# Colloid‐Mediated Synthesis of Hierarchically Porous Amorphous Catalyst for Durable Industrial‐Scale Water Electrolysis

**DOI:** 10.1002/adma.202516751

**Published:** 2025-12-29

**Authors:** Yu Liao, Lei Li, Jingxian Zhang, Yangyang Chen, Sha Luo, Yan Qing, Cuihua Tian, Guanjie He, Yiqiang Wu

**Affiliations:** ^1^ College of Materials and Energy Central South University of Forestry and Technology Changsha China; ^2^ Department of Chemistry University College London London UK

**Keywords:** anion exchange membrane water electrolysis, colloid‐mediated electroless plating, electrocatalyst, hydrogen evolution reaction, oxygen evolution reaction

## Abstract

Efficient and scalable hydrogen production via water electrolysis requires electrode architectures that combine high catalytic activity, effective active‐site utilization, and mechanical durability at industrial current densities. However, conventional synthesis routes often produce dense, fragile catalyst layers that limit performance and scalability. Herein, a colloid‐mediated electroless plating (CMEP) strategy is reported for the facile fabrication of hierarchically porous, amorphous Fe‐NiWB electrodes under ambient conditions. During CMEP, the in situ generation of Fe‐W‐O colloids suppresses compact layer growth, yielding an open architecture with abundant accessible sites, accelerated mass transfer, and strong substrate anchoring. Comprehensive structural and electronic analyses reveal that Fe incorporation modulates the local coordination environment, enhances intrinsic activity, and promotes beneficial dynamic surface reconstruction under alkaline oxygen evolution reaction (OER) conditions. The resulting electrode delivers excellent bifunctional activity and stability, sustaining 500 mA cm^−2^ for over 2000 h with negligible degradation in both hydrogen and oxygen evolution reactions (HER/OER). When integrated into an anion exchange membrane (AEM) electrolyzer, it delivers 500 mA cm^−2^ at 1.55 V with remarkable long‐term durability. A preliminary techno‐economic analysis (TEA) highlights the scalability and cost competitiveness of this approach, underscoring its promise for economically viable large‐scale green hydrogen production.

## Graphical abstract

A colloid‐mediated electroless plating (CMEP) strategy is proposed to fabricate hierarchically porous, amorphous Fe‐doped NiWB electrocatalysts under ambient conditions. The in situ formation of Fe‐W‐O colloidal species guides the formation of robust, porous catalyst layers with excellent mass transfer and durability, sustaining 500 mA cm^−2^ for 2000 h, showcasing a scalable, low‐cost platform for industrial hydrogen production via AEM electrolysis.

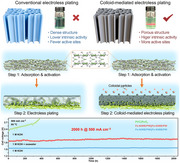

## 1 Introduction

Hydrogen (H_2_) plays a pivotal role in the decarbonization of industrial, transport, and energy systems [1, 2, 3]. Electrocatalytic water splitting using excess intermittent renewable energy sources such as solar, wind, and geothermal power offers a sustainable and efficient route for high‐purity H_2_ production [4, 5]. Among current electrolysis technologies, alkaline anion exchange membrane (AEM) water electrolyzers have emerged as a promising technology for the implementation of the oncoming hydrogen economy, as they combine the cost‐effectiveness and material flexibility of alkaline electrolysis with the high efficiency and compact design of proton exchange membrane (PEM) electrolyzers [6, 7]. Unlike PEM systems that rely on precious metals, AEM devices operate in a less corrosive alkaline environment and allow the use of non‐noble metal catalysts, reducing capital costs and expanding material choice [8, 9]. Despite these advantages, the scalable implementation of AEM electrolysis is still limited by the lack of highly active and durable non‐precious metal catalysts. Existing catalysts often exhibit low active site utilization and poor long‐term stability, especially under industrial conditions requiring high current densities (≥500 mA cm^−2^), elevated temperatures (50°C–80°C), prolonged operating periods, and efficient mass and charge transfer [10, 11].

Among non‐precious candidates, nickel‐based catalysts have garnered significant attention due to their favorable catalytic activity, affordability, and structural versatility [12, 13]. While certain advanced Ni‐based catalysts demonstrate promising activities, their long‐term durability under industrially relevant conditions remains a concern. The design of high‐performance catalysts thus demands simultaneous optimization of intrinsic electronic structures and external morphologies to accelerate kinetics and ensure mechanical integrity. In this context, amorphous materials, distinguished by their short‐range atomic order and structural flexibility, offer a higher density of active sites and dynamic surface reconfiguration capabilities compared to crystalline materials [14, 15, 16]. Nonetheless, conventional powdery catalysts typically require polymeric binders and metallic foam substrates for electrode assembly, which hinder ionic/gaseous transport, block active sites, and introduce mechanical instability due to bubble‐induced stress [17, 18]. Emerging designs inspired by natural structures, such as biomimetic porous arrays and anisotropic groove structures, have demonstrated improved catalytic efficiency [19, 20] but typically involve complex, energy‐intensive fabrication routes, impeding scalability. Similarly, conventional approaches to engineering porous architectures, such as hard/soft templating, dynamic hydrogen‐bubble templating, and dealloying, usually depend on multi‐step procedures that entail sacrificial templates, high‐current conditions, or corrosive etching. This reliance on demanding conditions emphasizes the need for a more straightforward and scalable fabrication strategy to achieve well‐defined porous architectures (Table S1).

To overcome these limitations, we developed a facile and scalable colloid‐mediated electroless plating (CMEP) method to fabricate amorphous Fe‐doped NiWB catalysts with a hierarchically porous architecture under ambient conditions. This approach leverages the in situ generation of Fe‐W‐O colloidal species to inhibit dense catalyst layer formation, yielding a well‐integrated porous coating with improved mass transfer properties and enhanced accessibility of active sites. The strong interfacial coupling between the catalyst and scaffold ensures excellent mechanical resilience under industrial current densities. Fe incorporation not only optimizes the electronic structure of the NiWB matrix, promoting charge transfer and lowering reaction barriers, but also facilitates surface reconstruction to active (Fe)NiOOH species under OER conditions. Benefiting from these synergistic enhancements, the Fe‐NiWB/PW electrode exhibits remarkable HER and OER performance in alkaline electrolyte, with low overpotentials (48 and 270 mV for HER; 248 and 350 mV for OER at 10 and 500 mA cm^−2^, respectively) and exceptional durability, maintaining 500 mA cm^−2^ for over 2000 h without noticeable degradation. When assembled into an AEM electrolyzer, the system delivers 500 mA cm^−2^ at a low cell voltage of 1.55 V with prolonged operational durability. Preliminary TEA indicates that an AEM electrolyzer plant employing Fe‐NiWB/PW can achieve hydrogen production cost targets set by the U.S. Department of Energy ($2.00/kg H_2_ by 2026) [21], reinforcing its potential for industrial‐scale green hydrogen production.

## 2 Results and Discussion

**FIGURE 1:**
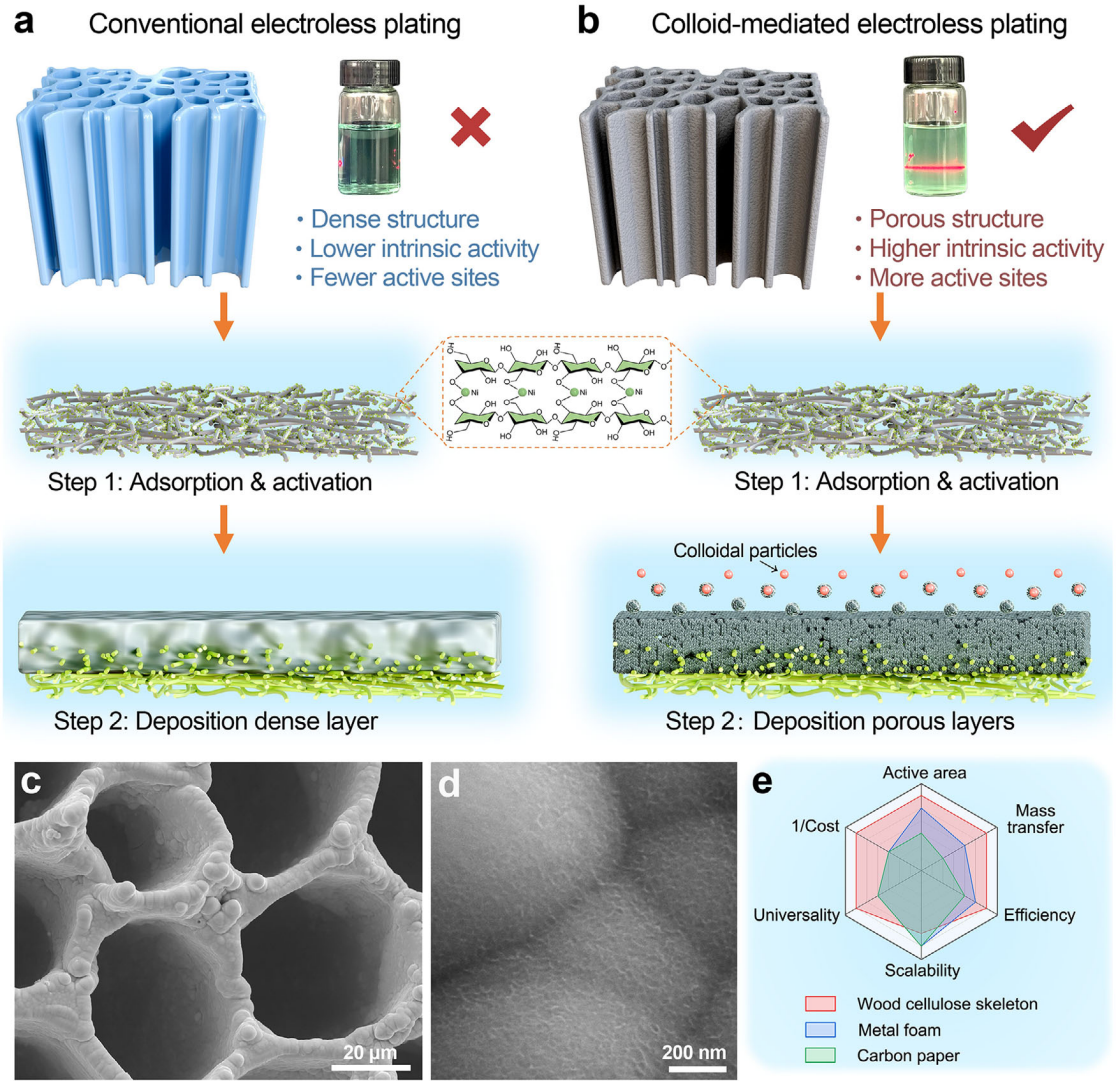
Synthesis and structural characterization. a, b) Schematic illustration of the fabrication process of the electrocatalyst via conventional electroless plating and colloid‐mediated electroless plating. c, d) SEM images of Fe‐NiWB/PW. e) Compared with commercial nickel foam and carbon paper without aligned microchannels, PW has a highly active surface area, low cost, efficient mass transfer behavior, outstanding efficiency, scalability, and universality in constructing electrode materials.

Electroless plating (EP) is widely valued for its simplicity, energy efficiency, and environmental compatibility, and has long been used in surface engineering. However, its application in electrocatalyst fabrication has been constrained by the dense and compact coatings typically produced via conventional EP methods, which limit catalytic activity due to restricted surface area and mass transfer (Figure 1a). To overcome this limitation, we developed a colloid‐mediated electroless plating (CMEP) strategy to directly fabricate an Fe‐doped Ni‐based amorphous catalyst on a purified wood (PW) skeleton featuring vertically aligned channels under ambient conditions (Figure 1b). The porosity and hydrophilicity of the PW skeleton were greatly improved after delignification, which facilitates subsequent uniform catalyst deposition (Figures S1 and S2). The plating process began with soaking the PW skeleton in NiSO_4_ solution to enable full absorption of Ni^2+^ ions, followed by activation in NaBH_4_ solution for 1 min, which reduced the Ni^2+^ to metallic nickel to serve as nucleation sites. A uniform, adherent catalyst layer was subsequently deposited via the CMEP process (Figure S3). Owing to its ambient‐condition operation, procedural simplicity, and inherent scalability, the CMEP strategy is ideally suited for large‐area catalyst fabrication. The resulting Fe‐NiWB/PW electrodes exhibit key advantages over state‐of‐the‐art counterparts, including higher active surface area, efficient mass transfer behavior, fast reaction kinetics, outstanding stability, and compatibility with scalable manufacturing (Figure 1e).

The morphological evolution of the catalyst layer during CMEP was systematically investigated using scanning electron microscopy (SEM). The pristine PW displayed a clean and smooth surface (Figure S1g,h). After 60 min of CMEP, a coherent catalyst layer comprising uniform nanospheres decorated with nanosheets was formed (Figure S4a–c). After 120 min, optimal microstructural features were achieved, with spherical porous nodules interconnected by nanosheets uniformly coating the substrate (Figure 1c,d; Figure S4e–g), creating a highly accessible, conductive architecture conducive to gas release and electrolyte diffusion. Extending the deposition to 180 min resulted in rough, aggregated particles (Figure S4i–k). Further prolongation to 240 min yielded a denser and less porous layer, which diminished active sites exposure and consequently degraded electrochemical performance (Figure S4m–o). Cross‐sectional SEM images confirmed the progressive thickening of the coating, which remained firmly anchored to the substrate, providing exceptional mechanical robustness under vigorous gas evolution conditions (Figure S4d,h,l,p).

To evaluate the generality of the CMEP method and the structural advantages of the PW scaffold, binder‐free Fe‐NiWB electrodes were also fabricated on alternative substrates, including filter paper (FP), melamine sponge (MS), and nickel foam (NF). SEM confirmed that CMEP enabled uniform catalyst deposition across all substrates, irrespective of their materials or geometries (Figures S5–S7). However, coatings on MS and NF showed weaker interfacial adhesion and were prone to delamination at high current densities (Figures S6 and S7), compromising stability. In contrast, the FP substrate, composed of cellulose fibers, offered stronger catalyst anchoring (Figure S5d). These observations underscore the importance of a hydrophilic, porous cellulose matrix in promoting stable and uniform catalyst deposition during CMEP (Figure S8).

**FIGURE 2:**
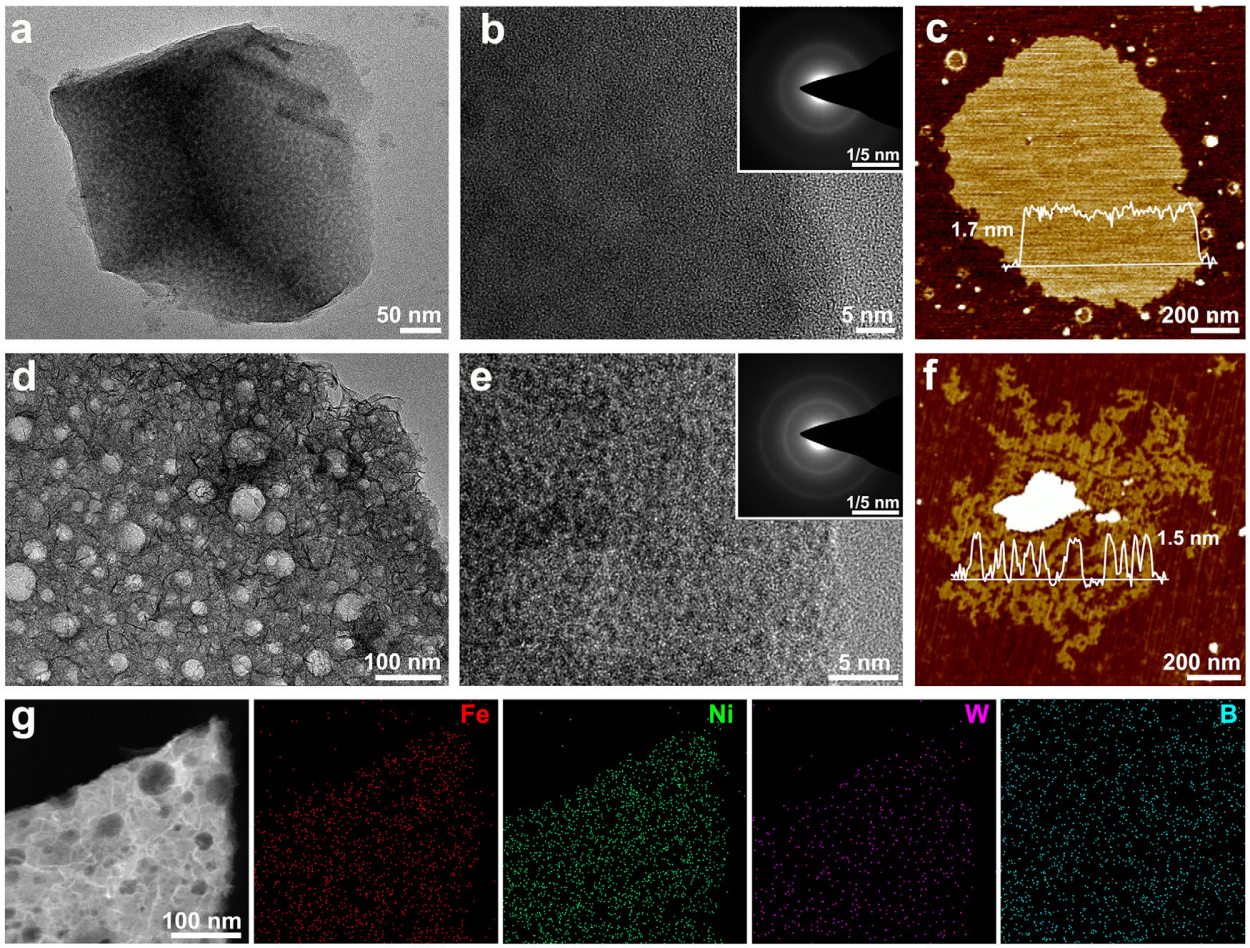
Morphological characterizations. a) TEM image, b) HRTEM image (inset is SAED pattern), and c) AFM image of NiWB. d) TEM image, e) HRTEM image (inset is SAED pattern), f) AFM image, and g) EDS elemental maps of Fe‐NiWB.

To elucidate the specific role of Fe, a control NiWB/PW sample (without Fe) was synthesized under identical conditions. After the optimal 120‐min deposition, NiWB/PW displayed a convex and rough surface lacking nanosheet structures (Figure S9). Transmission electron microscopy (TEM) and atomic force microscopy (AFM) further revealed that NiWB exhibited a relatively smooth layered morphology (Figure 2a–c), while Fe‐NiWB possessed a distinctly porous nanosheet architecture (Figure 2d–f). This structural distinction originates from the interaction of Fe^2+^ with WO_4_^2−^ in the plating bath, which leads to the in situ formation of insoluble Fe‐W‐O colloidal particles (Figures S10–S12). These particles become embedded within the growing catalyst matrix, thereby enhancing its porosity and increasing the density of exposed active sites (Figure S13). N_2_ adsorption‐desorption isotherms of the detached coatings revealed typical Type IV isotherms (Figure S14). The specific surface area of Fe‐NiWB (0.3494 m^2^ g^−1^) was approximately four times that of NiWB (0.0867 m^2^ g^−1^), corroborating the formation of an interconnected meso–macropores network induced by the Fe‐W‐O colloids (Table S2). High‐resolution TEM (HRTEM) images and the corresponding selected area electron diffraction (SAED) patterns confirmed the amorphous nature of both materials (Figure 2b,e). Elemental mapping demonstrated the homogeneous distribution of Fe, Ni, W, and B throughout the catalyst layer (Figure 2g), evidencing successful Fe incorporation and a uniform elemental composition. Inductively coupled plasma optical emission spectroscopy (ICP‐OES) quantified a major compositional shift from 80/6/14 (Ni/W/B) in NiWB/PW to 2/89/2/7 (Fe/Ni/W/B) in Fe‐NiWB/PW (Table S3), emphasizing the critical influence of Fe incorporation on the catalyst's architecture and its resulting properties.

X‐ray diffraction (XRD) patterns further confirmed the amorphous nature of both catalysts, with Fe‐NiWB/PW exhibiting slightly increased crystallinity due to Fe incorporation (Figure S15a). Raman spectroscopy showed only broad features associated with W─O─W, W═O/B─O, and Fe─O─W‐related vibrations (Figure S16). The absence of characteristic FeOOH bands confirms that Fe species are integrated into the amorphous NiWB matrix rather than forming separate Fe oxyhydroxide phases. X‐ray photoelectron spectroscopy (XPS) was employed to probe the chemical states and compositional differences between Fe‐NiWB and NiWB. As shown in Figure S15b, the Ni 2p spectra of Fe‐NiWB were deconvolved into Ni^2+^ (2p_1/2_ at 873.59 eV and 2p_3/2_ at 855.88 eV) and Ni^0^ (2p_1/2_ at 869.65 eV and 2p_3/2_ at 852.41 eV) [22]. Notably, the peaks of Ni in Fe‐NiWB shifted to higher binding energy compared with those in NiWB (873.48 and 855.80 eV for Ni^2+^; 869.40 and 852.25 eV for Ni^0^), indicating a slightly higher Ni valence state and suggesting electronic interaction between Ni and the incorporated Fe species. The W 4f spectra (Figure S15c) of both catalysts exhibit two prominent peaks around 34.97 and 37.11 eV, corresponding to the 4f_7/2_ and 4f_5/2_ of W^6+^ cations that dominate on the surface. Two smaller peaks at lower binding energy are associated with the metallic W, while a peak located at 41.36 eV represents the W loss feature [23, 24]. The B 1s spectrum (Figure S15d) shows the coexistence of metalloid B (187.12 eV) and oxidized B (191.95 eV, corresponding to borate) [16]. The Fe 2p spectrum of Fe‐NiWB (Figure S15e) reveals the presence of two oxidation states: Fe^3+^ and Fe^2+^. The Fe 2p_3/2_ and 2p_1/2_ for Fe^3+^ are located at 713.08 and 723.12 eV, respectively, while those for Fe^2+^ appear at 707.91 and 719.11 eV [25, 26], confirming the successful incorporation of Fe into the amorphous matrix.

To gain deeper insights into the atomic structure and electronic environment of Ni in both NiWB and Fe‐NiWB, X‐ray absorption spectroscopy (XAS) measurements were performed using NiO and Ni foil as references. As shown in Figure S15f, the normalized X‐ray absorption near‐edge structure (XANES) spectra of NiWB and Fe‐NiWB resemble that of Ni foil rather than NiO, confirming their predominantly metallic nature. Fe incorporation induces a slight shift of the Ni *K*‐edge toward higher energy, indicating an increase in the Ni oxidation state [27]. The slightly intensified pre‐edge shoulder in Fe‐NiWB suggests increased local distortion and decreased symmetry around Ni sites due to Fe incorporation [28]. Extended X‐ray absorption fine structure (EXAFS) analysis revealed both Ni─Ni and Ni─Fe bonds in Fe‐NiWB (Table S4), which is further supported by the Fourier‐transformed EXAFS spectra (Figure S15g), and the wavelet transforms of Ni *K*‐edge oscillations (Figure S15h). Additionally, EXAFS fitting of the R‐space spectra indicates a reduced Ni‐Ni coordination number and the emergence of a Ni‐Fe coordination shell in Fe‐NiWB (Figure S17 and Table S4), confirming successful Fe incorporation without altering the amorphous framework. Together, these results demonstrate that Fe doping effectively modulates both the local coordination environment and the electronic structure of Ni, creating a more favorable and stable catalytic environment.

The electrochemical performance of the Fe‐NiWB/PW electrode for both HER and OER was evaluated using a three‐electrode system in 1.0 m KOH at room temperature. Among electrodes fabricated with varying deposition durations (60–240 min), the one obtained after the 120‐min CMEP process exhibited the optimal activity for both reactions (Figure S18). This performance optimum correlates with its largest electrochemically active surface area (ECSA) and highest turnover frequency (TOF), confirming that a 120‐min process maximizes both the density of accessible active sites and the intrinsic activity of each site (Figure S19 and Table S5). To assess the generalizability of the CMEP method across different biomass substrates, Fe‐NiWB was also deposited on fast‐growing poplar and pine wood substrates. Although their performance was slightly lower than that of the balsa‐based Fe‐NiWB/PW, these electrodes exhibited uniform coatings and appreciable catalytic activity, confirming the versatility and scalability of the method (Figure S20).

**FIGURE 3:**
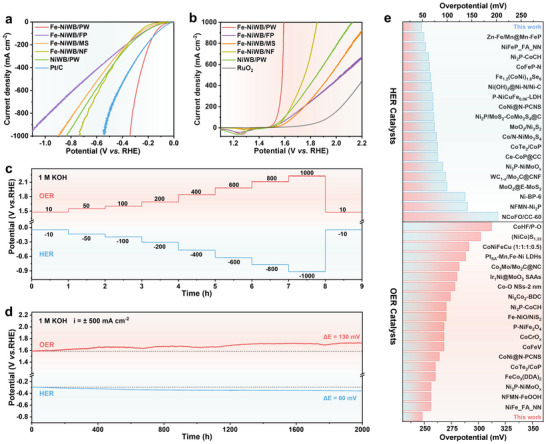
Electrocatalytic water splitting performance of the samples in 1.0 m KOH. a) HER, and b) OER polarization curves recorded in 1.0 m KOH. c) Multiple‐step chronopotentiometry (without iR correction) and d) long‐term chronopotentiometry measurement of Fe‐NiWB/PW for HER and OER. e) Comparison of overpotentials at 10 mA cm^−2^ with reported references in 1.0 m KOH.

The electrocatalytic activity of Fe‐NiWB/PW was further compared with that of Fe‐NiWB supported on NF, FP, and MS, as well as NiWB/PW, and commercial Pt/C and RuO_2_ catalysts. Among these, Fe‐NiWB/PW presented a rapid rise in current density with increasing overpotential for both HER and OER (Figure 3a,b), signifying superior catalytic activity. For HER, Fe‐NiWB/PW required only 48 mV to reach 10 mA cm^−2^, comparable to Pt/C (30 mV), and achieved 500 mA cm^−2^ at an overpotential of 270 mV— significantly lower than Pt/C (381 mV), NiWB/PW (549 mV), Fe‐NiWB/NF (532 mV), Fe‐NiWB/MS (588 mV), and Fe‐NiWB/FP (684 mV) (Figure S21a). A broader comparison with recently reported high‐efficiency HER electrocatalysts further highlighted the superior catalytic activity of Fe‐NiWB/PW at the 10 mA cm^−2^ benchmark (Figure 3e; Table S6). The Tafel slope analysis revealed that Fe‐NiWB/PW exhibited the smallest value (80.36 mV dec^−1^) among the tested samples (Fe‐NiWB/FP (86.12 mV dec^−1^), Fe‐NiWB/MS (109.00 mV dec^−1^), Fe‐NiWB/NF (88.99 mV dec^−1^), and NiWB/PW (103.65 mV dec^−1^), except for Pt/C (30.10 mV dec^−1^), indicating faster reaction kinetics, consistent with a Volmer‐Heyrovsky mechanism (Figure S21b) [29]. Electrochemical impedance spectroscopy (EIS) provided further support. The Nyquist plots showed that Fe‐NiWB/PW exhibited the smallest charge transfer resistance (Figure S21d), indicating that its vertically aligned microchannel architecture facilitates efficient charge transfer and accelerates the Faradaic process [30]. The catalyst also showed excellent durability under HER conditions. In a multi‐step chronopotentiometry test, overpotentials remained stable upon stepping to progressively higher current densities (Figure 3c), confirming mechanical and electrochemical robustness. Long‐term stability was assessed through chronopotentiometry at −500 mA cm^−2^, where the potential exhibited negligible fluctuation over 2000 h, with a decay rate of only 0.03 mV h^−1^ (Figure 3d).

For the OER, Fe‐NiWB/PW exhibited remarkable activity, achieving 10 and 500 mA cm^−2^ at low overpotentials of 248 and 350 mV, respectively. These values are significantly lower than those for RuO_2_ (𝜂10 = 426 mV), Fe‐NiWB/FP (274 mV), Fe‐NiWB/MS (309 mV), Fe‐NiWB/NF (281 mV), and NiWB/PW (262 mV) (Figure 3b; Figure S21a). Notably, at industrially relevant current densities, Fe‐NiWB/PW reached 1000 mA cm^−2^ at an overpotential of just 363 mV, outperforming most reported state‐of‐the‐art OER catalysts (Figure 3e; Table S7). The Tafel slope for Fe‐NiWB/PW (42.59 mV dec^−1^) was the lowest among all tested samples, indicating faster OER kinetics compared to Fe‐NiWB/FP (53.25 mV dec^−1^), Fe‐NiWB/MS (95.70 mV dec^−1^), Fe‐NiWB/NF (80.93 mV dec^−1^), NiWB/PW (49.76 mV dec^−1^), and RuO_2_ (281.93 mV dec^−1^) (Figure S21c). These activity trends were further corroborated by EIS results, with Fe‐NiWB/PW consistently showing the lowest charge transfer resistance (Figure S21e). Apart from its high activity, stability tests under OER conditions demonstrated consistent activity across a range of current densities (10–1000 mA cm^−2^) (Figure 3c). At 500 mA cm^−2^, Fe‐NiWB/PW maintained stable performance over 2000 h with a decay rate of only 0.07 mV h^−1^ (Figure 3d), highlighting its long‐term durability in alkaline conditions.

The ECSA, determined from double‐layer capacitance, was the highest for Fe‐NiWB/PW (15.39 mF cm^−2^), indicating a greater density of active sites compared to Fe‐NiWB/FP (14.29 mF cm^−2^), Fe‐NiWB/MS (5.48 mF cm^−2^), Fe‐NiWB/NF (3.59 mF cm^−2^), and NiWB/PW (14.78 mF cm^−2^) (Figure S22). ECSA‐normalized polarization curves further demonstrated that Fe‐NiWB/PW exhibited the highest intrinsic catalytic activity (Figure S23). These results underscore the role of Fe in enhancing charge transfer and increasing active site density, thereby contributing to superior HER and OER performance.

To understand the catalytic active sites of the Fe‐NiWB/PW catalyst, we conducted a comprehensive analysis of its microstructure, surface composition, and chemical state evolution during OER and HER tests. XRD patterns revealed negligible changes after prolonged electrolysis, indicating excellent bulk structural stability (Figures S24a and S30a). After the OER tests in 1.0 m KOH, the nanosheet morphology on the Fe‐NiWB/PW surface remained intact, with more pronounced growth (Figure S25a,b). High‐resolution XPS analysis revealed the oxidation of surface Ni to higher valence states, evidenced by the disappearance of Ni^0^ 2p peaks and the emergence of Ni^3+^ 2p peaks (Figure S24b) [28]. HRTEM and SAED images (Figure S25e,f) further confirmed the formation of nickel (oxy)hydroxides, which act as the primary active species for OER catalysis. Time‐resolved ICP‐OES analysis (Figure S26) showed that W and B undergo a rapid yet self‐limiting leaching during the initial hour of operation, after which their concentrations stabilize. This early‐stage leaching creates a more open near‐surface structure, which facilitates Ni oxidation and active‐phase generation. Interestingly, minimal leaching of Ni and Fe occurred during HER. In contrast, during OER, Fe ions underwent a dynamic dissolution‐redeposition process, indicating a surface equilibrium that prevents continuous material loss, consistent with the stable catalytic activity observed [31, 32, 33]. Collectively, these results suggest that Fe‐NiWB undergoes controlled and beneficial surface reconstruction rather than undesirable structural degradation during electrolysis. This adaptive transformation is key to its exceptional long‐term durability in alkaline electrolytes.

**FIGURE 4:**
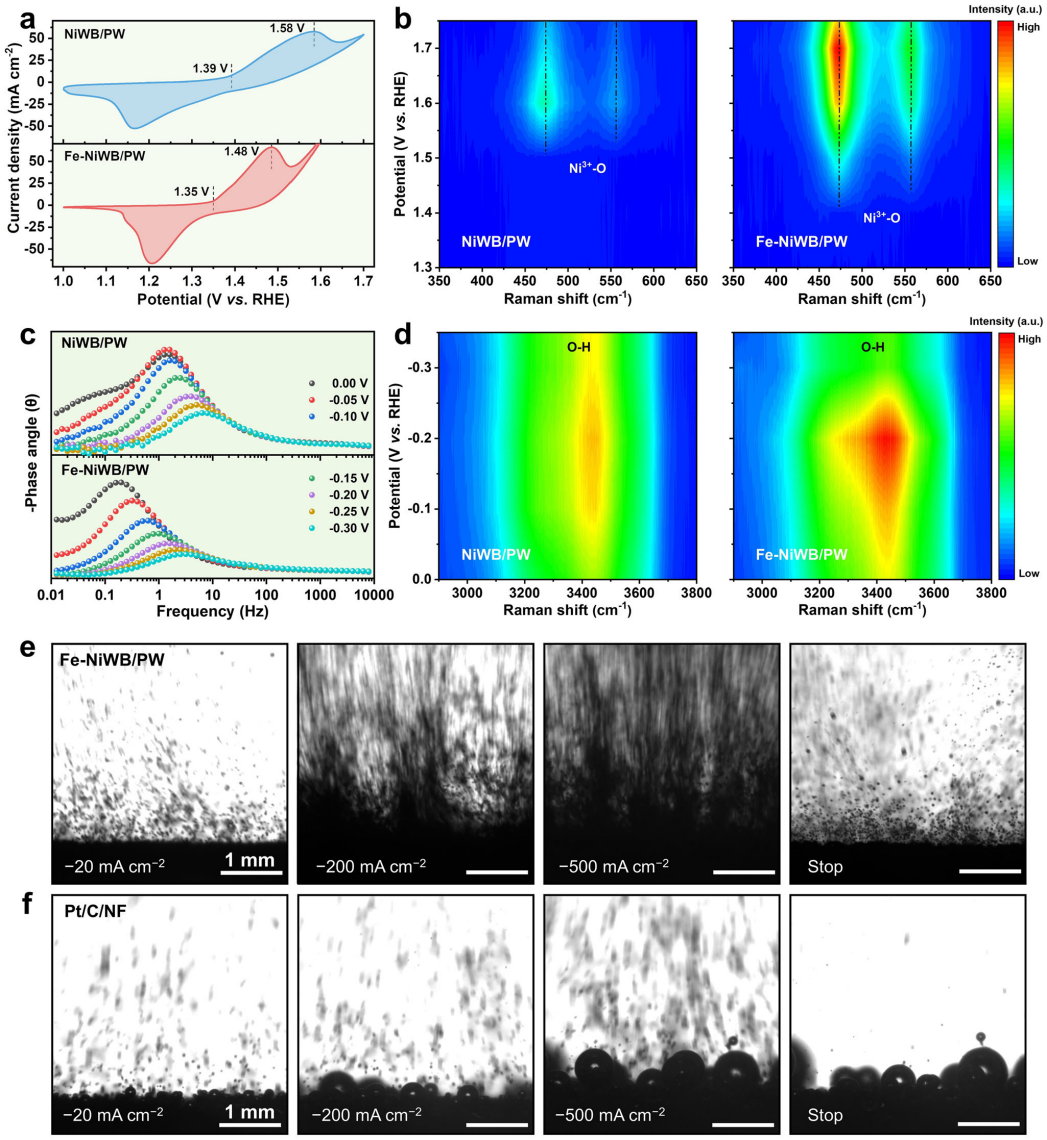
Structural evolution and mass transfer behavior analysis. a) Cyclic voltammetry curves (scan rate: 1 mV s^−1^, without iR compensation) and b) in situ Raman spectra of NiWB/PW and Fe‐NiWB/PW during OER. c) In situ Bode plots and d) in situ Raman spectra of NiWB/PW and Fe‐NiWB/PW during HER. e, f) Images of hydrogen bubbles released from Fe‐NiWB/PW and Pt/C/NF by using a high‐speed camera during chronopotentiometry measured under different current densities.

Electrochemical analysis further highlighted the critical role of Fe in enhancing OER activity. Cyclic voltammetry showed that the onset potential for Ni oxidation is approximately 100 mV lower for Fe‐NiWB/PW than for NiWB/PW (Figure 4a), indicating that Fe doping significantly facilitates the electrochemical formation of NiOOH at reduced overpotentials. This observation was corroborated by in situ Raman spectroscopy, which revealed the emergence of characteristic Ni^3+^─O vibrational modes (470 and 560 cm^−1^) associated with NiOOH [34] at 1.40 V for Fe‐NiWB/PW, compared to 1.50 V for NiWB/PW (Figure 4b; Figure S27). Notably, the absence of Fe‐specific Raman signals suggests that Fe is homogeneously incorporated into the NiOOH lattice [35], thereby reinforcing the reconstruction process and stabilizing the catalytically active (Fe)NiOOH phase.

During HER, the Fe‐NiWB/PW catalyst retained its nanosheet morphology, with only minor changes induced by alkaline leaching that did not compromise performance (Figure S28a–c). In situ Raman spectroscopy detected no significant structural changes during short‐term HER operation (Figure S29). XPS analysis revealed progressive surface oxidation after HER, evidenced by attenuated W and B signals and the loss of Ni^0^ peaks (Figure S30). However, no significant shift in Fe or Ni binding energies was observed, confirming the stability of their valence states during HER. HRTEM and SAED further indicated a partial transformation of the near‐surface region into low‐crystallinity nickel hydroxides (Figure S28e,f). These results indicate that Fe‐NiWB acts as the active phase for HER. Although surface evolution occurred after prolonged alkaline exposure, HER performance remained stable.

Operando EIS provided detailed insights into the structure–activity relationships and charge transfer kinetics [29]. In Bode plots, the low‐frequency and high‐frequency regions correspond to interfacial charge transfer and inner‐layer electron transport, respectively [36]. The Bode plots revealed a marked decrease in the low‐frequency phase angle for Fe‐NiWB/PW under HER conditions, reflecting enhanced interfacial charge transfer that accelerates the HER kinetics (Figure 4c) [36]. Consistently, Nyquist plots showed smaller semicircles for Fe‐NiWB/PW, indicating improved reactant adsorption and faster Heyrovsky step kinetics compared with NiWB/PW (Figure S31) [37]. Under OER conditions, Fe‐NiWB/PW similarly displayed lower charge transfer resistance and pronounced low‐frequency phase shifts, highlighting its superior charge transfer capability, which contributes to its enhanced OER kinetics (Figure S32).

To elucidate the mechanism underlying the enhanced HER performance of Fe‐NiWB/PW, in situ Raman spectroscopy was employed to probe interfacial water dynamics in 1.0 m KOH (Figure S33). Both catalysts exhibited broad O─H stretching bands in the range of 3100–3650 cm^−1^, corresponding to interfacial water adsorption [38]. The peaks at ∼3260 and 3460 cm^−1^ are assigned to four hydrogen‐bonded water (4‐HB·H_2_O), and two hydrogen‐bonded water (2‐HB·H_2_O), respectively, while the peak at ∼3610 cm^−1^ is attributed to K^+^ hydrated water (K^+^·H_2_O) [39, 40]. At 0 V (vs. RHE), Fe‐NiWB/PW exhibited more pronounced O─H vibrational intensities, indicating enhanced interfacial water adsorption. Upon applying cathodic potential, these vibrational features attenuated more rapidly for Fe‐NiWB/PW, reflecting faster water consumption and more favorable kinetics for the Volmer step. In contrast, NiWB/PW showed both weaker initial O─H signals and a slower rate of intensity decay during HER, consistent with its less efficient activation and cleavage of interfacial water. This contrast is visually apparent in the potential‐dependent Raman contour plots (Figure 4d), which collectively underscore the more effective interfacial water recruitment and superior catalytic efficiency of Fe‐NiWB/PW. Taken together, these findings demonstrate that Fe incorporation boosts both OER and HER performance by facilitating favorable electrochemical reconstruction, accelerating charge transfer kinetics, and optimizing interfacial water dynamics.

Mass transfer at the electrode–electrolyte interface, including the flow of liquid‐phase reactants and the evolution of gas‐phase products, is essential for enhancing electrochemical gas evolution reactions, especially at high current densities [41]. The rapid detachment of gas bubbles from the electrode surface is particularly critical, as it maintains active site availability and enhances overall energy efficiency [42, 43]. To assess the advantage conferred by the hierarchically porous structure and oriented open channels of the Fe‐NiWB/PW electrode, contact angle measurements were performed to examine its surface wettability. A droplet of 1.0 m KOH solution was completely absorbed by Fe‐NiWB/PW within 62 ms, demonstrating its superhydrophilicity (Figure S34a). In contrast, the less hydrophilic Fe‐NiWB/MS and Fe‐NiWB/NF required 124 ms for full absorption (Figure S34b,c), while Fe‐NiWB/FP exhibited a large contact angle of 116°, indicating poor wettability and an inability to absorb the droplet (Figure S34d). These results underscore the superhydrophilicity of the Fe‐NiWB/PW electrode, which facilitates electrolyte accessibility–a crucial factor for efficient gas evolution reactions [44].

To further elucidate how the aligned, multichannel PW scaffold governs bubble dynamics, bubble behavior during HER and OER was monitored using a high‐speed camera (Figure S35). On the Fe‐NiWB/PW electrode, hydrogen nucleated as uniformly dispersed microbubbles that detached rapidly from the surface, indicating pronounced superaerophobicity. At 500 mA cm^−2^, bubble evolution intensified, with the surface becoming densely populated by rapidly detaching microbubbles, reflecting the enhanced gas‐release dynamics at high current densities. Strikingly different behavior was observed on Pt/C/NF, where bubble removal was sluggish: bubbles adhered, coalesced into larger ones, and persisted even after the applied bias had been removed (Figure 4e,f; Movie S1). Such persistent bubble adhesion can block active sites, increase interfacial resistance, and potentially cause mechanical degradation under industrial operating conditions. Other control substrates, including Fe‐NiWB/NF, Fe‐NiWB/MS, and Fe‐NiWB/FP, also exhibited stronger bubble adhesion than Fe‐NiWB/PW during both HER and OER (Figures S36 and S37).

The exceptional bubble detachment capability of Fe‐NiWB/PW stems from the intrinsic structural merits of the PW scaffold. Its vertically aligned, low‐tortuosity channels, coupled with strong superhydrophilicity, promote rapid electrolyte infiltration and capillary‐driven replenishment. This ensures a continuous supply of water while efficiently evacuating generated gas bubbles. As illustrated in Figure S38, this ordered multiscale architecture disrupts the three‐phase‐contact (TPC) interface, lowers bubble adhesion energy, and accelerates bubble desorption. Conversely, the disordered macropores in NF and MS tend to trap gas bubbles, leading to coalescence and pore blockage that interrupts electrolyte flow [17]. Meanwhile, the flat FP substrate lacks the necessary surface roughness to break the TPC interface, resulting in persistent bubble adhesion. Overall, the superwetting properties and unique structural features of Fe‐NiWB/PW maximize active site utilization, ensure unhindered electrolyte penetration, and enable efficient gas product release. These synergistic attributes fundamentally explain the electrode's outstanding performance and durability at high current densities.

**FIGURE 5:**
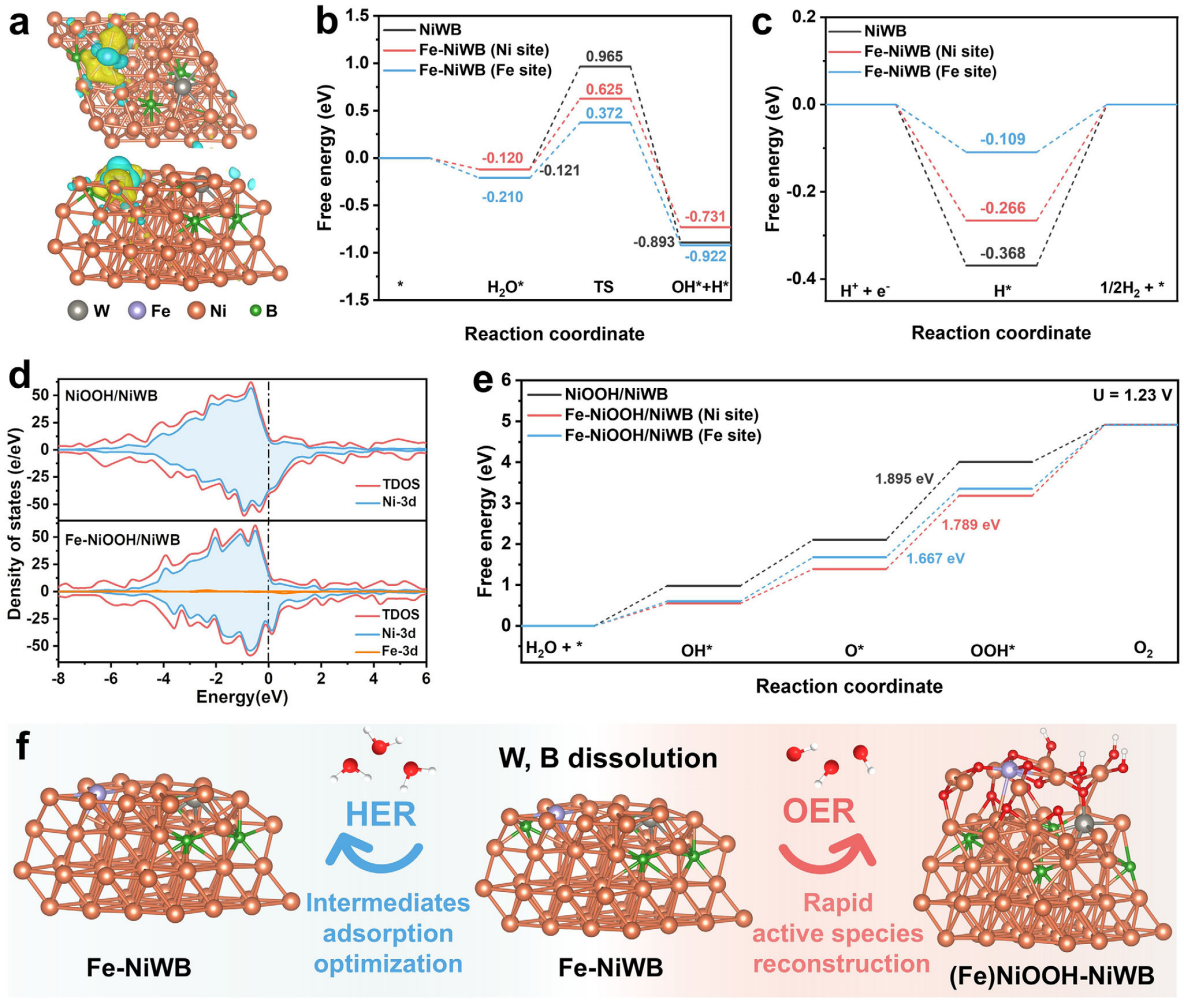
Theoretical investigations. a) Charge density difference of the Fe‐NiWB. b) Kinetics barriers of water adsorption and dissociation path on NiWB, Fe‐NiWB (Ni site), and Fe‐NiWB (Fe site), respectively. c) The calculated adsorption free energy of hydrogen on NiWB, Fe‐NiWB (Ni site), and Fe‐NiWB (Fe site), respectively. d) Density of state (DOS) analysis for the NiOOH/NiWB and Fe‐NiOOH/NiWB. e) Gibbs free energy diagram of OER on NiOOH/NiWB and (Fe)NiOOH/NiWB. f) Schematic representation of the genuine phases and active sites of Fe‐NiWB in 1.0 M KOH electrolyte during HER and OER.

To clarify the role of Fe incorporation in boosting the HER and OER performance of Fe‐NiWB/PW, density functional theory (DFT) calculations were carried out. Schematic models for HER (Fe‐NiWB and NiWB) and OER (NiOOH‐NiWB and (Fe)NiOOH‐NiWB) were constructed based on experimental findings (Figure S39). The charge density difference of Fe‐NiWB (Figure 5a) shows electron accumulation near Fe sites (yellow regions) and electron depletion (blue regions), indicating pronounced electron rearrangement due to Fe incorporation. Complementary electron localization function (ELF) analysis (Figure S40) reveals that Fe induces a more homogeneous electron distribution on adjacent Ni sites than in pristine NiWB. Such moderated local electron localization suggests an electronically optimized environment that more readily accommodates the adsorption and transformation of reaction intermediates. In addition, ELF analysis indicates that B strengthens electronic localization around Ni─B bonds, helping to stabilize adsorbed intermediates, whereas W primarily consolidates the amorphous framework and introduces local disorder that increases accessible active sites. Together, Fe, W, and B collaboratively modulate electronic distribution and structural robustness, creating an optimized electronic environment for catalysis. The total density of states (TDOS) further supports the enhanced conductivity of Fe‐NiWB, as evidenced by its *d*‐band center (−0.908 eV) being closer to the Fermi level than that of NiWB (−0.997 eV) (Figure S41 and Table S8).

The HER process in alkaline electrolytes typically involves water dissociation, hydrogen intermediate adsorption, and hydrogen generation. To explore this mechanism, we calculated the HER pathways on Fe‐NiWB and NiWB. Differential charge density analysis (Figure S42) reveals stronger interactions between Fe sites and H_2_O molecules, accompanied by electron transfer from Fe centers to the O atoms in H_2_O. Quantitatively, Fe sites donate 0.024 e^−^ to adsorbed H_2_O (*vs*. 0.004 e^−^ at Ni sites in NiWB), thereby enhancing water activation efficiency. Similarly, Fe sites provide 0.285 e^−^ to adsorbed H* intermediates (*vs*. 0.250 e^−^ at Ni sites in NiWB), yielding an optimized H* binding environment that facilitates subsequent desorption. As shown in Figure 5b, Fe sites in Fe‐NiWB exhibit a more negative H_2_O adsorption energy, promoting stronger water adsorption. Notably, the water dissociation energy at Fe sites in Fe‐NiWB is only 0.582 eV, substantially lower than the values for Ni sites in Fe‐NiWB (0.745 eV) or NiWB (1.086 eV). This lower barrier implies accelerated water dissociation and Heyrovsky‐step kinetics (Figures S43–S45) [45]. Additionally, the free energy of H* adsorption (ΔG_H*_) is a key descriptor for HER activity. On Fe sites in Fe‐NiWB, ΔG_H*_ is 0.109 eV, near the thermoneutral value (Figure 5c), indicating that Fe sites act as ideal proton acceptors, facilitating both H* conversion and H_2_ desorption [40].

For OER, the electronic structure of the reconstructed catalysts was evaluated by analyzing their d‐band centers. As shown in Figure 5d, the Ni *d*‐band center is −1.120 eV in NiOOH/NiWB and remains nearly unchanged (−1.117 eV) in (Fe)NiOOH/NiWB, whereas Fe sites exhibit a much deeper *d*‐band center of −1.454 eV. This lower Fe *d*‐band center implies stronger electronic interaction with oxygen intermediates. Free‐energy diagrams (Figure 5e) show that the formation of *OOH from *O is the rate‐determining step (RDS) for both systems (Figures S46–S48). For NiOOH/NiWB, the RDS free‐energy barrier is 1.895 eV. Upon Fe incorporation, this barrier decreases markedly: 1.789 eV at Ni sites and only 1.667 eV at Fe sites in (Fe)NiOOH/NiWB. Thus, the Fe site provides the most energetically favorable pathway for OER, requiring the lowest free energy for O─O bond formation. This reduction in the RDS free‐energy barrier provides a direct theoretical explanation for the enhanced OER activity following Fe incorporation, as observed experimentally.

Integrating the experimental and theoretical findings, the proposed reaction mechanism is summarized in Figure 5f. The hierarchically porous nanosheet structure of Fe‐NiWB, along with W and B leaching during electrocatalysis, increases the electrochemical surface area, exposes abundant active sites, and enhances charge and mass transfer. Concurrently, Fe incorporation optimizes the local electronic structure, tunes intermediate adsorption energies, and accelerates elementary reaction kinetics. These combined effects underpin the superior bifunctional HER/OER performance of Fe‐NiWB.

**FIGURE 6:**
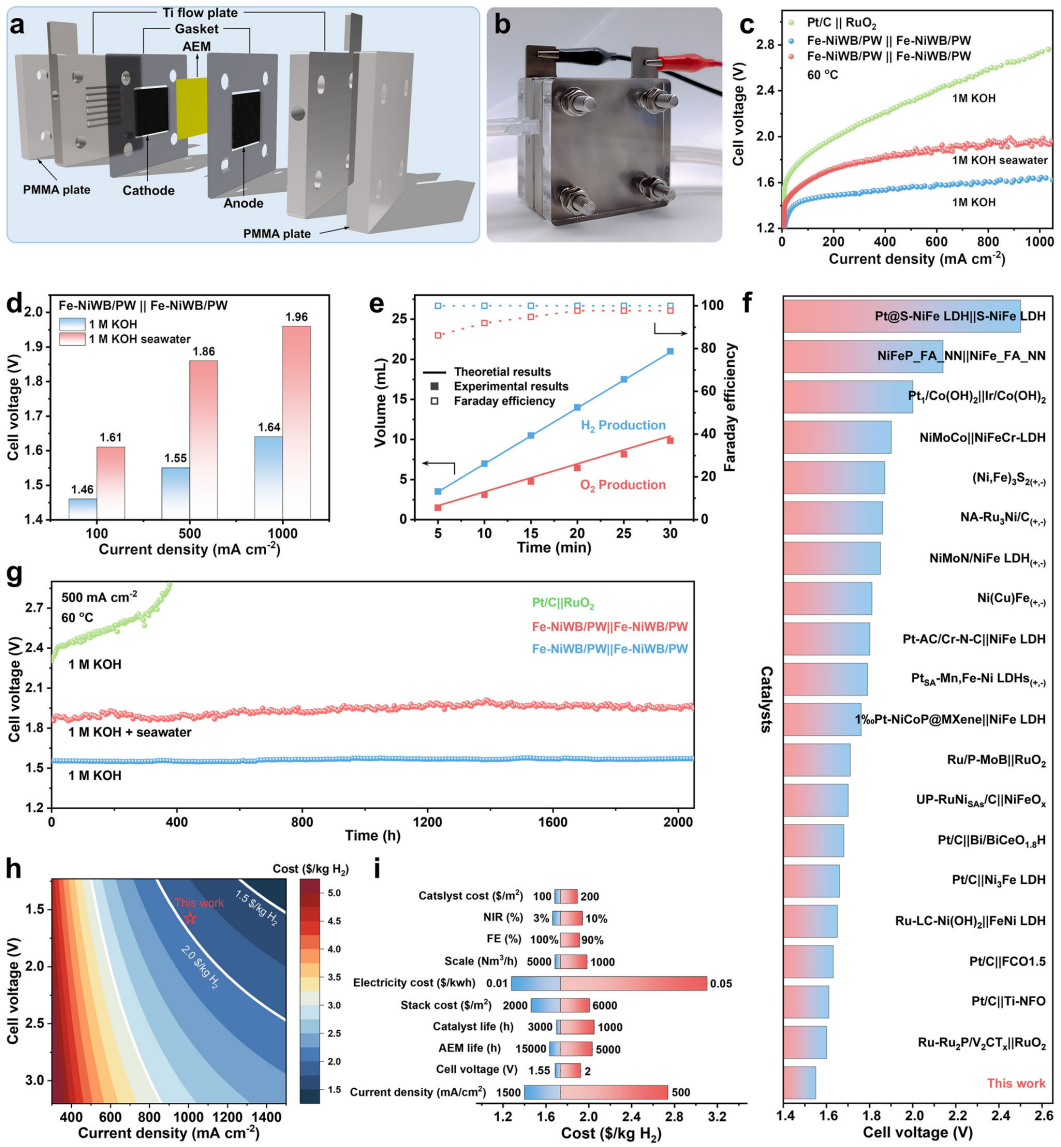
Water splitting application and cost analysis. a) Schematic diagram and b) picture of the Anion Exchange Membrane (AEM) electrolyzer. c) LSV curves of Fe‐NiWB/PW||Fe‐NiWB/PW in 1.0 M KOH and 1.0 M KOH seawater with Pt/C||RuO_2_ as a comparison. d) Cell voltages for water electrolysis on Fe‐NiWB/PW||Fe‐NiWB/PW at 100, 500, and 1000 mA cm^−2^ in 1.0 m KOH and 1.0 m KOH seawater, respectively. e) The Faradaic efficiency and gas production of Fe‐NiWB/PW. f) Comparison of total water splitting performance between Fe‐NiWB/PW and reported catalysts. g) Chronopotentiometry curve of Fe‐NiWB/PW||Fe‐NiWB/PW. h) Plant operating current density and cell voltage dependence, and i) Sensitivity analysis of hydrogen levelized production cost for an ideal 1 MW‐based AEM plant.

Building on the exceptional bifunctional activity of Fe‐NiWB/PW in a three‐electrode system, we assembled an AEM electrolyzer with Fe‐NiWB/PW serving as both the cathode and anode to assess its practical application performance (Figure 6a,b). Remarkably, this AEM electrolyzer delivered a current density of 500 mA cm^−2^ at a cell voltage of just 1.55 V in 1.0 m KOH (Figure 6c), surpassing the performance of a commercial Pt/C||RuO_2_ benchmark system. Gas quantification using the drainage method confirmed nearly 100% Faradaic efficiency (FE) of H_2_ production, while O_2_ FE reached 86% initially due to charge consumption during anodic reconstruction into (Fe)NiOOH, as confirmed by in situ Raman spectroscopy. Following this reconstruction, the O_2_ FE increased to and remained stable above 98% (Figure 6e; Figure S49), consistent with the complete formation of the active phase. The carbon‐free, binder‐free architecture, together with the high FEs for both gases, underscores efficient charge utilization and negligible side reactions during overall water splitting.

Motivated by the potential cost benefits of seawater electrolysis, we further evaluated the Fe‐NiWB/PW couple in an AEM electrolyzer using 1.0 m KOH + seawater electrolyte (Figures S50 and S51). In this medium, it achieved a current density of 500 mA cm^−2^ at 1.86 V (Figure 6d). Long‐term stability tests demonstrated that Fe‐NiWB/PW maintained stable operation for over 2000 h at 500 mA cm^−2^ in both 1.0 m KOH and alkaline seawater, with no significant voltage increase (Figure 6g). In contrast, the Pt/C||RuO_2_ couple showed a drastic increase in cell voltage within 400 h, primarily due to the catalyst peeling caused by accumulated gas bubble shock. As summarized in Figure 6f and Table S9, the Fe‐NiWB/PW electrode exhibits superior long‐term durability and activity compared with most previously reported catalysts. This exceptional bifunctional performance establishes Fe‐NiWB/PW as a promising and cost‐effective alternative for efficient water splitting. Additionally, the potential of intermittent renewable‐energy‐driven water electrolysis using Fe‐NiWB/PW was explored. The electrolyzer successfully operated with solar, wind, and thermal energy inputs, generating abundant gas bubbles on both electrodes (Figure 6h; Figure S52). Its ability to maintain operation under fluctuating voltage conditions underscores its adaptability to diverse energy inputs, highlighting strong practical potential.

Compared with commercial noble‐metal benchmarks such as Pt/C and RuO_2_, the Fe‐NiWB/PW offers substantial cost advantages (Tables S10 and S11). To quantitatively assess its economic viability, we performed a preliminary techno‐economic analysis (TEA) to estimate the levelized cost of hydrogen (LCOH) produced by an ideal 1 MW‐scale AEM electrolysis plant utilizing the single cell technology developed here (Figure S53; Tables S12–S18). Assuming continuous operation at 1 A cm^−2^ over a 20‐year plant lifetime, the system achieves green hydrogen production at a competitive LCOH of US$1.74 per kg H_2_, already below the U.S. Department of Energy's 2026 target (< $2 per kg H_2_) [21]. Sensitivity analysis identifies electricity price and operating current density as the dominant factors influencing LCOH (Figure 6i), reinforcing that pairing high‐performance catalysts with low‐cost renewable electricity is crucial for cost‐competitive hydrogen production. These results highlight the techno‐economic competitiveness of Fe‐NiWB/PW and its promise as a scalable, durable, and economically viable catalyst system for next‐generation green hydrogen production.

## 3 Conclusions

In summary, we have developed a colloid‐mediated electroless plating (CMEP) strategy for the ambient‐conditions fabrication of hierarchically porous, amorphous Fe‐doped NiWB electrocatalysts. The in situ formation of Fe‐W‐O colloidal species during plating plays a pivotal role in regulating catalyst morphology, effectively suppressing the growth of dense layers and yielding an open architecture that promotes mass transfer and increases the density of electrochemically accessible sites. Combined experimental and theoretical analyses reveal that Fe incorporation modulates the local coordination environment, promotes favorable electrochemical surface reconstruction, and enhances the intrinsic activity of catalytic sites. Operando and post‐reaction analyses further reveal that the catalyst undergoes a dynamic transformation into active (Fe)NiOOH during OER, while maintaining an amorphous phase during HER, resulting in exceptional bifunctional activity and long‐term durability at industrial current densities. Consequently, the Fe‐NiWB/PW electrode delivers outstanding performance in a practical AEM water electrolyzer, sustaining industrial‐level current densities (500 mA cm^−2^) for over 2000 h with negligible degradation. This work not only offers fundamental insights into catalyst structure–function relationships but also establishes CMEP as a versatile and scalable strategy for designing cost‐effective, large‐area electrocatalysts, presenting a promising pathway toward sustainable hydrogen production.

## 4 Experimental Section

Experimental details are provided in the Supporting Information.

## Conflicts of Interest

The authors declare no conflicts of interest.

## Supporting information




**Supporting file**: adma71994‐sup‐0001‐SuppMat.docx

**Supporting file**: adma71994‐sup‐0002‐MovieS1.mp4

## Data Availability

The data that support the findings of this study are available from the corresponding author upon reasonable request.
