# Supplementary material for: Colloid‐Mediated Synthesis of Hierarchically Porous Amorphous Catalyst for Durable Industrial‐Scale Water Electrolysis
Source: Adv Mater. 2025 Dec 29;38(10):e16751. doi: 10.1002/adma.202516751 (PMC12910545; doi:10.1002/adma.202516751)
Supplement: Supplementary file 1 — Supporting file: adma71994‐sup‐0001‐SuppMat.docx [file ADMA-38-e16751-s001.docx]

Copyright WILEY-VCH Verlag GmbH & Co. KGaA, 69469 Weinheim, Germany, 2018.

Supporting Information

**Colloid-Mediated Synthesis of Hierarchically Porous Amorphous Catalyst for Durable Industrial-Scale Water Electrolysis**

*Yu Liao, Lei Li, Jingxian Zhang, Yangyang Chen, Sha Luo, Yan Qing*, Cuihua Tian, Guanjie He* & Yiqiang Wu**

Y. Liao, L. Li, J. Zhang, Y. Chen, S. Luo, Y. Qing, C. Tian, Y. Wu

College of Materials and Energy, Central South University of Forestry and Technology, Changsha 410004, China.

E-mail: qingyan@csuft.edu.cn; wuyiqiang@csuft.edu.cn

G. He

Department of Chemistry, University College London, London WC1H 0AJ, UK.

E-mail: g.he@ucl.ac.uk

**Experimental Section**

**Chemical Reagents and Raw Materials:** Balsa wood (*Ochroma lagopus* Swartz) was obtained from Zhuhai Yoongsun Century Trading Co., Ltd. (Zhuhai, China). The following reagents were purchased from Sinopharm Chemical Regent Co., Ltd. (Shanghai, China): sodium hypochlorite solution (NaClO, available Cl ≥ 15%), nickel sulfate (NiSO_4_•6H_2_O, 98.5%), sodium sulfate (Na_2_SO_4_, 99.0%), sodium succinate (C_4_H_4_Na_2_O_4_•6H_2_O, 99.0%), sodium tungstate (Na_2_WO_4_•6H_2_O, 99.5%) and ammonium ferrous sulfate ((NH_4_)_2_Fe(SO_4_)_2_•6H_2_O, 99.5%). Dimethylamine borane (DMAB, 96.0%) and RuO_2_ (99.9%) were sourced from Shanghai Macklin Biochemical Technology Co., Ltd. (Shanghai, China). Sodium borohydride (NaBH_4_, 98.0%) was supplied by Chengdu Kelong Chemical Co. Ltd. (Chengdu, China). 20 % Pt/C (Hispec3000) was purchased from Johnson Matthey (London, UK). Distilled water was obtained from Guangzhou Watsons Food and Beverage Co., Ltd. (Guangzhou, China). All chemicals and reagents were used as received without further purification.

**Preparation of Purified Wood:** The Balsa wood block was first cut into 1 mm thick pieces perpendicular to its growth direction. These slices were treated with an aqueous NaClO solution at 25 ℃ for 30 minutes, then thoroughly washed with water and ethanol. The resulting purified wood (PW) scaffolds were stored in 50% ethanol until use.

**Fabrication of Fe-NiWB/PW electrode:** To activate the substrate, the PW was rinsed with water, blotted dry, and soaked in a 0.5 M nickel sulfate solution for 4 hours. Afterward, the nickel-loaded PW was activated in a 0.2 M NaBH_4_ solution for 1 minute to reduce the adsorbed Ni^2+^ to metallic nucleation sites, followed by rinsing with water.

For the colloid-mediated electroless plating solution, accurately weigh the mass of each component required for 1L plating bath (sodium succinate 25 g, Nickel sulfate 24.5 g, sodium sulfate 15 g, sodium tungstate 1.5 g, Dimethylamine borane 7.2 g, and ammonium ferrous sulfate 0.5 g) pour it into the beaker and add the proper amount of water to dissolve it. The dissolved component solution was mixed under constant stirring, and the final volume was adjusted to 1 L. The activated PW was then submerged in the plating solution at room temperature for 120 minutes. After plating, the Fe-NiWB/PW electrode was washed with water and ethanol and dried at room temperature under vacuum. The effect of plating time on catalyst formation was investigated by varying the plating duration to 60, 180, and 240 minutes.

The Fe-NiWB/FP, Fe-NiWB/MS, and Fe-NiWB/NF electrodes were prepared using Filter paper (FP), Melamine sponge (MS), and Nickel foam (NF) substrates, using the identical CMEP procedure.

**Fabrication of the NiWB/PW electrode:** The NiWB/PW electrode was prepared analogously, omission of ammonium ferrous sulfate and adjusting the nickel sulfate quantity to 25 g.

**Preparation of Benchmark Electrodes:** To prepare the Pt/C and RuO_2_ electrodes for comparison, 10 mg of commercial 20 wt.% Pt/C or RuO_2_ was dispersed in a mixture of 700 μL water, 260 μL isopropanol, and 40 μL Nafion. The dispersion was sonicated for 60 minutes to achieve homogeneity. The resulting ink was then drop-cast onto nickel foam (1.0 mm thick) and air-dried to achieve a catalyst loading of 1.0 mg cm^−2^.

**Materials Characterization:** The morphology of the materials was observed using a field emission scanning electron microscope (SEM) (Zeiss Sigma 300, Germany), and transmission electron microscopy (TEM) (FEI Tecnai F20, USA). Samples were sputter-coated with a thin layer of platinum by Oxford Quorum SC7620 (England) before SEM characterization. X-ray diffraction (XRD) patterns were obtained with a Panalytical Empyrean powder diffractometer (Netherlands), using Cu Kα radiation source (λ=0.15406 nm) at 40 kV and 40 mA with a scan rate of 5° min^−1^. X-ray photoelectron spectroscopy (XPS) was performed using a Thermo Scientific K-Alpha instrument (USA) with an Al Kα radiation source (*hv* =1486.6 eV), with the C 1s peak fixed at 284.8 eV for calibration. Contact angles were measured at room temperature using an OCA-15 instrument (Data-physis, Germany). The size and zeta potential of colloidal particles were analyzed using a Zetasizer Nano ZS instrument (MAIVERN, England).

**XAFS measurements:** Ni K-edge analysis was conducted at the BL20U beamline at the Shanghai Synchrotron Radiation Facility (SSRF) using a Si (311) crystal monochromator. Powder samples were pressed into thin discs (1 cm in diameter). X-ray absorption fine structure (XAFS) spectra were recorded in transmission mode at room temperature using a Bruker 5040 Silicon Drift Detector (SDD). Both Ni K-edge extended X-ray absorption fine structure (EXAFS), and Ni K-edge X-ray absorption near-edge structure (XANES) spectra were analyzed. Standard samples (Ni foil and NiO) were also recorded in transmission mode for comparison. Data processing and fitting were carried out using the Athena and Artemis software packages.

**ICP-OES Analyses of Ion Dissolution:** To determine the ion leaching during HER and OER processes, Fe-NiWB/PW electrode (1 × 1 cm) was operated at constant current (−500 for HER and 500 mA cm^−2^ for OER) in 70 mL of 1 M KOH. At predetermined intervals (1, 12, 24, 48, and 72 hours), 1 mL of electrolyte were extracted, diluted to 50 mL with water, and analyzed for ion leaching using inductively coupled plasma optical emission spectroscopy (ICP-OES, Agilent 7700(MS), USA).

**In-situ Raman measurements:** In-situ Raman spectroscopy was performed using a LabRAM Odyssey confocal microscope Raman system (Horiba, Japan) with a 532 nm laser excitation. The electrode surface changes during HER (0 to −0.5 V vs RHE) and OER (1.2 to 1.8 V vs RHE) were monitored in 1 M KOH. The laser power was set to 1.7 mW, and the spectral range was 50 to 4000 cm^−1^. Raman spectra were recorded over 3-minute acquisition periods using chronoamperometry at the specified potential.

**Electrochemical Measurements**: Electrochemical tests were performed using an Interface 1000T (Gamry Instruments, USA) in a typical three-electrode setup at 25 ℃. The working electrode was the prepared self-supported electrode (1 cm^2^ work area), with a graphite rod, and Hg/HgO electrode serving as the counter, and reference electrodes, respectively. An N_2_-saturated 1.0 M KOH solution was applied as the electrolyte. All potentials were referenced to the reversible hydrogen electrode (RHE) using the conversion: E_RHE_= E_Hg/HgO_ + 0.059 pH+0.098 V. Linear sweep voltammetry (LSV) was conducted at a scan rate of 1 mV s^−1^ to minimize capacitance currents. To eliminate the incorrect identification of OER overpotential caused by metal oxidation peaks, the OER polarization curves were collected from high to low potential. The Tafel slope (b) was extracted from the linear portion of the polarization curves using the equation: *η* = *b log(j)* + *a*, where *η* is the overpotential, *a* is the Tafel constant, and *j* is the current density. Electrochemical impedance spectroscopy (EIS) measurements were taken across a frequency range of 0.01 and 10^5^ Hz. Double-layer capacitance (*C*_dl_) measurements within the range of 0.6~0.7 V (vs RHE) were conducted by cyclic voltammetry (CV) at scan rates of 10, 20, 40, 60, 80, and 100 mV s^−1^. By plotting the capacitive current (Δ*j*/2 at 0.65 V *vs.* RHE) against the CV scan rates, the *C*_dl_ was obtained as the slope of the corresponding linear. The electrochemically active surface area (ECSA) was calculated using: ECSA = *C*_dl_/*C*_s_, where C_s_ = 0.04 mF cm^−2^. Long-term stability was assessed by chronopotentiometry at a constant current density. Unless otherwise specified, all electrochemical data presented in this work were processed using an 80% iR compensation according to E_iR_ = E_tested_ – I * 0.8 R_s_, where R_s_ is the system resistance obtained from high-frequency EIS.

Faradic efficiency (FE) was determined by measuring the total charge passed during the electrocatalytic reaction and the amount of produced gas n _H2/O2_ (unit: mol). The experiment was conducted in a two-electrode cell system at a constant current of 100 mA for 30 minutes, and the produced gas volume was collected by the water drainage method. FE was calculated using the equation:

$$\mathrm{FE}\left( \% \right)=\frac{96485\times Z\times n \left( H_{2}/O_{2} \right)\times100\%}{Q}$$

Where *Z* is the number of electrons required to generate one molecule of H_2_ (*Z* = 2) or O_2_ (*Z* = 4), n represents the amount of H_2_/O_2_ produced (mol), and Q is the total charge passed.

**Alkaline anion exchange membrane (AEM) water electrolysis test**: The AEM (FAB-PK-130, Fumasep, Germany) was used with the Fe-NiWB/PW electrode as both the anode and cathode. Electrically insulating gaskets were used to prevent leakage of liquid or gas. The AEM electrolyzer was compressed using four bolts to ensure tight seals. The electrochemical performance of the AEM water electrolyzer, with an active area of 1 cm², was evaluated by LSV in a 1.0 M KOH solution circulated by a peristaltic pump. The electrolyte temperature was maintained at 60 ℃ using a water bath, and the potential was scanned from 3.0 to 1.2 V at a scan rate of 1 mV s^−1^. Long-term stability was tested at a constant current density of 500 mA cm^−2^.

**DFT calculations:** Density functional theory (DFT) calculations were carried out using the Vienna Ab-initio Simulation Package (VASP) ^[1]^, within the generalized gradient approximation (GGA) using the Perdew-Burke-Ernzerhof (PBE) formulation ^[2]^. The projected augmented wave (PAW) method ^[3]^ was used for ionic core description, and valence electrons were treated with a plane-wave basis set with a kinetic energy cutoff of 450 eV. Partial occupancies of the Kohn−Sham orbitals were allowed using the Gaussian smearing method and a width of 0.05 eV. Electronic energy convergence was set to 10⁻^5^ eV, and geometry optimization was considered convergent when the energy change was smaller than 0.05 eV/Å. A 2 × 2 × 1 Monkhorst mesh was used to sample the Brillouin zone ^[4]^. To account for solvent effects, the VASPsol implicit-solvation model was employed, which introduces a self-consistent continuum dielectric environment to simulate the aqueous electrolyte (dielectric constant ε = 78.4).

The slab models were constructed with a 15Å vacuum layer to prevent interactions between periodic images. Amorphous NiWB was simulated using ab initio molecular dynamics (AIMD) to accurately capture its atomic-level structure. Following the AIMD simulation, a simulated annealing process was employed to refine the amorphous bulk structure, which was maintained under the NVT (constant number of particles, volume, and temperature) conditions for a duration of 15PS. For the slab model, the [111] crystallographic direction of the amorphous bulk was selected to align with the surface normal.

The Gibbs free energy (ΔG) can be obtained by adding corrections including entropic (*TS*) and zero-point energy (ZPE) to the calculated DFT energy so that ΔG = Δ*E*_DFT_ + Δ*ZPE* – *T*Δ*S* – e*U*. where the E_DFT_ is the calculated DFT reaction energy, Δ*ZPE* is the change in ZPE calculated from the vibrational frequencies, and T is the temperature (T=298.15 K), Δ*S* is the change in the entropy referring to thermodynamic databases. The electrode potential is adopted concerning the reversible hydrogen electrode, which makes the standard electrochemical potential of the electron involved in the reaction (*G*_e_) equal to – e*U*, and the standard electrochemical potential of the proton (*G*_H+_) equal to that of the hydrogen atom in gaseous H_2_ (1/2*G*H_2_). Considering that the triplet state of the O_2_ molecule is poorly described in the current DFT scheme, the free energy of the O_2_ molecule was derived according to G_O2_ = 2G_H2O_ – 2G_H2_ + 4.92.

**Techno-Economic Analysis**

**Analysis Methods and Indicators:** Techno-economic analysis employs the cash flow method (CF). Dynamic economic evaluation indicators consider the time value of money and analyze the entire economic data set of revenues and expenditures over the project’s lifetime. Therefore, dynamic evaluation indicators are more comprehensive and scientific than static indicators. The primary dynamic evaluation indicators include the Return on Investment (ROI), Net Present Value (NPV), Payback Period (PBP), and Internal Rate of Return (IRR).

**Return on Investment (ROI):** ROI is an indicator used to evaluate the return on a project investment. It calculates the ratio of investment returns to investment costs. A higher ROI generally indicates better economic feasibility.

$ROI=\frac{\sum_{t=1}^{N} (GP_{t}-Taxespaid_{t})}{N\cdot CAPEX}*100\%$

Where *GP-Taxes paid* represents the total annual revenue after tax, *N* denotes the plant life, and *CAPEX* is the total investment cost.

**Net Present Value (NPV)**: NPV analysis is used to compute the difference between the discounted future cash flows of a project and its investment costs. A positive NPV signifies that the net revenue of the project exceeds its investment cost.

$NPV(n)=\sum_{\text{t=0}}^{n} \frac{CF(t)}{(1+NIR)^{t}}$

Where *n* represents the operational year. NPV is computed based on cash flow (*CF*(*n*)) ^[5]^, assuming a one-year construction period. The cash flow calculation as follows:

$CF(n)=\left\{ \begin{matrix} CAPEX,n=0 \\ GP,n=1 \\ GP-IT(GP_{n-1}-D_{n-1}),n\geq2 \\ WC+GP-IT(GP_{n-1}-D_{n-1}),n=N \end{matrix} \right.$

Where *IT(GP_n-1_ – D_n-1_)* is the tax from the previous year, and *D* is the depreciation expense.

**Payback Period (PBP)**: PBP refers to the time required to recover the total investment from the project's net income (annual revenue minus annual expenditure) from the commencement of investment. It is determined by the point where *NPV* = 0. Using the cash flow method, the PBP can be determined when *NPV*(*n*) ＜ 0, and *NPV*(*n+1*) ＞ 0 can be calculated more precisely using interpolation ^[6]^ as:

$PBP=n+\frac{|NPV(n)|}{CF_{present}(n+1)}$

Where *CF*_present_ is the present value of cash flow, calculated as:

$CF_{present}(n)=\frac{CF(n)}{(1+NIR)^{n}}$

**Internal Rate of Return (IRR)**: IRR is the discount rate at which the cumulative present value of net cash flows over the project’s life cycle equals zero, i.e., *NPV*(*N*) = 0:

$NPV(N)=\sum_{\text{t=0}}^{N} \frac{CF(t)}{(1+IRR)^{t}}=0$

The IRR is an efficiency indicator that reflects the profitability of the funds utilized in the project. Unlike NPV, which requires a predetermined discount rate, the IRR is an intrinsic measure derived from the project's cash flow characteristics. Determining the benchmark rate of return is challenging, as national guidelines exist for certain industries, but many sectors still lack reference benchmarks.

**Analytical Procedure**: The annuity techno-economic assessment method is used to estimate costs ^[7]^. Each year’s capital investment cost is assumed to be borrowed and repaid at a specified loan interest rate over the plant’s life cycle. The annualized investment cost (ACC) is calculated as:

$ACC=FCI\times\frac{NIR\times(1+NIR)^{N}}{(1+NIR)^{N}-1}$

Total production cost is determined as follows:

$C_{total}=\frac{(ACC+CCOP)}{Annual Production}$

The total system cost comprises CAPEX and OPEX. CAPEX includes equipment procurement, construction costs, and contingency expenses, whereas OPEX covers labor costs, maintenance expenses, material and utility costs, and administrative overheads. The cost analysis in this study follows the framework established in references ^[8]^. A life cycle cost assessment is conducted, incorporating expenses associated with the investment phase, operational phase, and decommissioning phase. It is assumed that working capital accounts for 5% of total capital costs, while the salvage value of the facility is set at 0%.

**Supporting Figures**


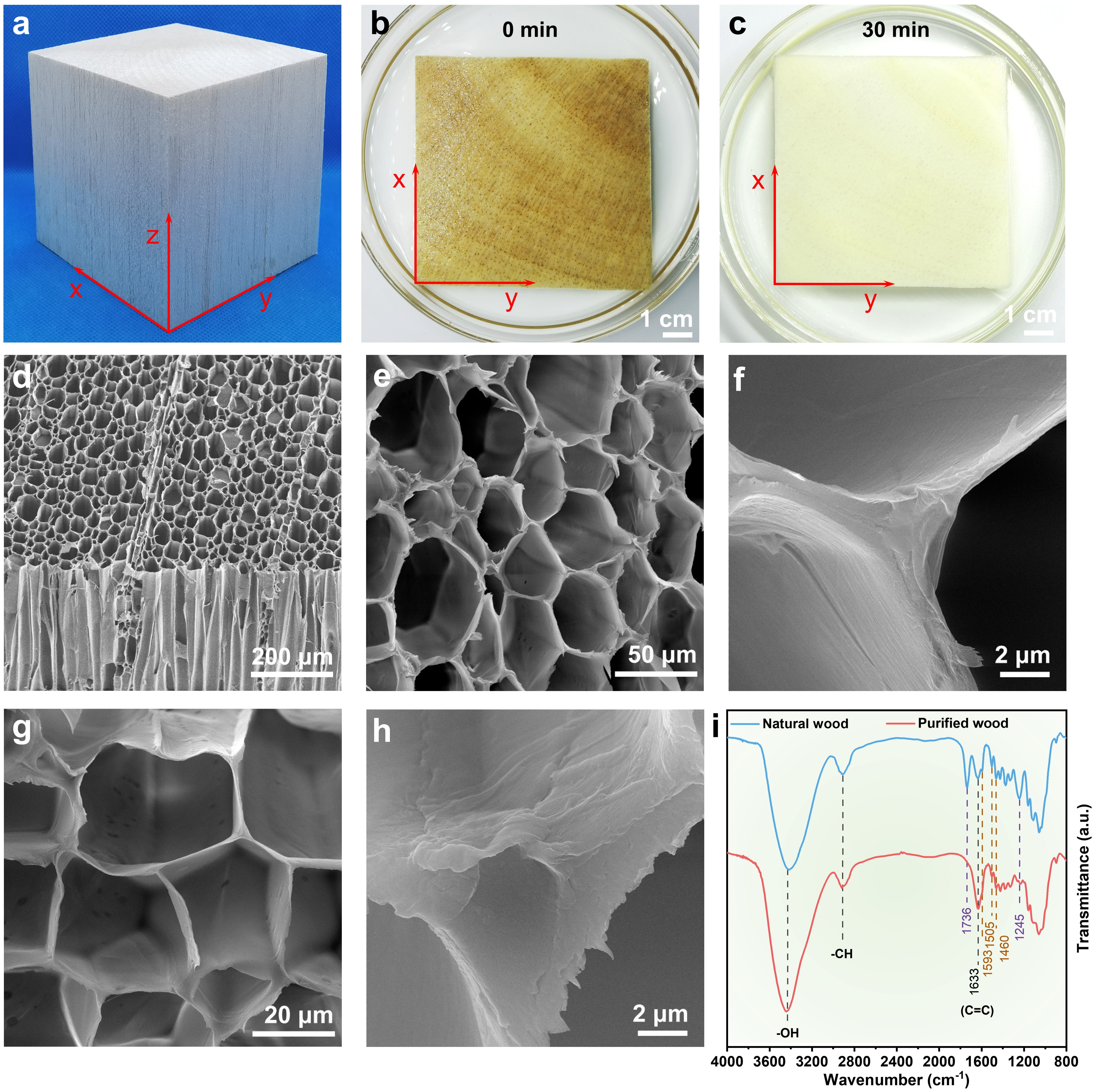


**Figure S1.** a) Potograph of natural blasa wood block. b, c) Color changes of wood slices before and after the delignification process. SEM images of d, e, f) natural wood and g, h) purified wood. i) FTIR spectra of natural and purified wood.

**Note:** A simple chemical treatment using an aqueous NaClO_2_ solution was employed to selectively remove the lignin and hemicellulose from the wood, thereby enhancing its hydrophilicity and porosity. After treatment, the originally yellowish balsa wood slice became completely white, indicating the effective removal of dark-colored lignin and the retention of colorless polysaccharides (Figure S1a-c). After oxidation, the wood cell-wall structure loosened, the inter-fiber pit apertures opened, and the pore network became markedly more accessible (Figure S1d-h). In the Fourier transform infrared (FT-IR) spectrum of the purified wood, the characteristic lignin peaks at 1593, 1505, and 1460 cm^−1^ (aromatic skeletal vibrations) decrease after the chemical treatment. The absorption at 1736 cm^−1^ corresponds to the carbonyl stretching vibration in hemicellulose. The band of 1245 cm^−1^ is associated with uronic acid groups in hemicellulose or ester linkages originating from lignin and hemicellulose (Figure S1i) ^[9]^. The disappearance of the 1736 cm^−1^ band, together with reduced intensities of the 1245 cm^−1^ band and other lignin-related peaks, indicates partial dissolution/removal of lignin and hemicellulose from the natural wood after treatment.


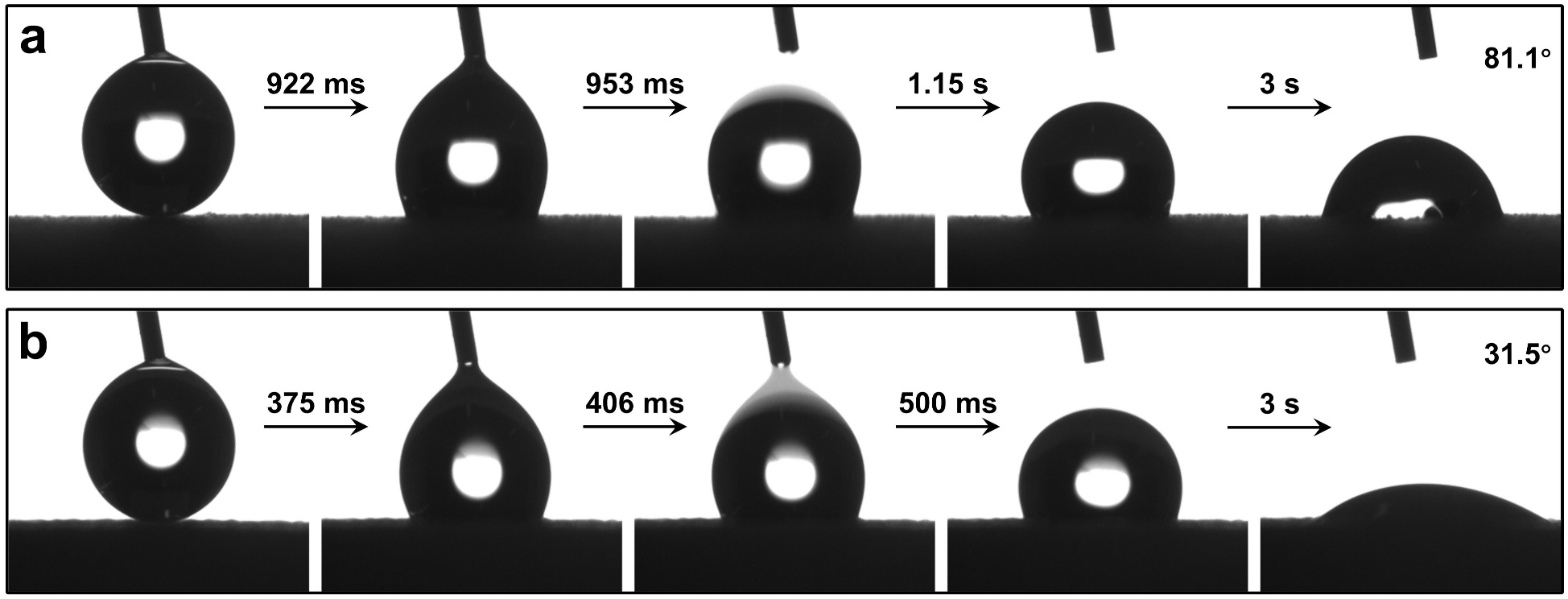


**Figure S2.** Dynamic wetting images of a) natural balsa wood and b) purified balsa wood.


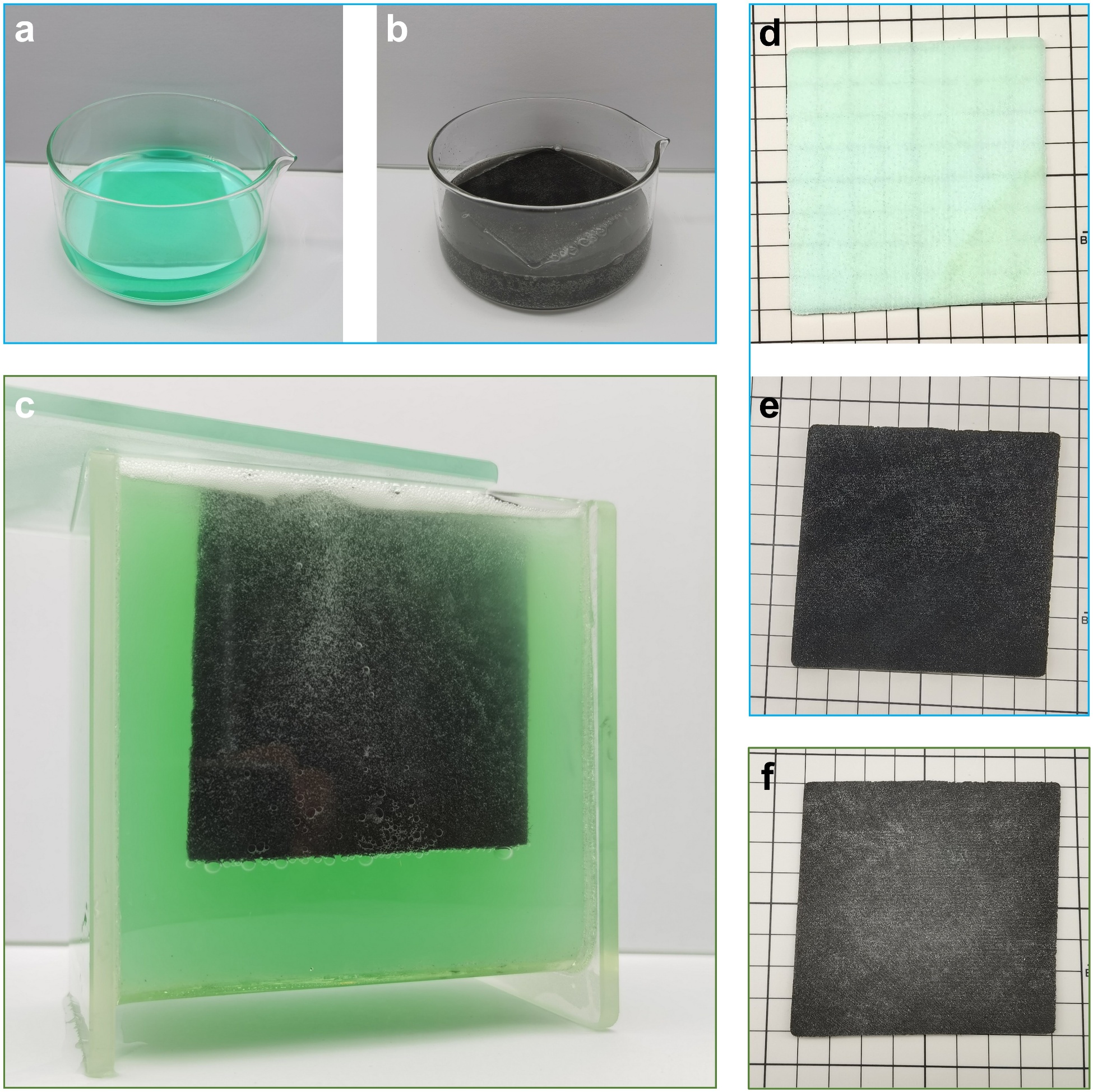


**Figure S3.** Optical photos of the catalytic material preparation process and samples at each stage. a) Soaked the PW in a nickel sulfate solution, b) then dipped in a NaBH_4_ solution for activation. c) Finally, the colloid-mediated electroless plating process was performed. d-f) The samples after the treatment of steps a), b), and c), respectively. (Each square has an area of 1 cm^2^)


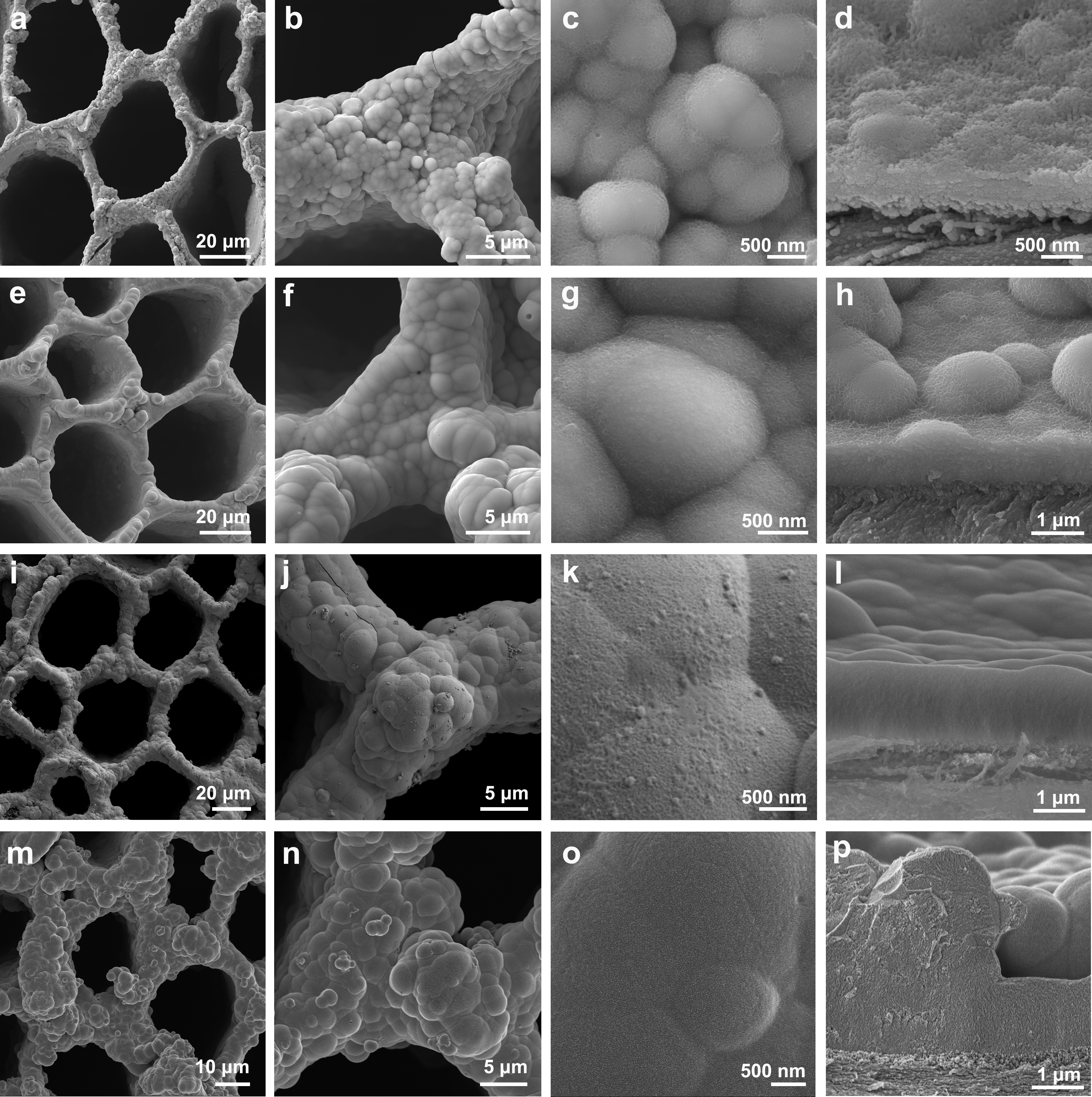


**Figure S4.** Top-view and cross-sectional SEM images of Fe-NiWB/PW with different synthesis times of a-d) 60 min, e-h) 120 min, i-l) 180 min, and m-p) 240 min.


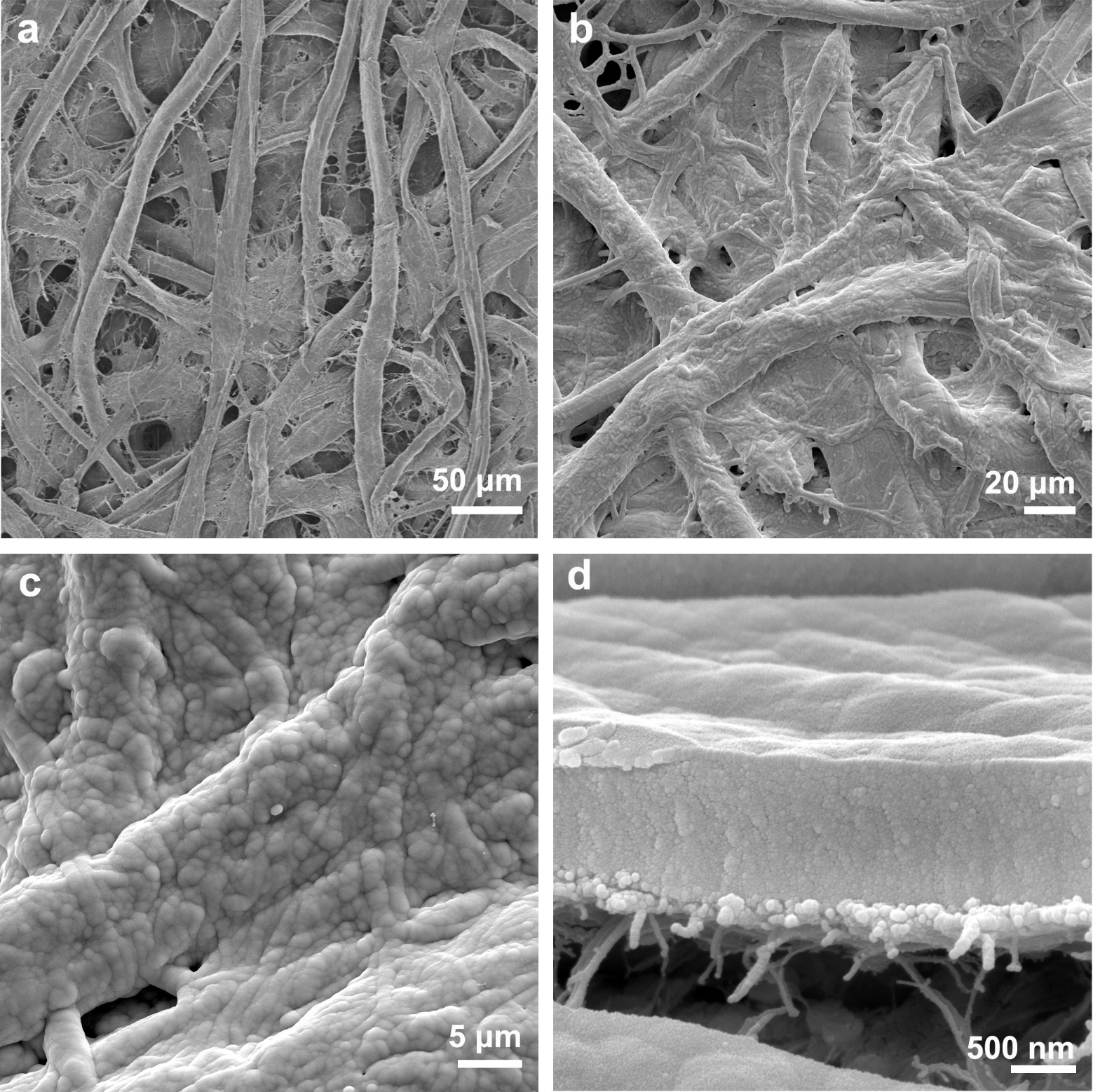


**Figure S5.** Top-view SEM images of a) Filter paper, and b, c) Fe-NiWB/FP. d) Cross-sectional SEM images of Fe-NiWB/FP.


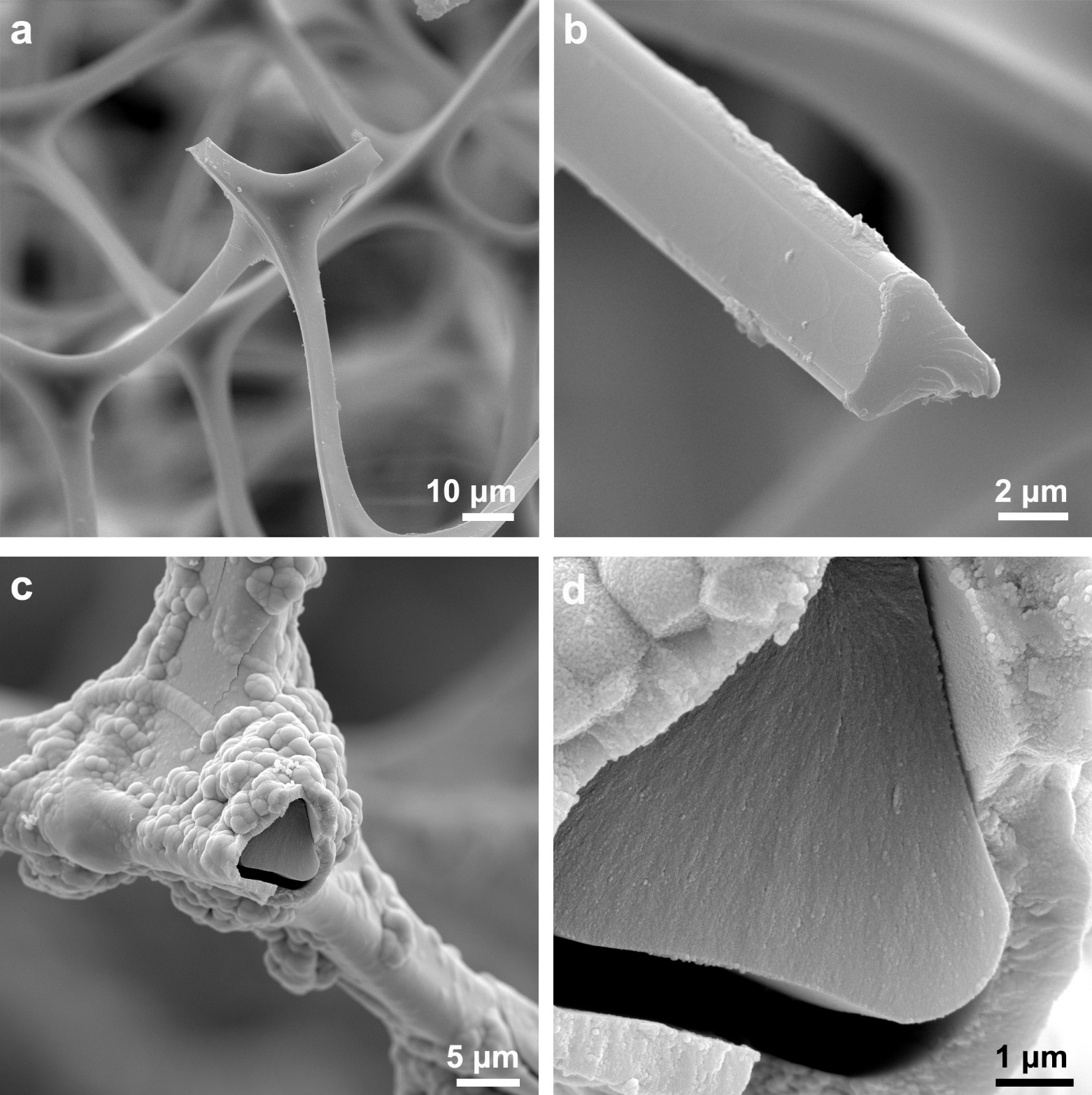


**Figure S6.** SEM images of a, b) Melamine sponge , and c,d) Fe-NiWB/MS.


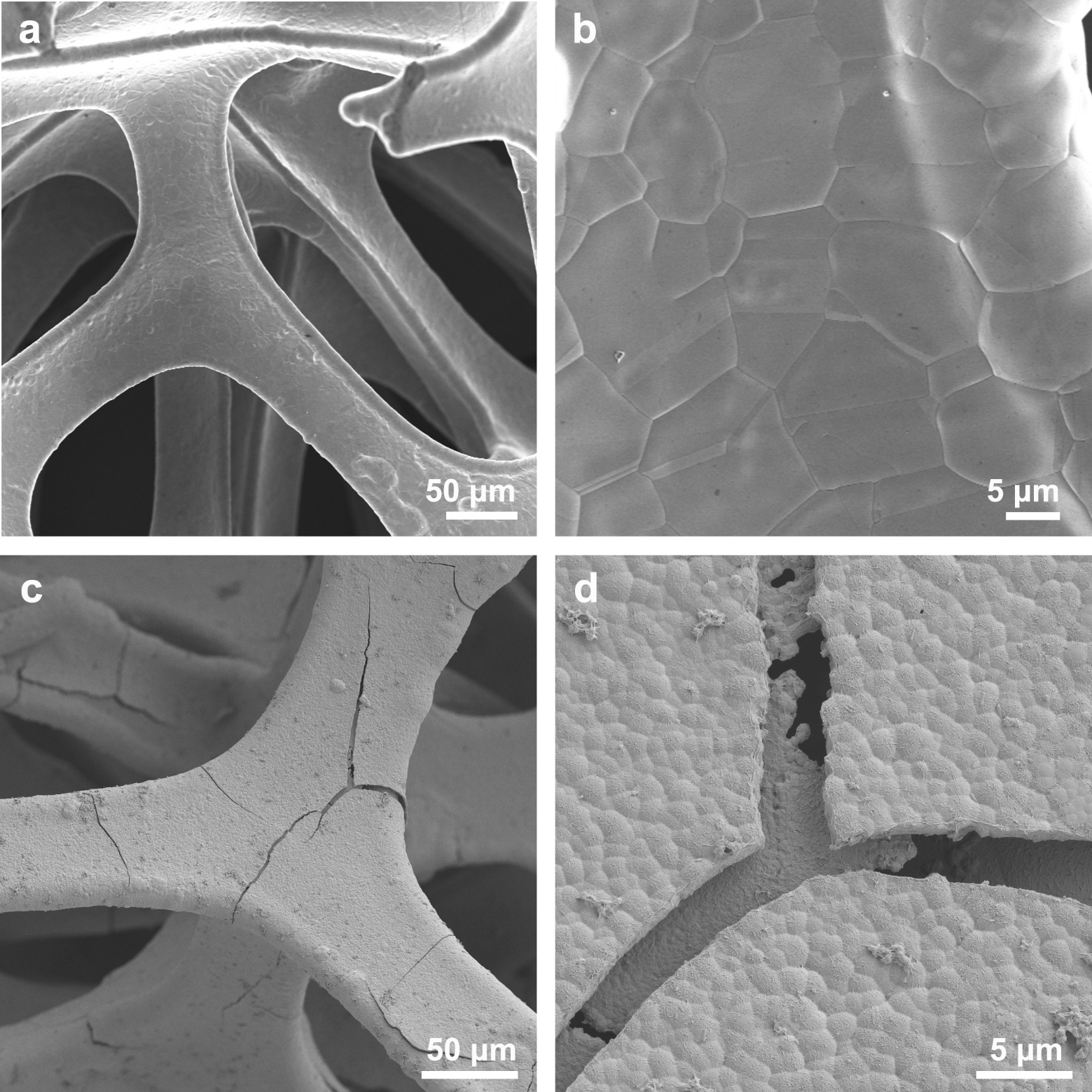


**Figure S7.** SEM images of a, b) Nickel foam, and c,d) Fe-NiWB/NF.


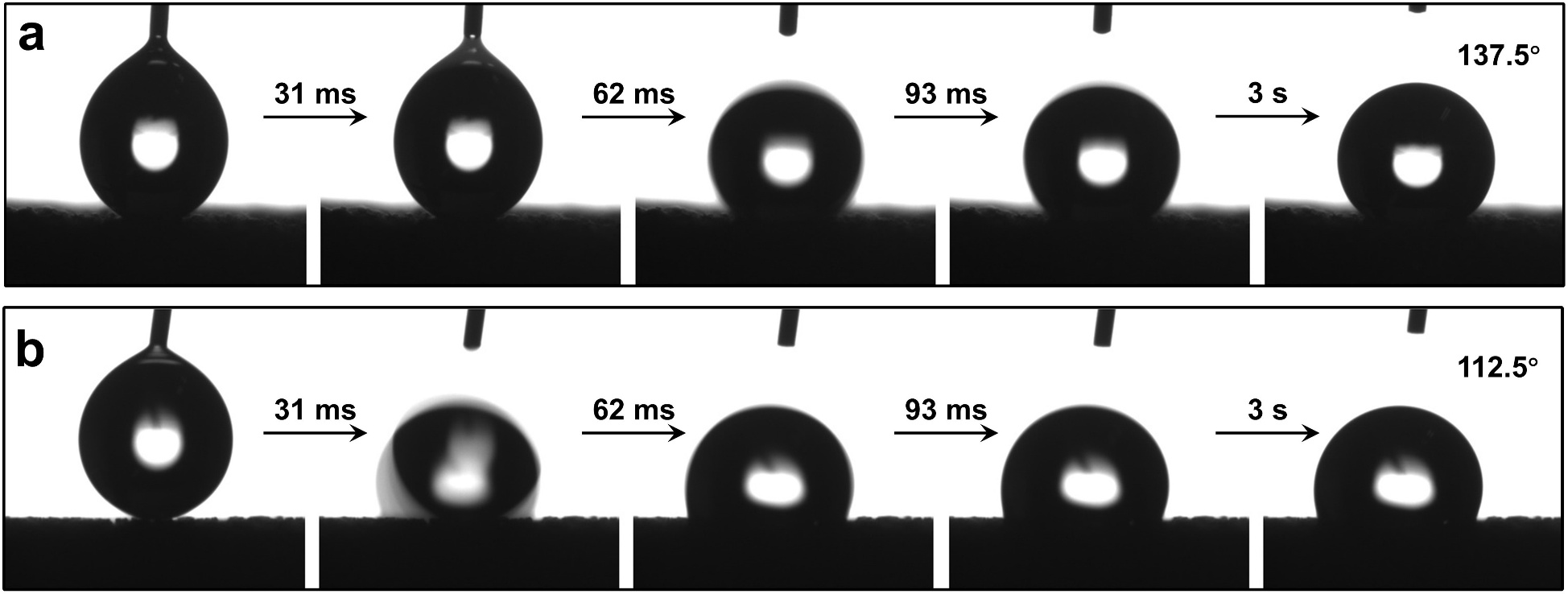


**Figure S8.** Dynamic wetting images of a) Melamine sponge and b) Nickel foam substrate.


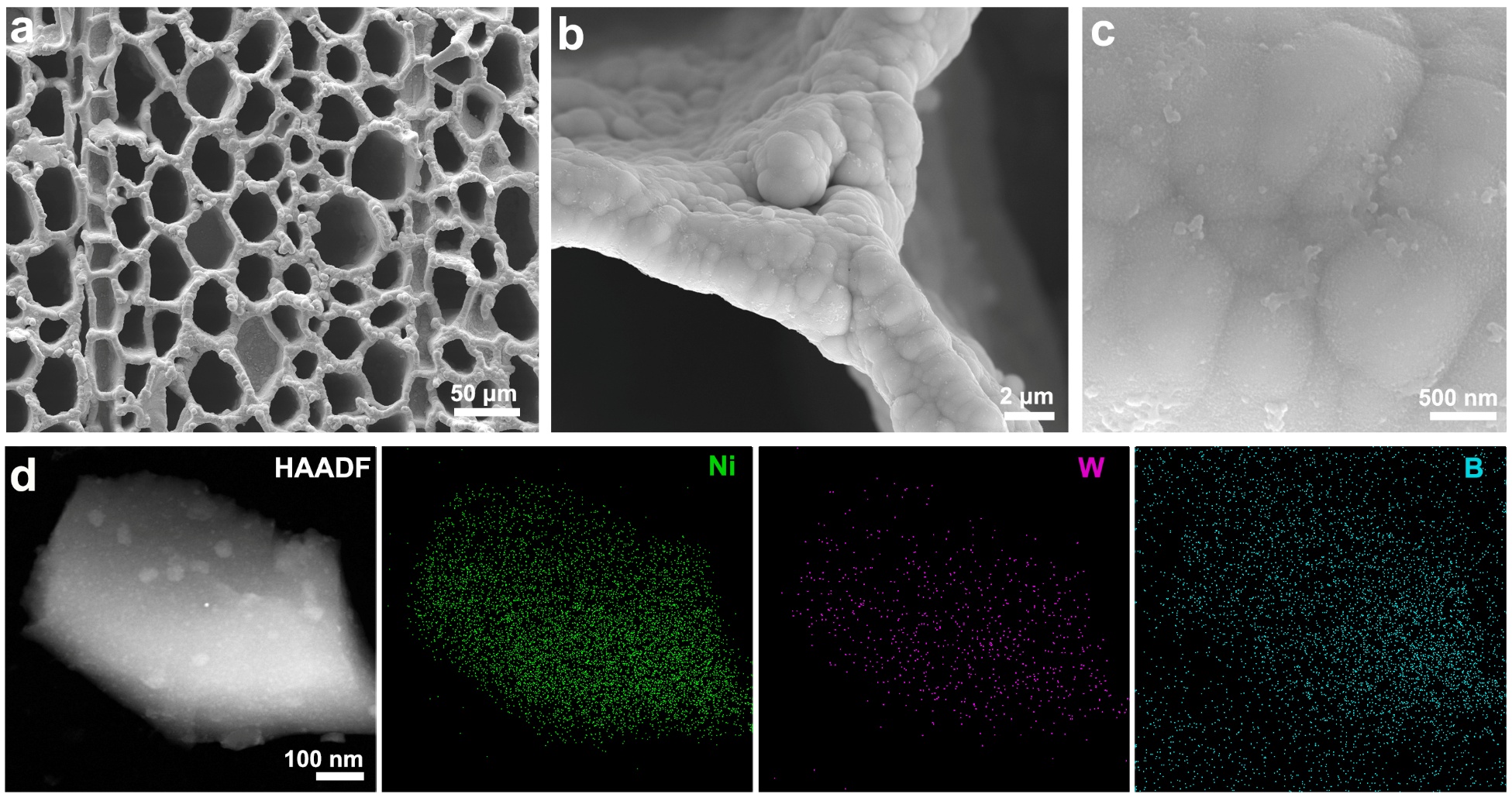


**Figure S9.** a-c) SEM images of the NiWB/PW. d) HAADF-STEM image and corresponding element mapping of Ni, W, and B in the NiWB.


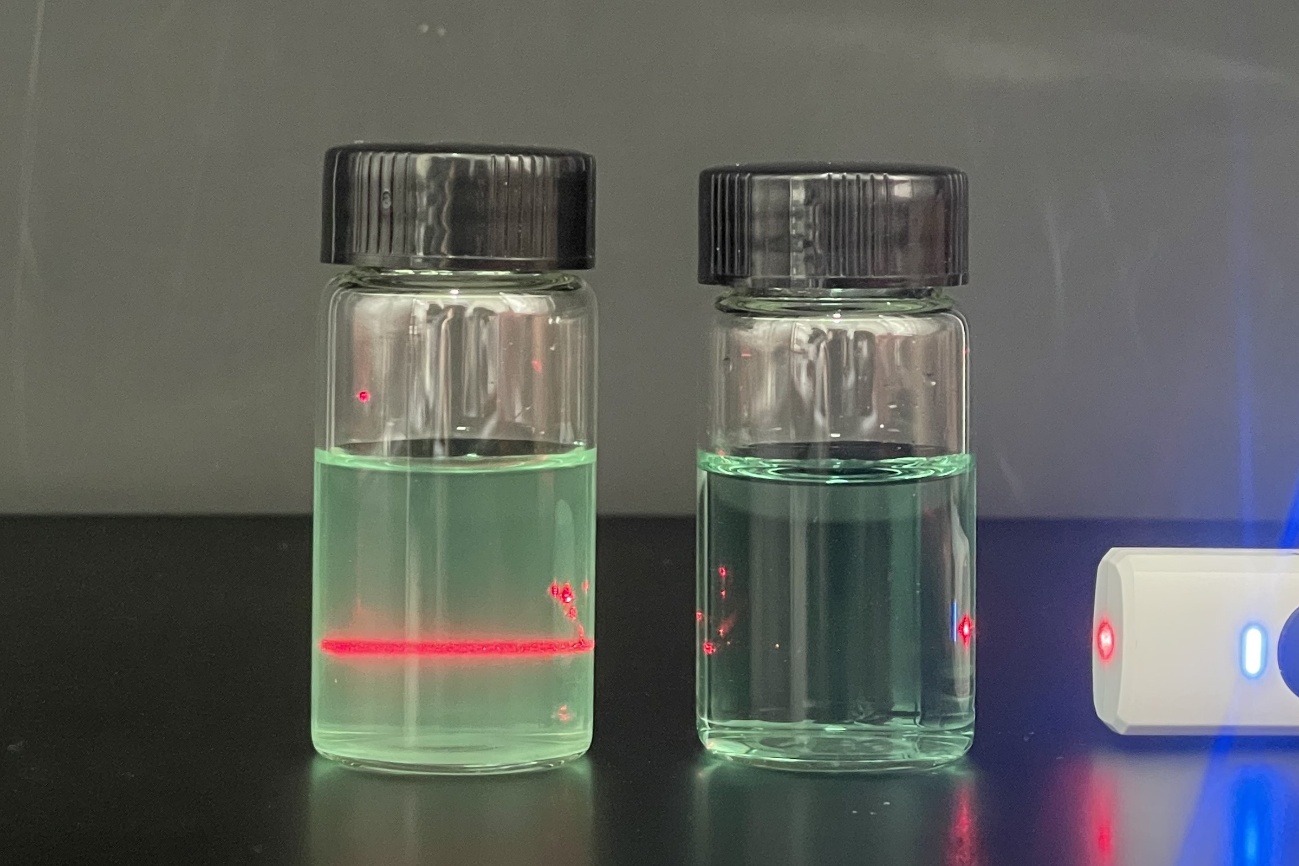


**Figure S10.** Optical images of the as-prepared electroless plating solution of (left) NiWB and (right) Fe-NiWB, and irradiating the solution with a laser pointer incident from the left.

**Note:** In the proposed colloid-mediated electroless plating (CMEP) process, introducing Fe^2+^ ions into the tungstate-containing plating bath immediately turns the initially transparent solution turbid and generates a pronounced Tyndall scattering, indicating the rapid formation of a well-dispersed colloidal suspension (Figure S10).

The plating bath consists of the following components: Nickel sulfate (metal source), dimethylamine borane (reducing agent), sodium succinate (complexing agent), Sodium sulfate (buffer), and Sodium tungstate (stabilizer). Upon addition of ammonium ferrous sulfate, Fe^2+^ ions spontaneously react with tungstate species (WO_4_^2−^) to yield Fe-W-O colloidal particles via partial hydrolysis and condensation. This reaction consumes a fraction of the tungstate stabilizer, subtly modifying the co-deposition kinetics of NiWB. To investigate the nature of this colloidal phase, a reference sample was synthesized under identical conditions without subsequent metal deposition (Figure S11a). Characterization via TEM, XRD, and XPS confirmed the amorphous nature of the product, composed predominantly of Fe^3+^ and W-based oxide species, suggesting the formation of a disordered Fe-W-O phase (Figure S11b-i).

It is important to note that when Fe^2+^ is introduced into aqueous media, it inevitably undergoes self-hydrolysis to form Fe(OH)_3_-type colloids in parallel with the Fe-W-O formation. Thus, to distinguish which colloidal species dominates under CMEP conditions, we additionally examined Fe(OH)_3_ colloids produced by hydrolyzing ferrous ions in the absence of tungstate. Dynamic light scattering (DLS) and ζ-potential analyses (Figure S12) revealed a fundamental difference between the two types of colloids. The Fe-W-O colloids display a hydrodynamic diameter of ≈120 nm and a strongly negative ζ-potential (−42 mV), ensuring excellent colloidal stability. In contrast, Fe(OH)_3_ colloids exhibit a much larger size (~1050 nm) and a weak ζ-potential (−7.4 mV), confirming their poor stability and strong tendency to sediment. These comparisons demonstrate that, under plating conditions, the predominant dispersed species participating in the CMEP process is the Fe-W-O colloid rather than Fe(OH)_3_.

During the CMEP process, the uniformly dispersed Fe-W-O colloids act as dynamic soft templates and kinetic moderators that simultaneously influence the structural and chemical evolution of the growing NiWB film: (1) Physical templating: Their nanoscale size and strong surface charge temporarily adsorb on the growing NiWB surface, creating steric and electrostatic barriers that inhibit dense-layer closure, yielding a porous and interconnected morphology. (2) Chemical modulation: Partial consumption of tungstate reduces homogeneous nucleation and favors site-selective growth, while residual Fe^2+^ becomes incorporated into the amorphous matrix, modifying local coordination and conductivity. (3) Structural outcome: Embedded colloids generate nanoscale voids that evolve into hierarchical porosity (Figure S13), enhancing active-site exposure and mass transport.

With prolonged deposition, the colloid concentration decreases due to sedimentation, leading to a denser and smoother coating. Overall, the Fe-W-O colloids function as dynamic soft templates and chemical moderators, simultaneously governing nucleation, growth, and doping to form the hierarchically porous Fe-NiWB architecture with superior catalytic activity and durability.


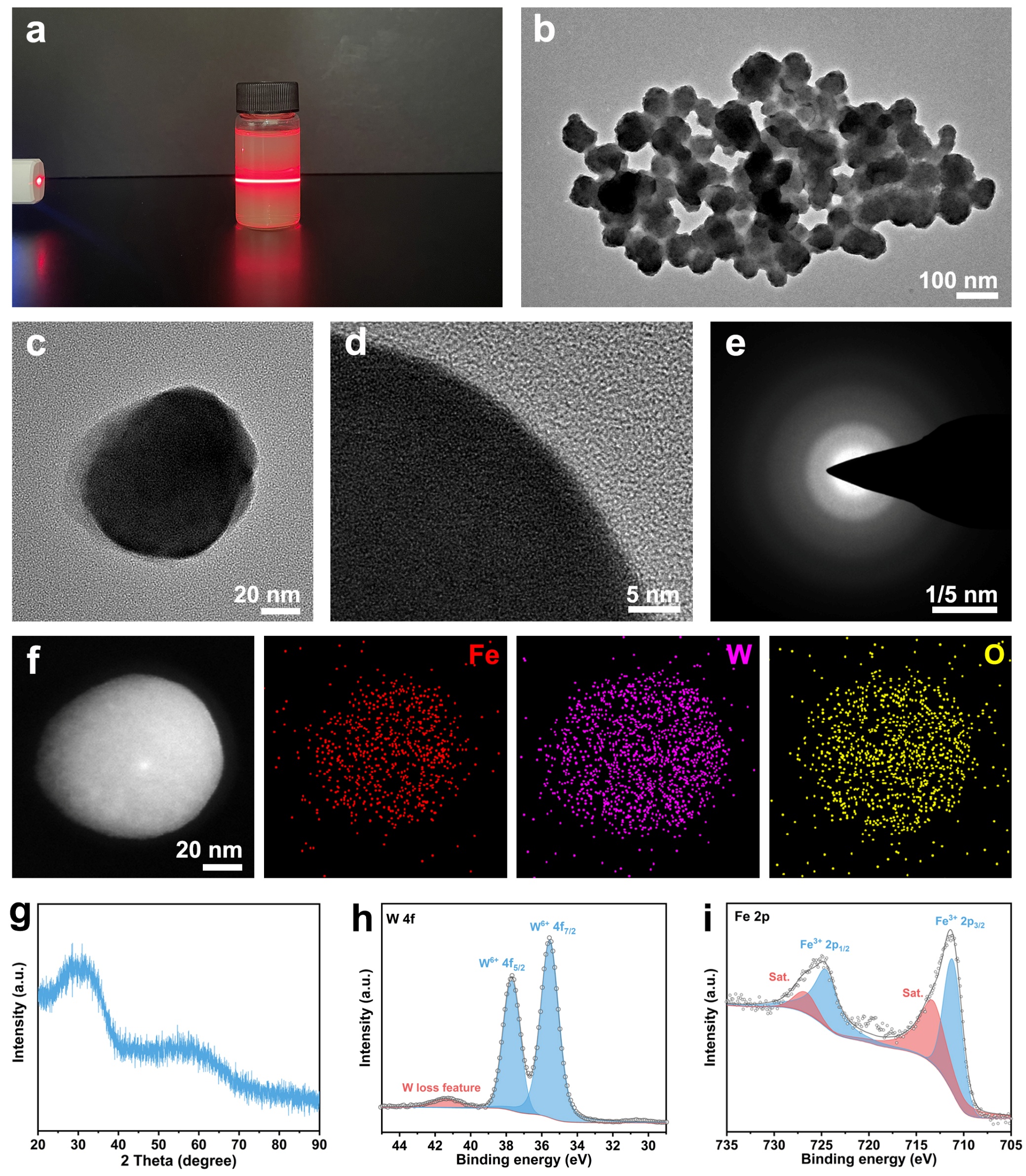


**Figure S11.** Structural characterization of the Fe-W-O colloid. a) Optical images of the colloidal solution. b, c) TEM images, d) HRTEM image, and e) SAED pattern showing amorphous morphology, confirming lack of crystallinity. f) EDS mapping indicating uniform Fe and W distribution. g) XRD pattern showing amorphous structure. h, i) high-resolution XPS spectra of W 4f, and Fe 2p confirming W^6+^ and Fe^3+^ states.


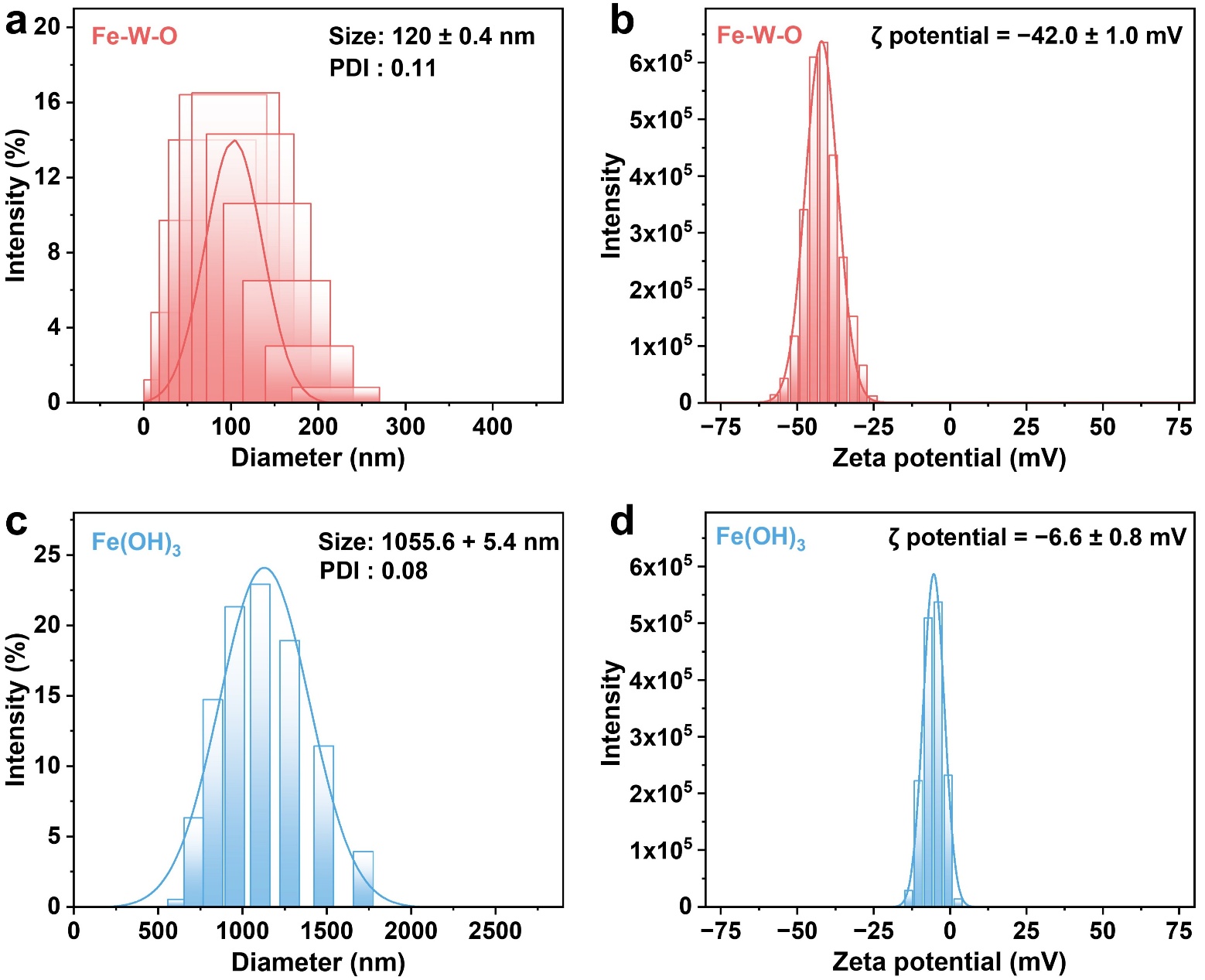


**Figure S12.** Particle size distribution and zeta potential of (a, b) Fe-W-O colloids and (c, d) Fe(OH)_3_ colloids.


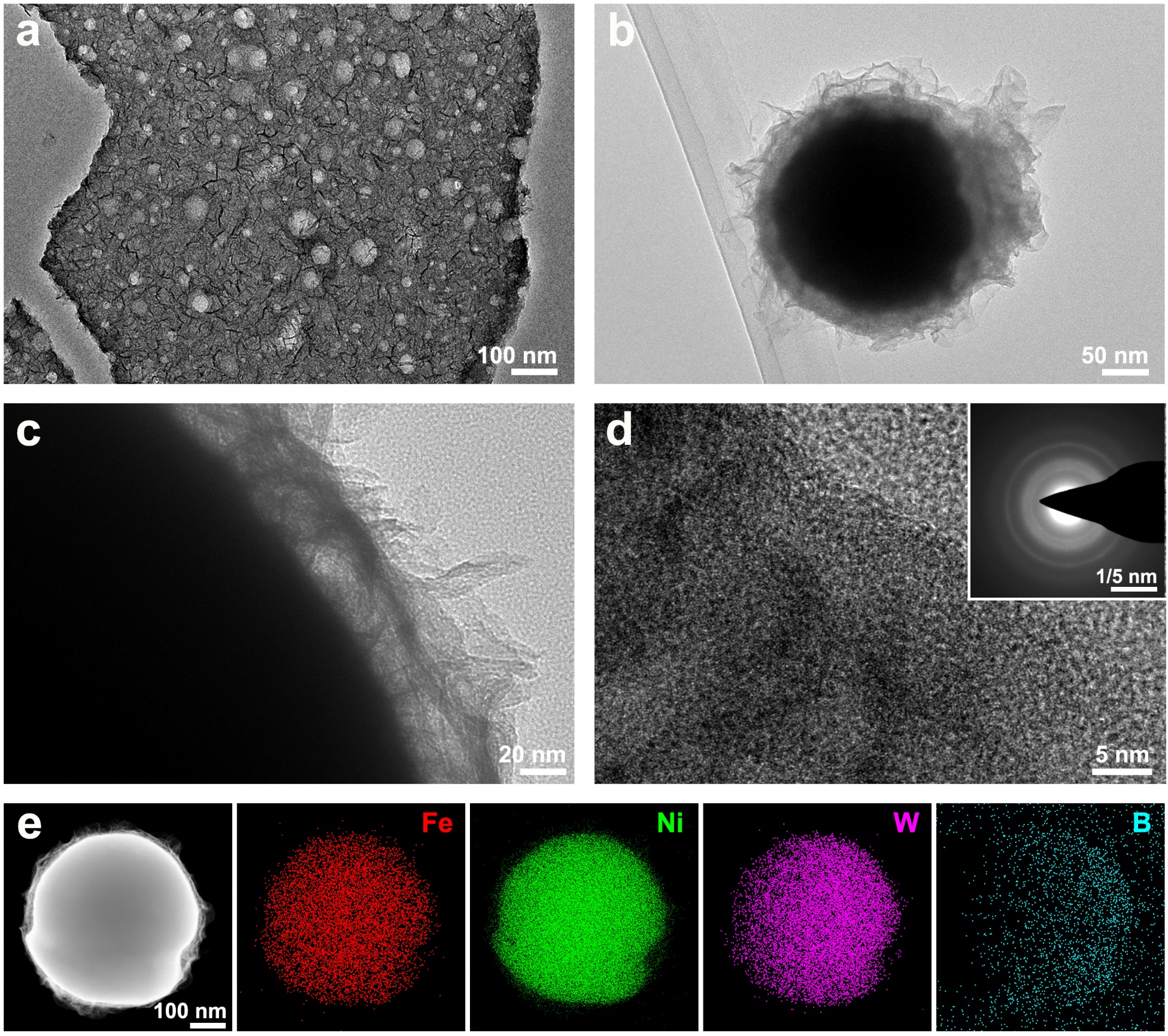


**Figure S13.** a) TEM image of the porous Fe-NiWB layer. b, c) TEM images, d) HRTEM image (inset is SAED pattern), and EDS elemental maps of the Fe-W-O colloidal particles coated with Fe-NiWB layer.


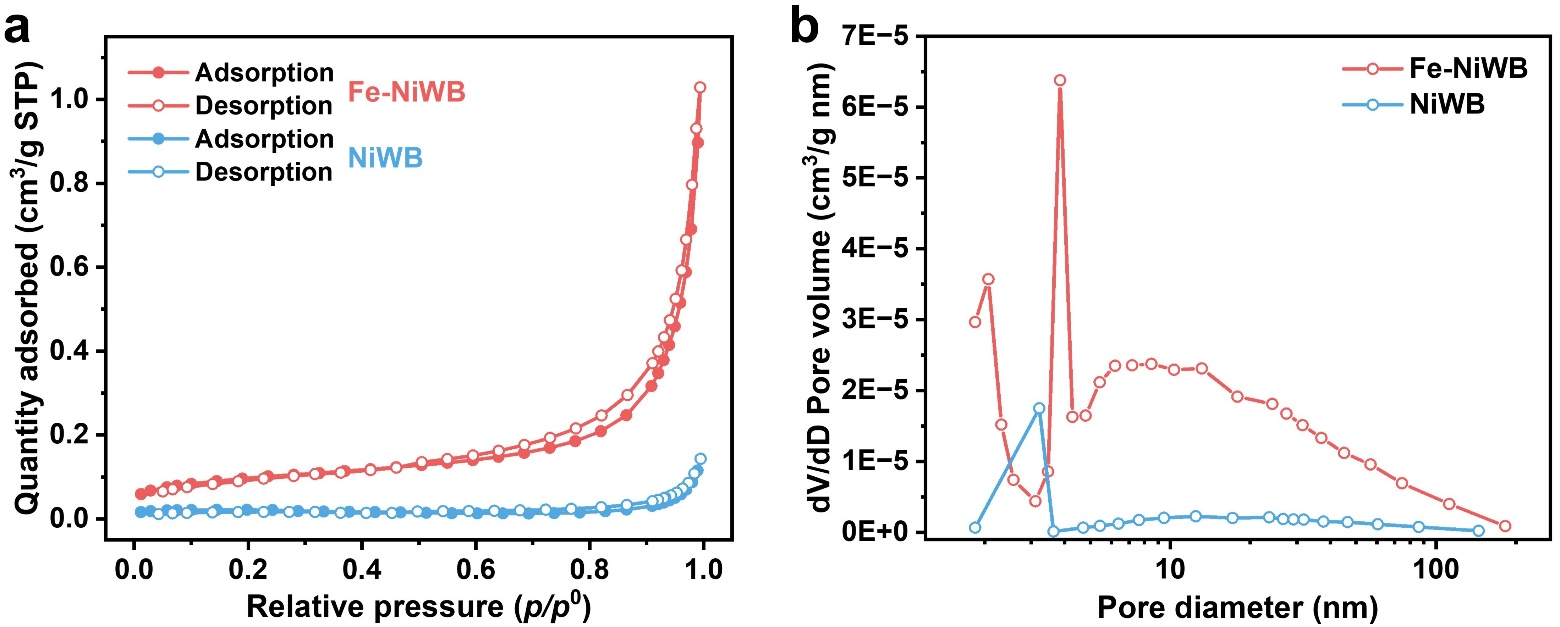


**Figure S14.** a) N_2_ adsorption–desorption isotherms and b) BJH pore-size distributions of NiWB and Fe-NiWB. Minor narrow peaks at ~3–4 nm may arise from model limitations and should be interpreted semi-quantitatively. The overall trend of higher N_2_ uptake and broader pore distribution for Fe-NiWB remains robust.


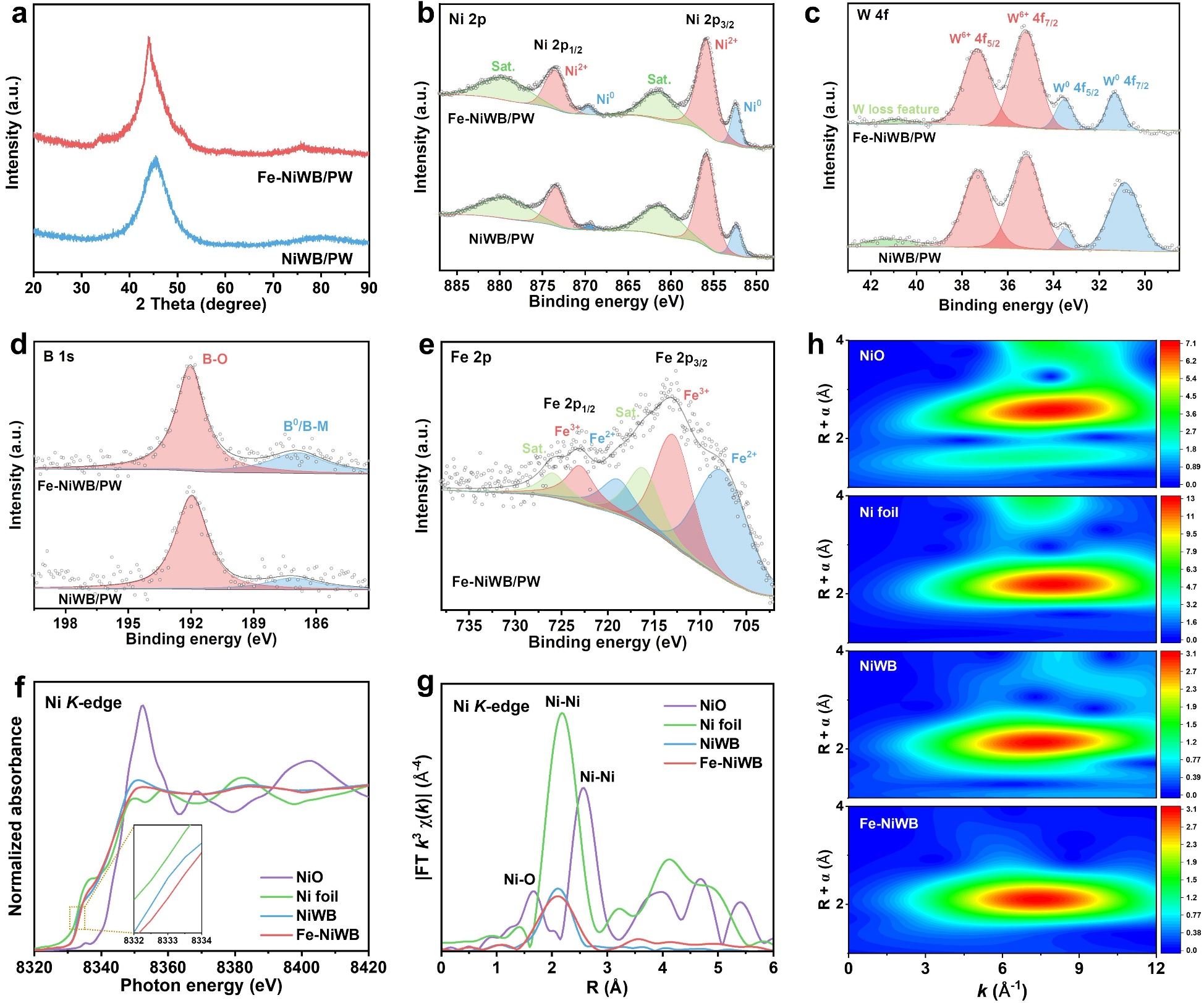


**Figure S15.** Structural characterizations. a) XRD pattern of Fe-NiWB/PW and NiWB/PW. High-resolution XPS spectra of b) Ni 2p, c) W 4f, d) B 1s, and e) Fe 2p of Fe-NiWB/PW and NiWB/PW. f) Normalized Ni K-edge XANES spectra, g) Fourier transforms of Ni K-edge EXAFS, and h Wavelet transform for the *k*^2^-weighted XANES signs of Fe-NiWB, NiWB, Ni foil, and NiO.

**Note:** Although the Fe-NiWB sample exhibits slightly more defined diffraction features than NiWB (Figure S15a), this does not indicate long-range crystallization. Instead, Fe incorporation induces short-range atomic ordering within the amorphous NiWB matrix by promoting heterogeneous nucleation and local Ni-Fe/W coordination. This nanoscale ordering enhances framework rigidity and electron transport while preserving amorphous flexibility, which collectively contributes to the catalyst’s high durability and activity.

**
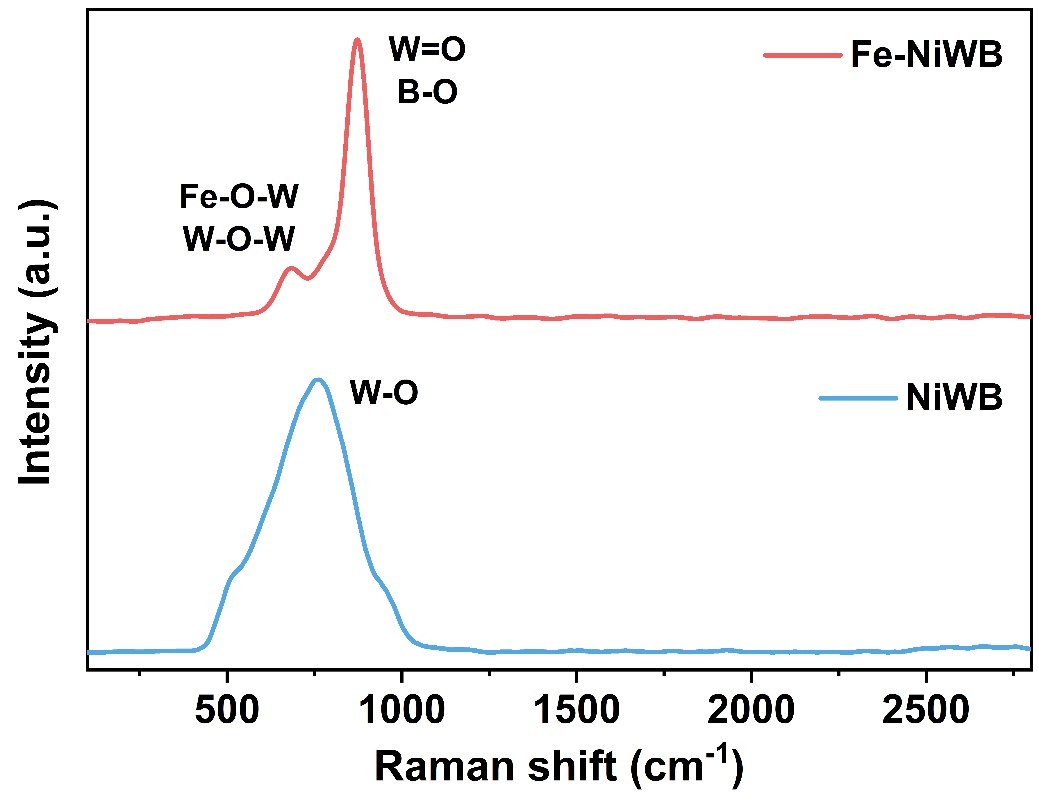
**

**Figure S16**. Raman spectra of NiWB/PW and Fe-NiWB/PW in the dry state, confirming the absence of FeOOH signatures.

**Note:** The NiWB/PW sample exhibits a broad band centered at ~800 cm^−1^, corresponding to the W-O stretching vibration ^[10]^ of an amorphous NiWB framework. In contrast, Fe-NiWB/PW displays a slightly sharper and blue-shifted high-frequency W=O/B-O band (~930 cm^−1^), along with a weak shoulder appearing at ~650–720 cm^−1^, which can be attributed to W-O-W and Fe-O-W vibrations ^[11]^. Notably, no FeOOH-related bands (250–680 cm^−1^) are observed, confirming that Fe species are incorporated into the amorphous NiWB matrix rather than forming crystalline Fe oxyhydroxides on the catalyst surface.


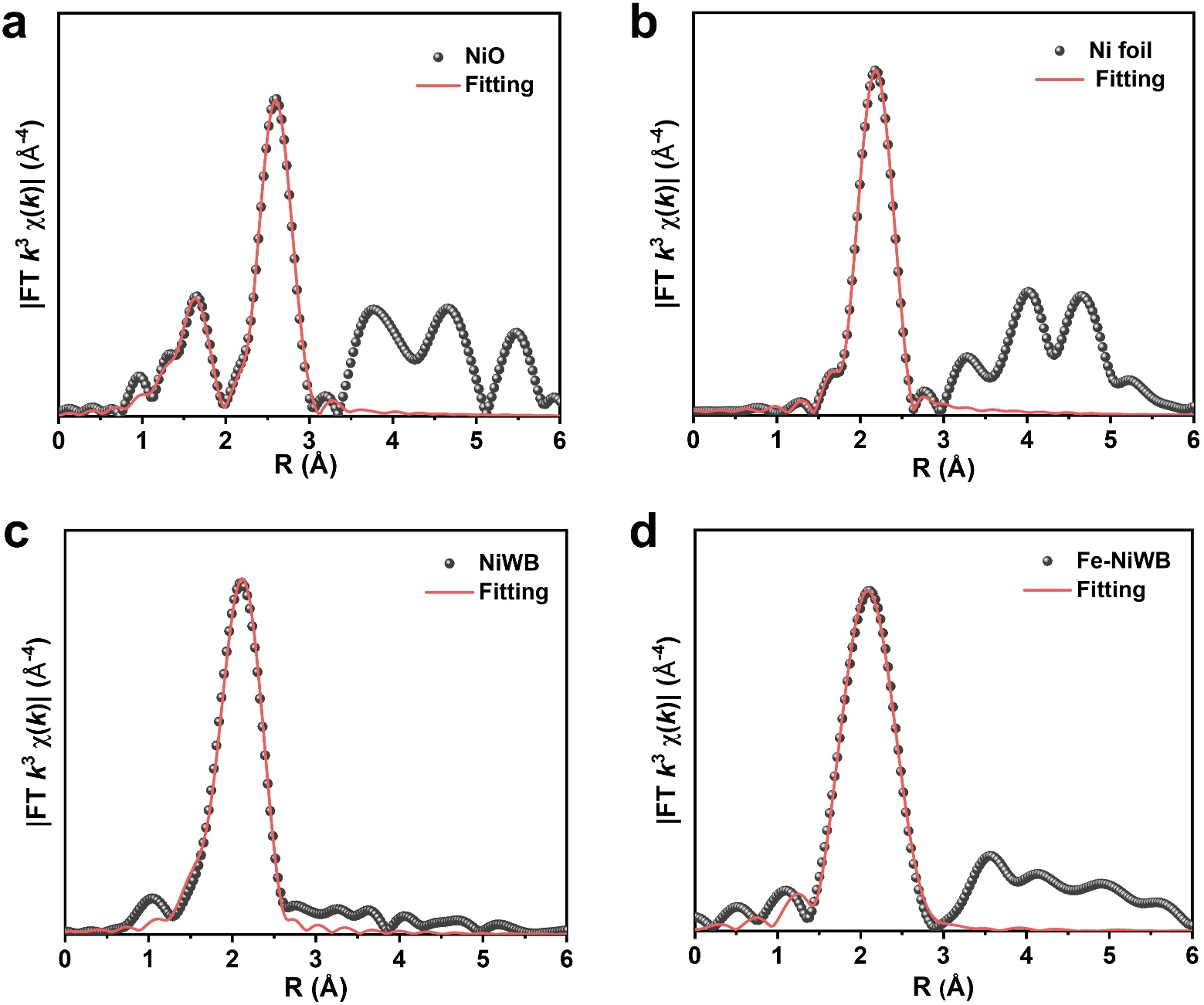


**Figure S17.** The Fourier transformend (FT)-EXAFS spectra in R-space a) NiO, b) Ni foil, c) NiWB, and d) Fe-NiWB.


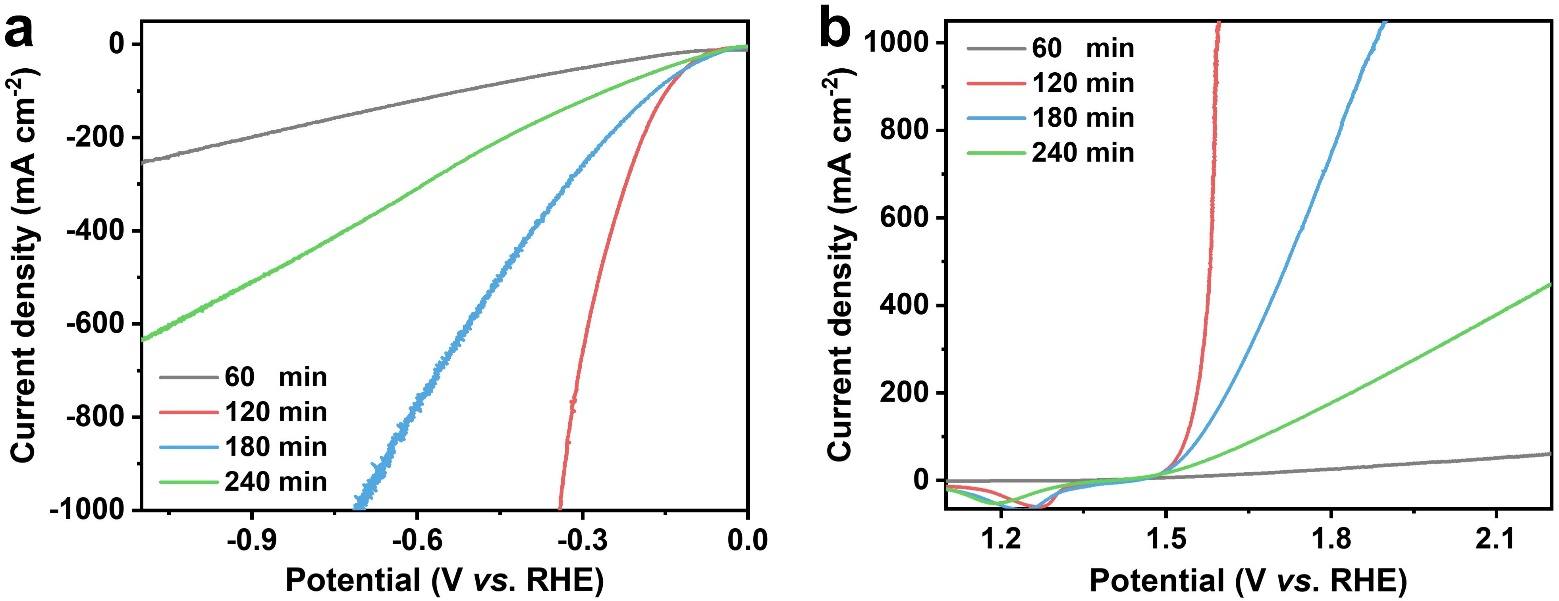


**Figure S18.** LSV curves of Fe-NiWB/PW electrodes with different deposition times for a) HER and b) OER.


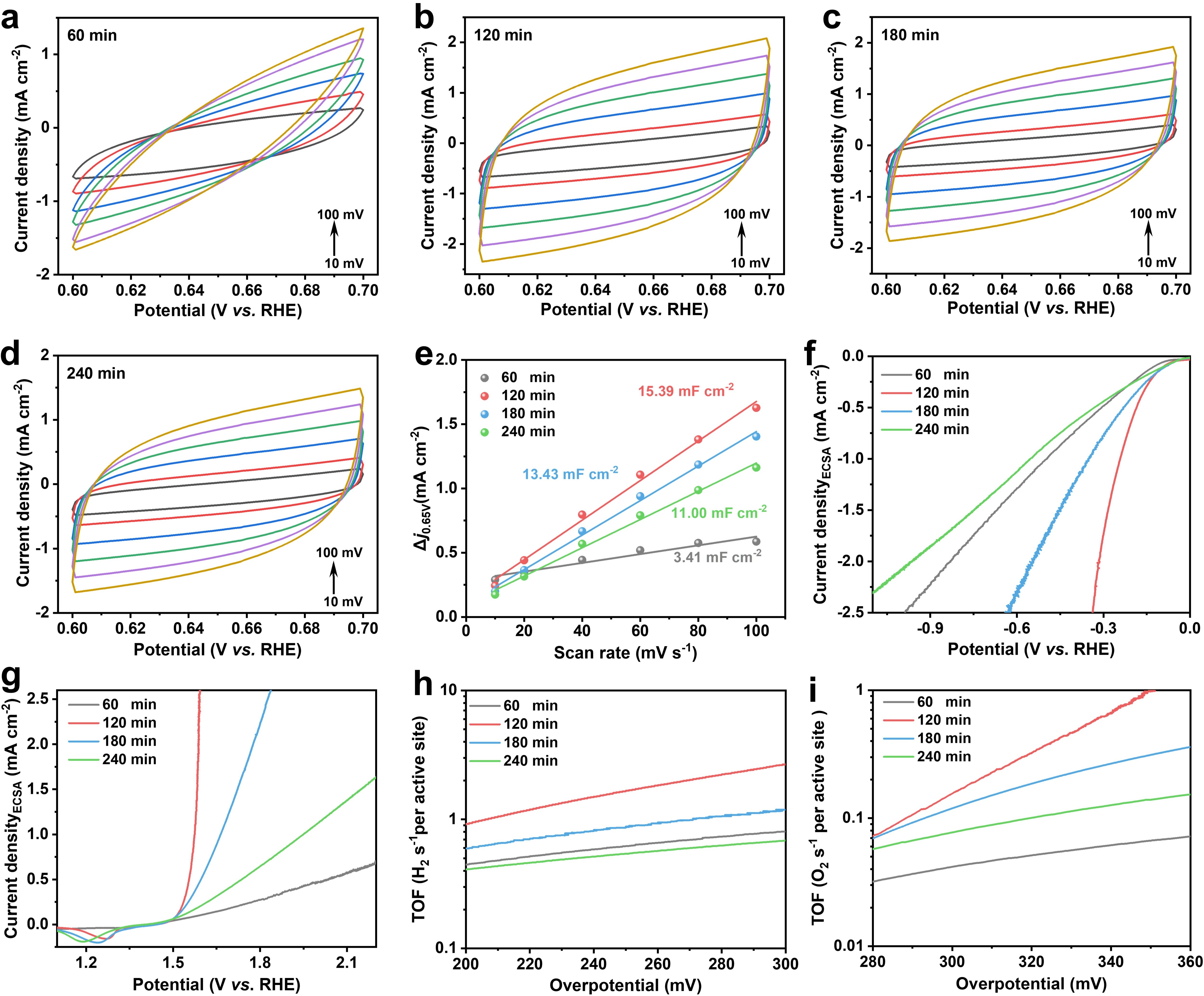


**Figure S19.** CV cluves of Fe-NiWB/PW electrodes with different deposition times (a) 60 min, b) 120 min c) 180 min, and d) 240 min) at diffrent scan rates (10 to 100 mV s^−1^). e) Corresponding *C*_dl_ values (without *iR* correction). ECSA-normalized LSV curves of Fe-NiWB/PW electrodes with different deposition times for a) HER and b) OER.

**Note:** Since ECSA plays a vital role in the assessment of activity, we further investigated its influence. First, the CV curves were recorded in a non-Faradic region (Figure S19a-d). Next, the differences in current density variation$\Delta j=\left| j_{a}-j_{c} \right|/2$ at 0.65 V *vs*. RHE plotted against the scan rate are fitted to estimate the *C*_dl_. The obtained *C*_dl_ values are 3.41, 15.39, 13.43, and 11.00 mF cm^−2^ for Fe-NiWB/PW catalysts deposited for 60, 120, 180 and 240 min, respectively (Figure S19e). The ECSA was calculated according to the following equation, using a conversion factor of 0.04 mF cm^−2^ (typically 0.02 ~ 0.06 mF cm^−2^):

$$A_{ECSA}^{catalyst}= \frac{C_{dl}^{catalyst} mF {cm}^{-2}}{0.04 mF {cm}^{-2} per {cm}_{ECSA}^{2}}$$

Turnover frequency (TOF) values were estimated according to previously reported methods.^[12]^ For the HER, the number of hydrogen turnovers per active site (TOF_H2_) was calculated using the equation:

$$\mathrm{TOF}_{H2}=\frac{{Total hydrogen turnovers}/{\mathrm{cm}^{2} geomric area}}{{Surface sites}/{\mathrm{cm}^{2}} geomric area}$$

The total number of hydogen turnovers per unit current density can be derived as:

$$Number of H_{2}=\frac{j \mathrm{mA}}{\mathrm{cm}^{2}}\times\frac{1 C/s}{1000 mA}\times\frac{1 \mathrm{mol}e^{-}}{96485 C}\times\frac{1 \mathrm{mol}H_{2}}{2 \mathrm{mol}e^{-}}\times\frac{6.02\times{10}^{23} H_{2}}{1 \mathrm{mol}H_{2}}$$

$$=3.12\times{10}^{15}\frac{{H_{2}}/s}{\mathrm{cm}^{2}}\mathrm{per}\frac{\mathrm{mA}}{\mathrm{cm}^{2}}$$

Similarly, the number of oxygen turnovers per site (TOF_O2_) was determined by:

$$\mathrm{TOF}_{O2}=\frac{{Total oxygen turnovers}/{\mathrm{cm}^{2} geomric area}}{{Surface sites}/{\mathrm{cm}^{2}} geomric area}$$

and the total number of oxygen turnovers per current density is calculated as:

$$Number of O_{2}=\frac{j \mathrm{mA}}{\mathrm{cm}^{2}}\times\frac{1 C/s}{1000 mA}\times\frac{1 \mathrm{mol}e^{-}}{96485 C}\times\frac{1 \mathrm{mol}O_{2}}{4 \mathrm{mol}e^{-}}\times\frac{6.02\times{10}^{23} O_{2}}{1 \mathrm{mol}O_{2}}$$

$$=1.56\times{10}^{15}\frac{{O_{2}}/s}{\mathrm{cm}^{2}}\mathrm{per}\frac{\mathrm{mA}}{\mathrm{cm}^{2}}$$

Since it is challenging to experimentally determine the exact number of catalytically active sites, we adopted a commonly used approximation of surface site density (Γ) for metallic catalysts, assuming all samples share a similar active site density. For flat metallic surfaces, Γ is typically $2\times{10}^{15} \mathrm{sites}/{\mathrm{cm}_{ECSA}^{2}}$ ^[13]^. Therefore, the TOF values for HER and OER can be expressed as:

$$\mathrm{TOF}_{H2}=\frac{(3.12\times{10}^{15}\frac{{H_{2}}/s}{\mathrm{cm}^{2}}\mathrm{per}\frac{\mathrm{mA}}{\mathrm{cm}^{2}})\times|j|}{\left( 2\times{10}^{15} \mathrm{sites}/{\mathrm{cm}_{ECSA}^{2}} \right)\times\mathrm{ECSA}}$$

$$\mathrm{TOF}_{O2}=\frac{(1.56\times{10}^{15}\frac{{O_{2}}/s}{\mathrm{cm}^{2}}\mathrm{per}\frac{\mathrm{mA}}{\mathrm{cm}^{2}})\times|j|}{\left( 2\times{10}^{15} \mathrm{sites}/{\mathrm{cm}_{ECSA}^{2}} \right)\times\mathrm{ECSA}}$$

As shown in Figure S19e, the Fe-NiWB/PW-120 min sample exhibits the largest *C*_dl_ (384.75 mF cm^−2^), corresponding to the highest ECSA. This result indicates that a deposition time of 120 min provides the most abundant and accessible active surface sites. Furthermore, as presented in Figure S19f-i and Table S5, the Fe-NiWB/PW-120 min electrode delivers the highest ECSA-normalized current density and turnover frequency (TOF) values for both HER and OER. Specifically, at an overpotential of 300 mV, the TOF values reach 2.67 s^−1^ (HER) and 0.16 s^−1^ (OER), which are significantly higher than those of samples obtained with shorter or longer deposition durations. These results confirm that the Fe-NiWB/PW-120 min electrode possesses the most intrinsically active surface and optimized catalytic kinetics for both hydrogen and oxygen evolution reactions.


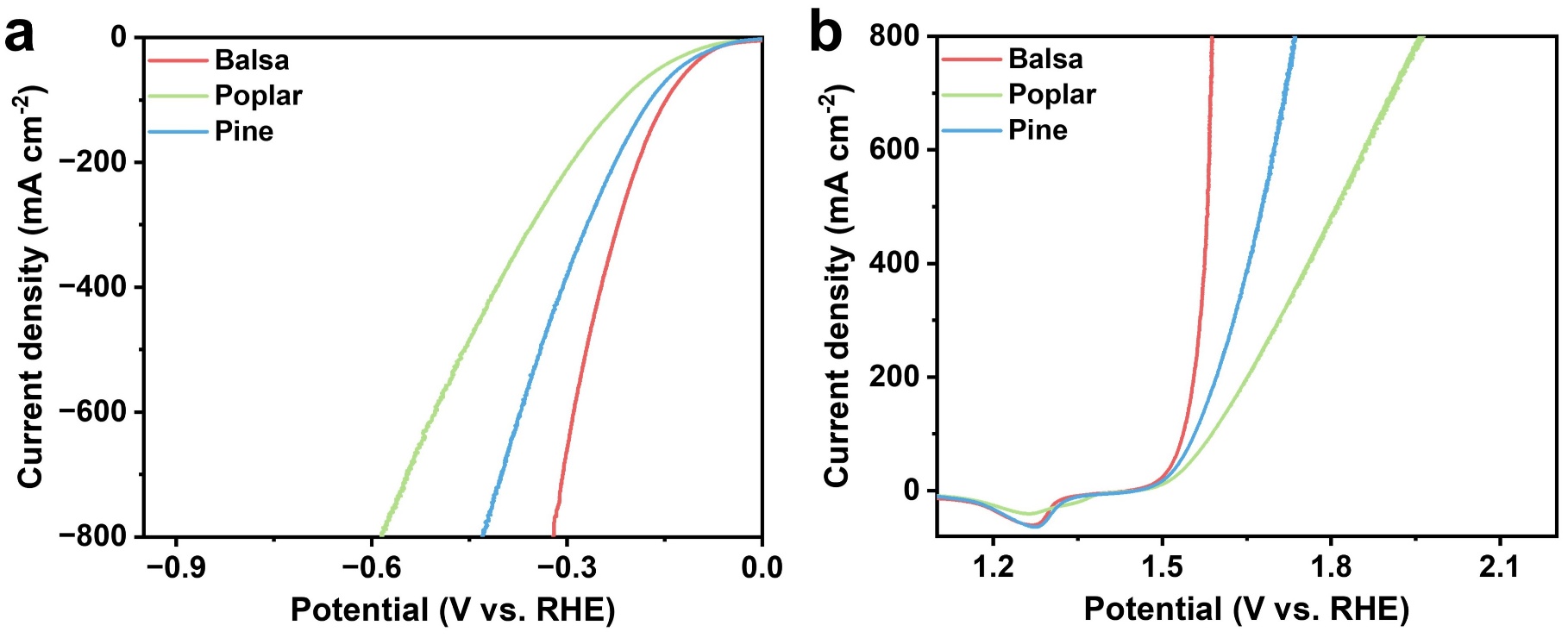


**Figure S20.** a) HER, and b) OER LSV curves of Fe-NiWB catalytic layer deposition on different wood species (balsa wood, poplar wood (*Populus tomentosa* Carr.), and pine wood *(Pinus sylvestris* var. *mongholica* Litv.)).


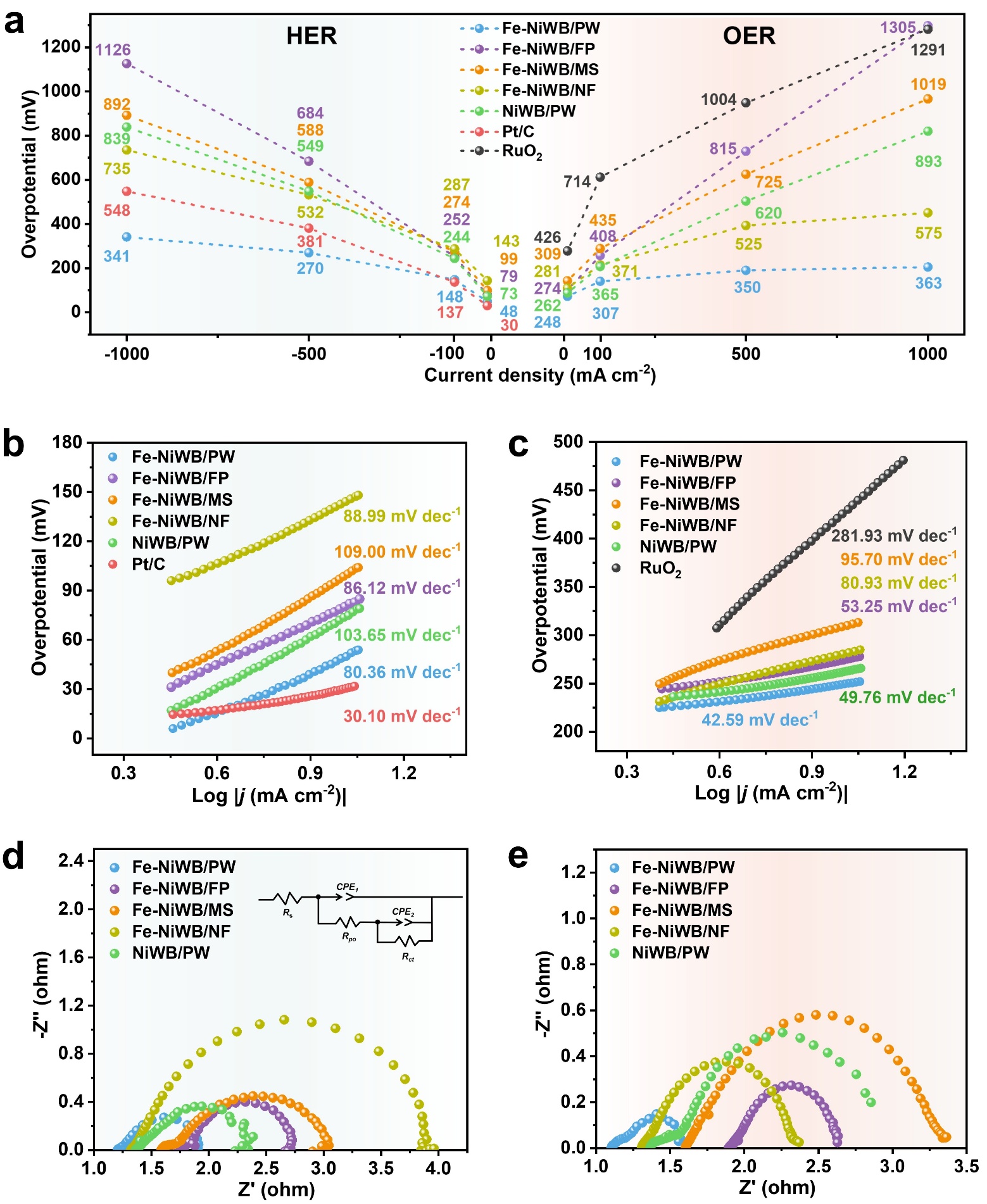


**Figure S21.** a) Overpotentials comparison at 10, 100, 500, and 1000 mA cm^−2^ for HER and OER. Tafel slopes of prepared samples for b) HER and c) OER. Nyquist plot at d) −0.2 V (*vs.* RHE) and e) 1.6 V (*vs.* RHE) of Fe-NiWB/PW and control catalysts (Inset a is the equivalent circuit model for EIS fitting).


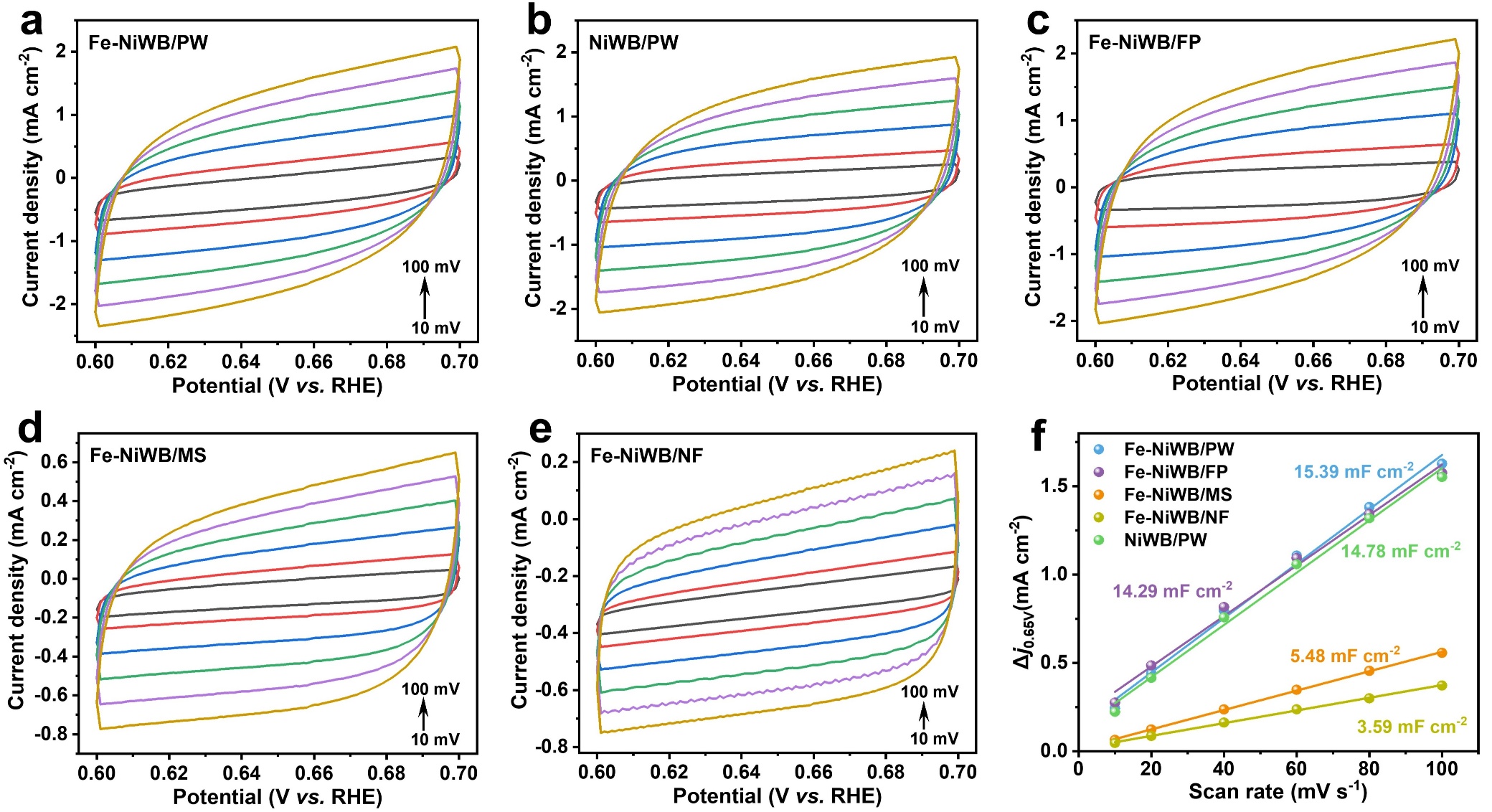


**Figure S22.** CV cluves of a) Fe-NiWB/PW, b) NiWB/PW, c) Fe-NiWB/FP, d) Fe-NiWB/MS and e) Fe-NiWB/NF at diffrent scan rates (10 to 100 mV s^−1^) (without *iR* correction). f) *C*_dl_ values for Fe-NiWB/PW and control catalysts.

**Note:** From the normalized polarization curves (Figure S23), it is evident that the catalytic performance of NiWB/PW is significantly inferior to that of Fe-NiWB/PW, indicating that the incorporation of Fe significantly enhances the intrinsic catalytic activity. In addition, the porous structures of Fe-NiWB/MS and Fe-NiWB/NF exhibit lower intrinsic catalytic activity compared to Fe-NiWB/PW. Interestingly, Fe-NiWB/FP has a comparable active surface area to Fe-NiWB/PW, but exhibits the poorest catalytic performance. This suggests that electrode architecture is a crucial factor influencing electrochemical performance, as it plays a pivotal role in the mass transfer processes associated with electrocatalysis.


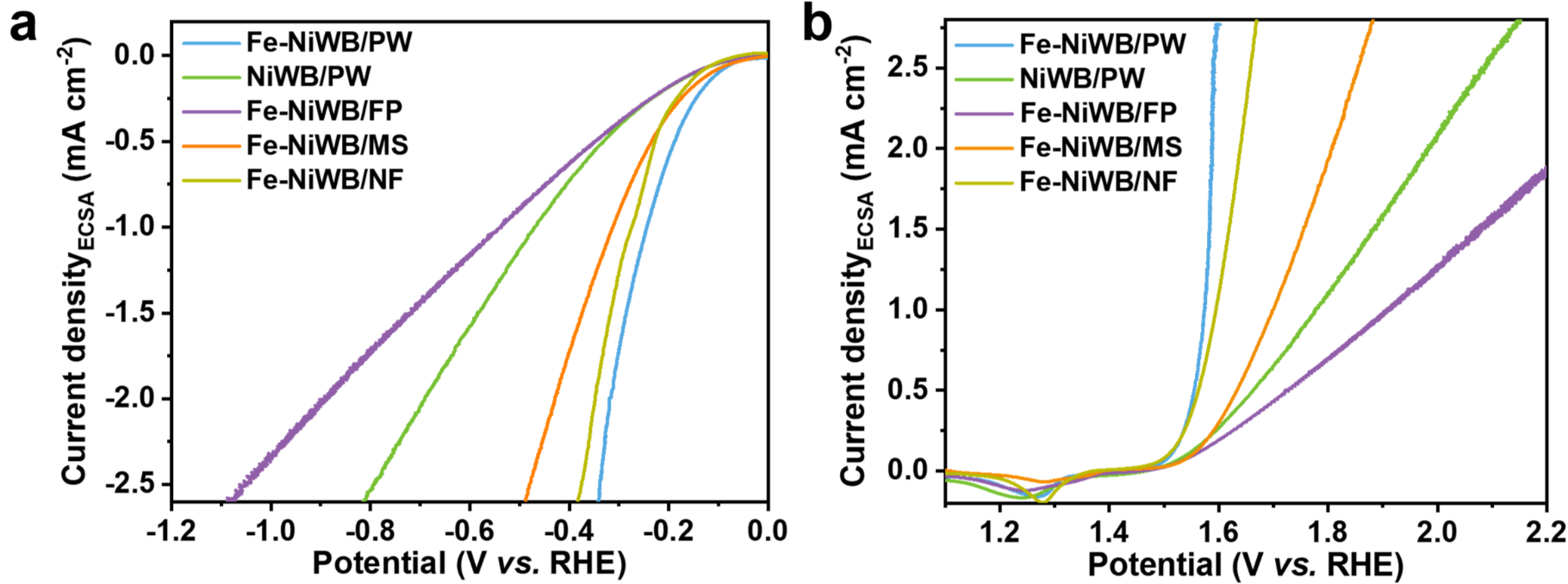


**Figure S23.** a) HER, and b) OER LSV curves of different samples normalized by the ECSA.


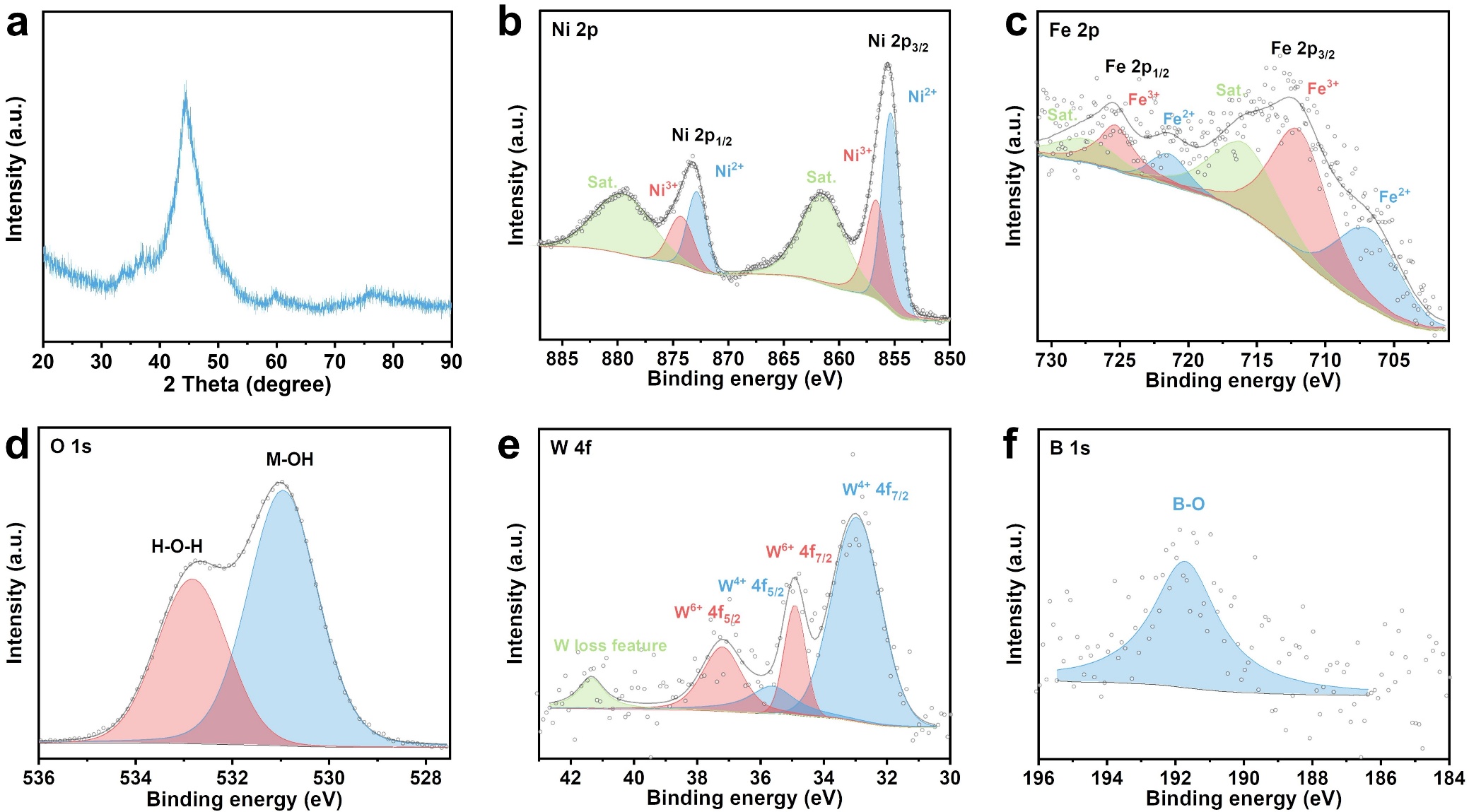


**Figure S24.** a) XRD patterns, b) Ni 2p, c) Fe 2p, d) O 1s, e) W 4f, and f) B1s XPS spectrum of Fe-NiWB/PW electrode 100 h stability test at 500 mA cm^−2^.


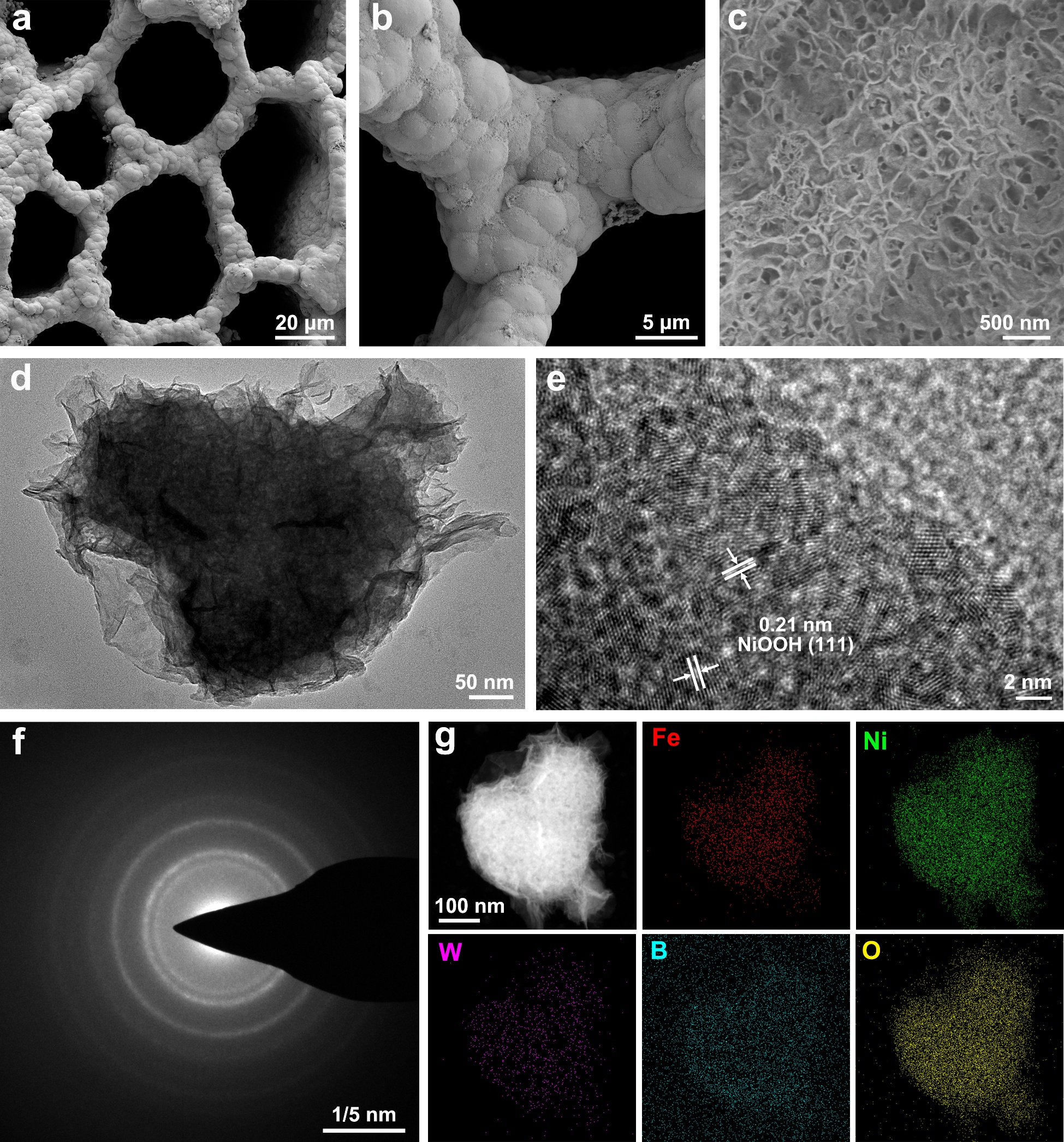


**Figure S25.** a-c) SEM images, d) TEM image, e) HRTEM image, f) SEAD, g) HAADT-STEM image, and EDS mappings of the Fe-NiWB/PW electrode after 100 h stability test at 500 mA cm^−2^.


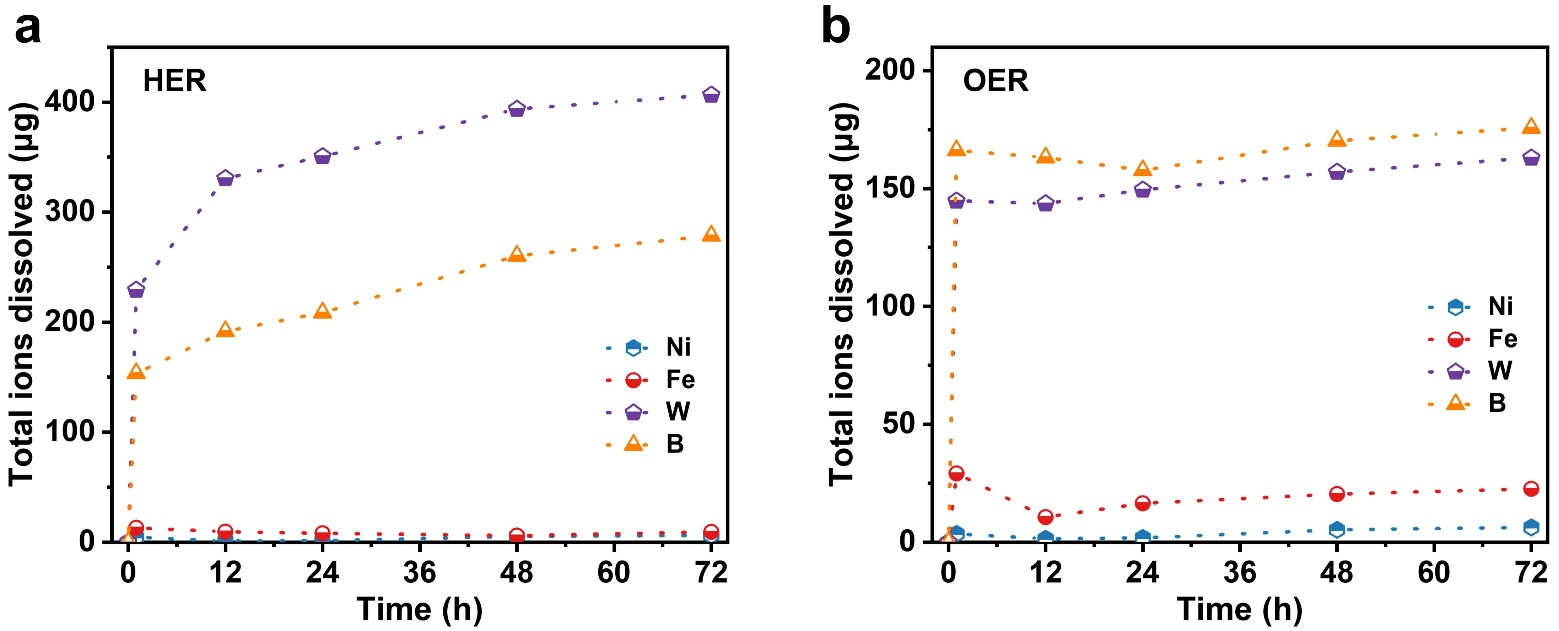


**Figure S26.** Leached ions contents in the electrolyte at different time points during the a) HER, and b) OER stability test of Fe-NiWB/PW. The experiment was performed at a constant current density of −500 and 500 mA cm^−2^.


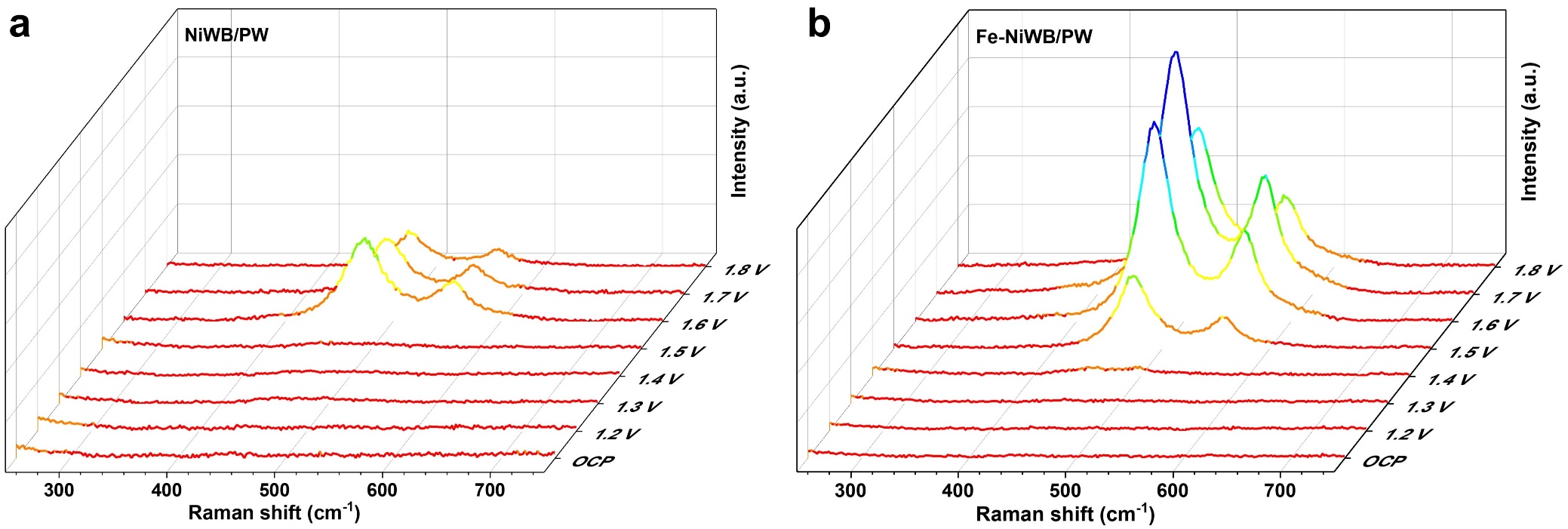


**Figure S27.** In situ Raman spectra of NiWB/PW and Fe-NiWB/PW in the wavenumber region 250–750 cm^−1^ under applied potentials ranging from OCP to 1.8 V vs. RHE.


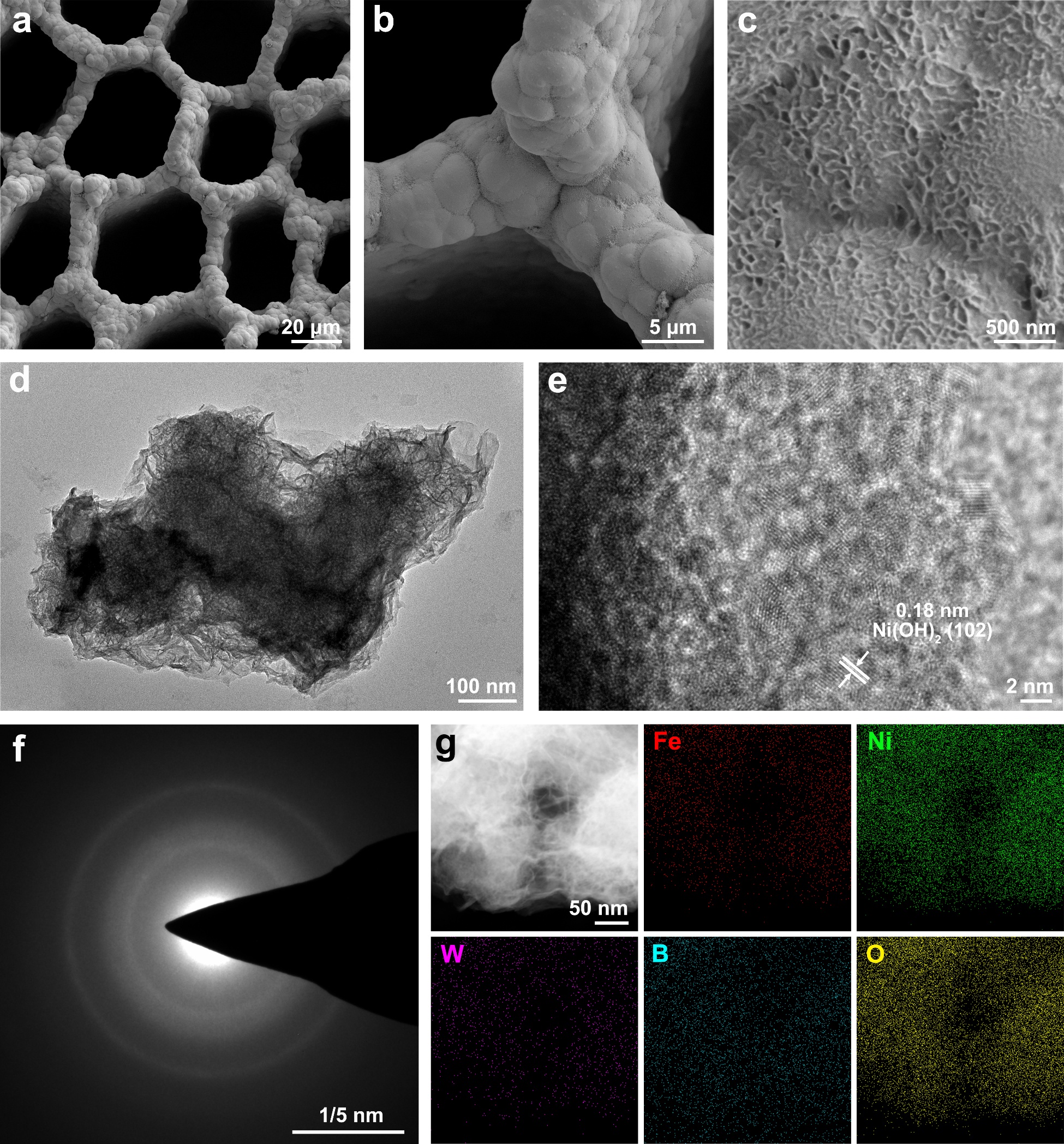


**Figure S28.** a-c) SEM images, d) TEM image, e) HRTEM image, f) SEAD, g) HAADT-STEM image, and EDS mappings of the Fe-NiWB/PW electrode after 100 h stability test at −500 mA cm^−2^.


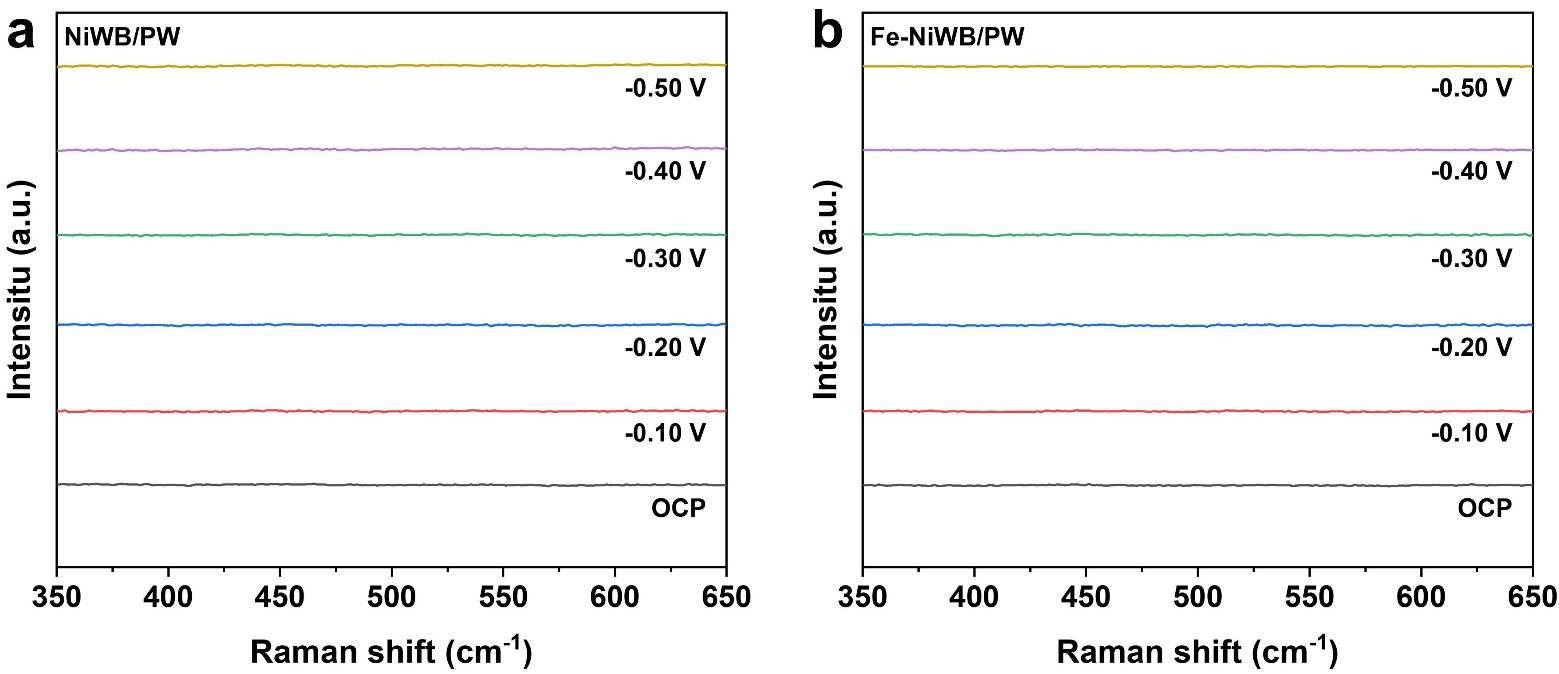


**Figure S29.** In situ Raman spectra of NiWB/PW and Fe-NiWB/PW in the wavenumber region 350–650 cm^−1^ under applied potentials ranging from 0 to −0.50 Vvs. RHE.


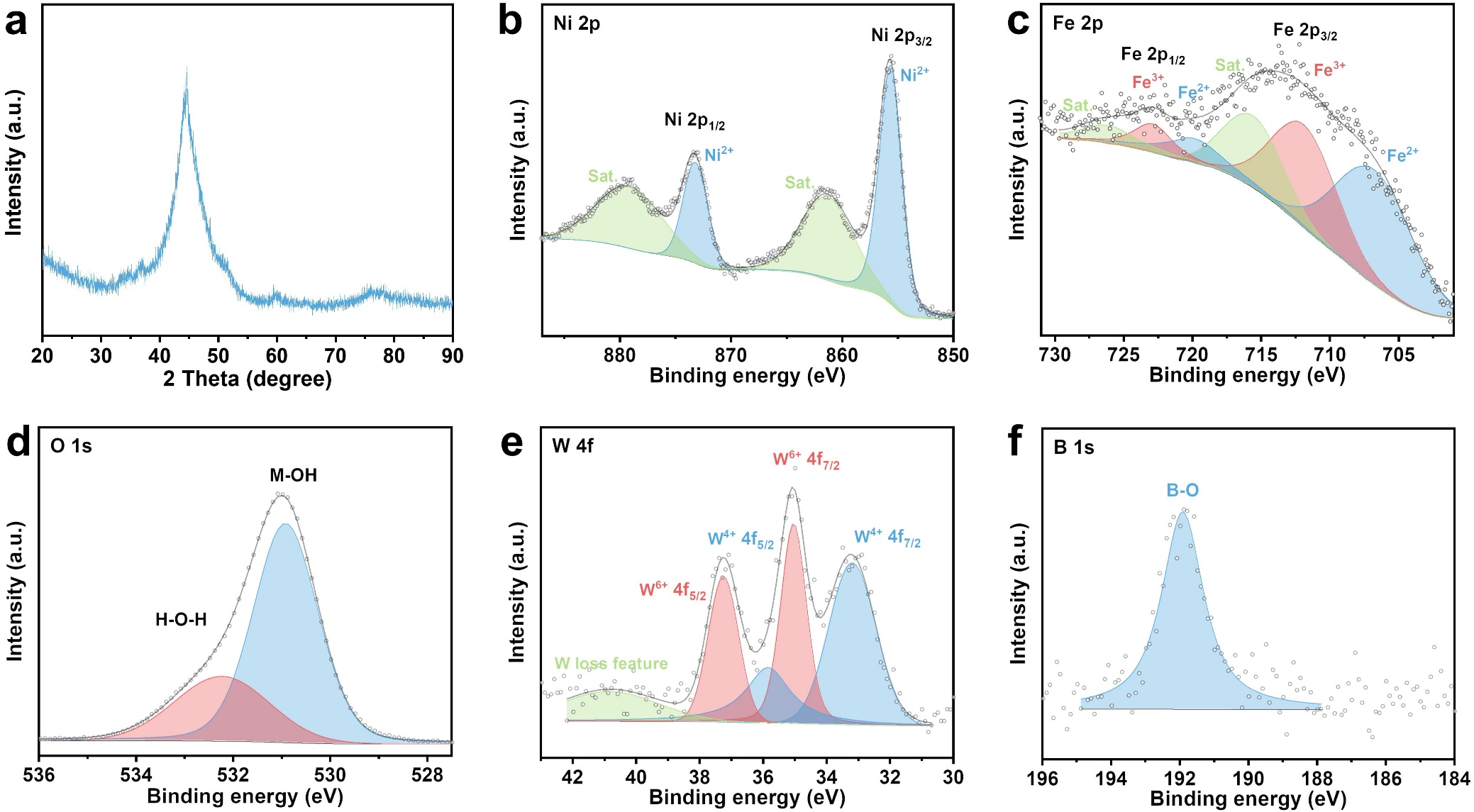


**Figure S30.** a) XRD patterns, b) Ni 2p, c) Fe 2p, d) O 1s, e) W 4f, and f) B1s XPS spectrum of Fe-NiWB/PW electrode after 100 h stability test at −500 mA cm^−2^.


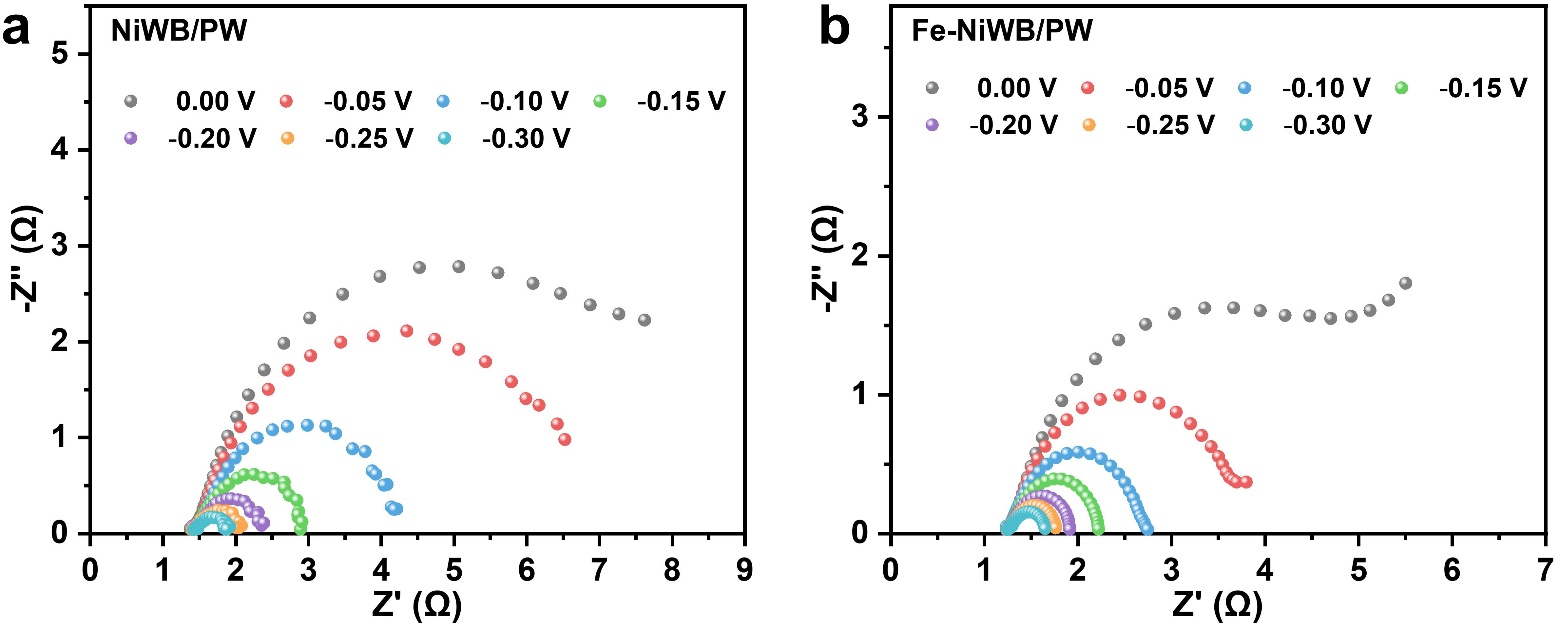


**Figure S31.** Nyquist plots measured at various potentials for the a) NiWB/PW and b) Fe-NiWB/PW during the HER.


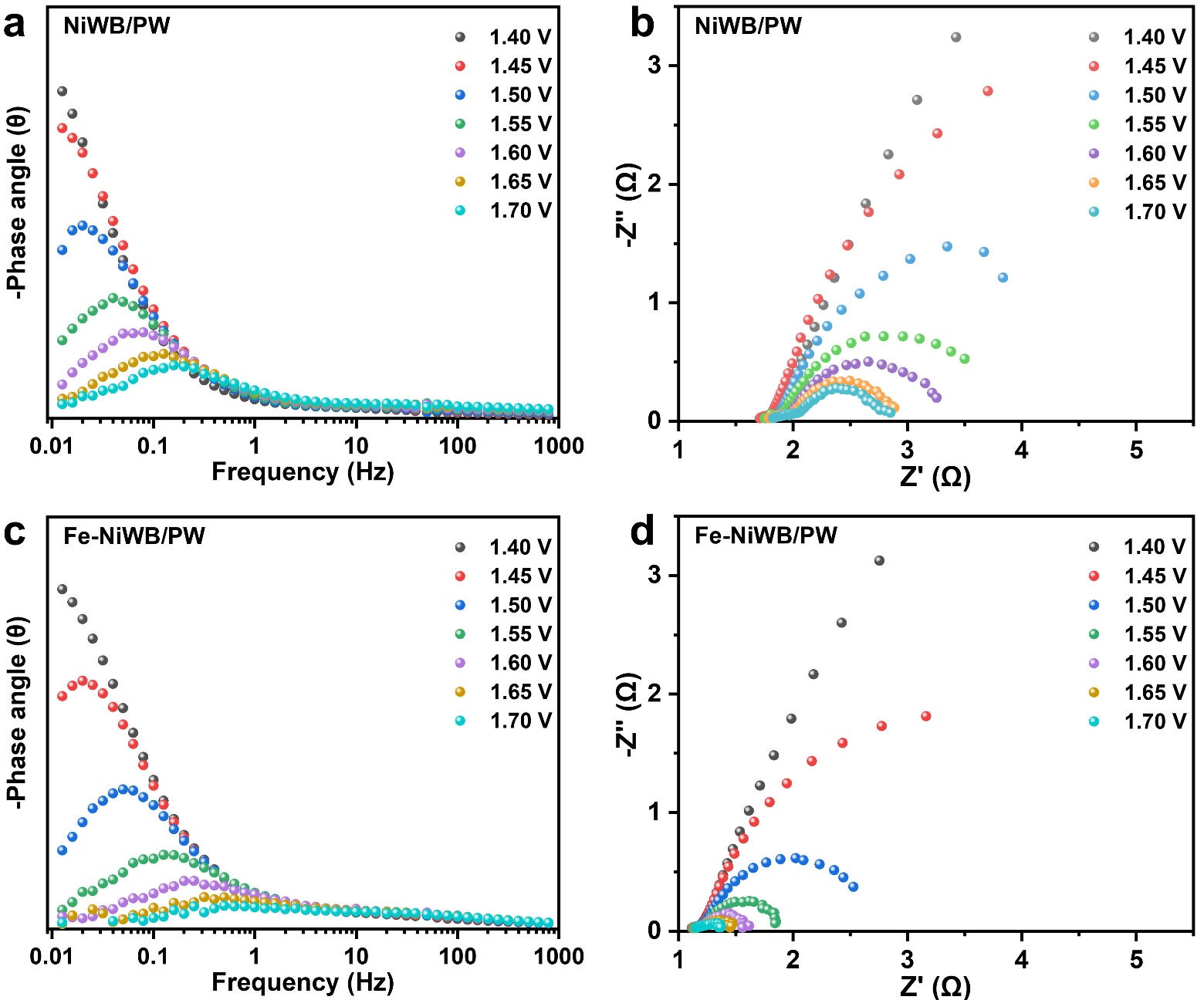


**Figure S32.** Nyquist and Bode plots measured at various potentials for a, b) NiWB/PW and c, d) Fe-NiWB/PW during the OER process.


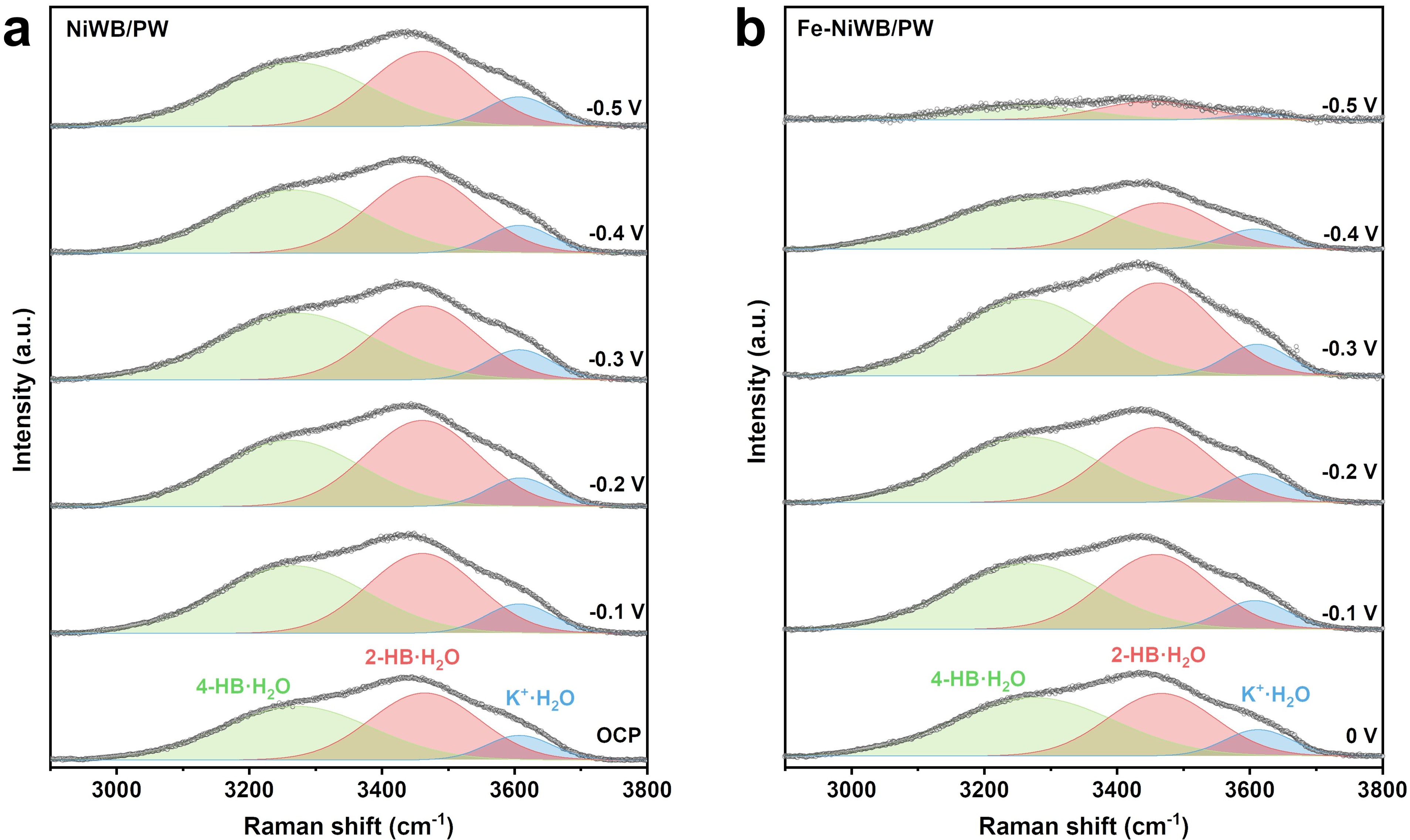


**Figure S33.** In situ Raman spectra for a) NiWB/PW and b) Fe-NiWB/PW under different potentials (V *vs*. RHE), and Gaussian fits of three O-H stretching modes.


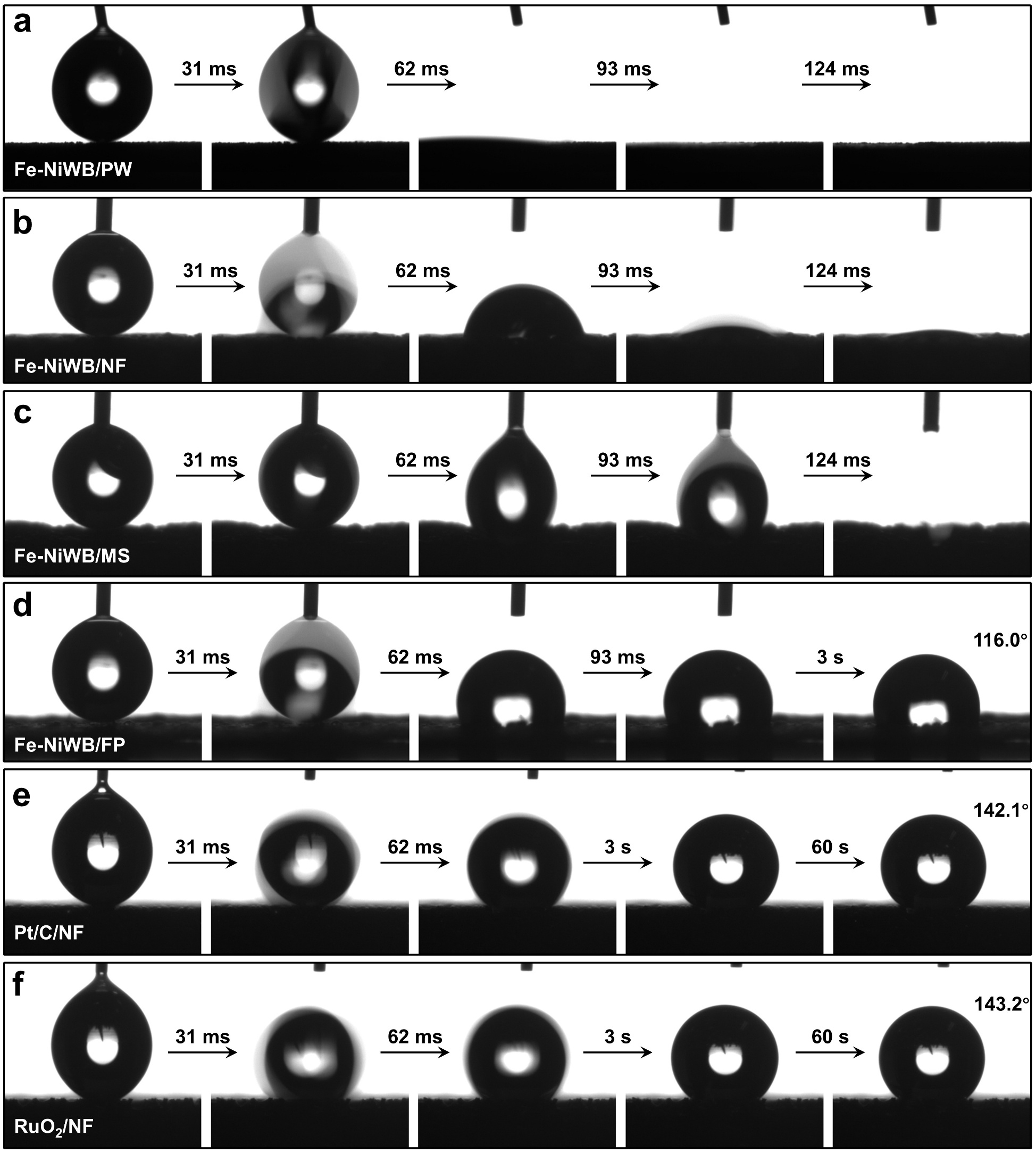


**Figure S34.** The dynamic wettability properties of different samples. Contact angle measurement of 1.0 M KOH drop on a) Fe-NiWB/PW, b) Fe-NiWB/MS, c) Fe-NiWB/NF, d) Fe-NiWB/FP, e) Pt/C/NF, and f) RuO_2_/NF.


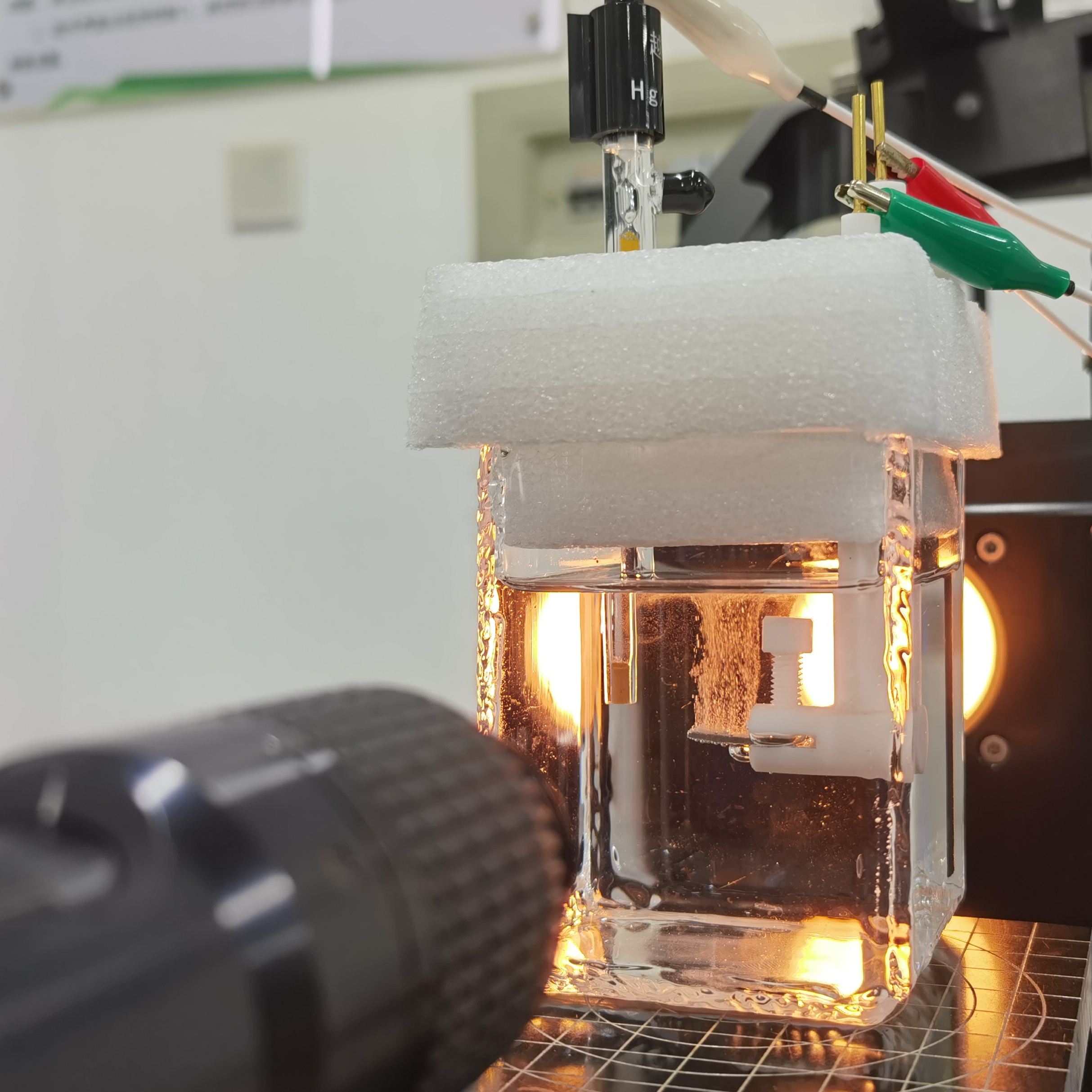


**Figure S35.** A photograph of the experiment setup for observing bubble release behavior during electrocatalytic water splitting.


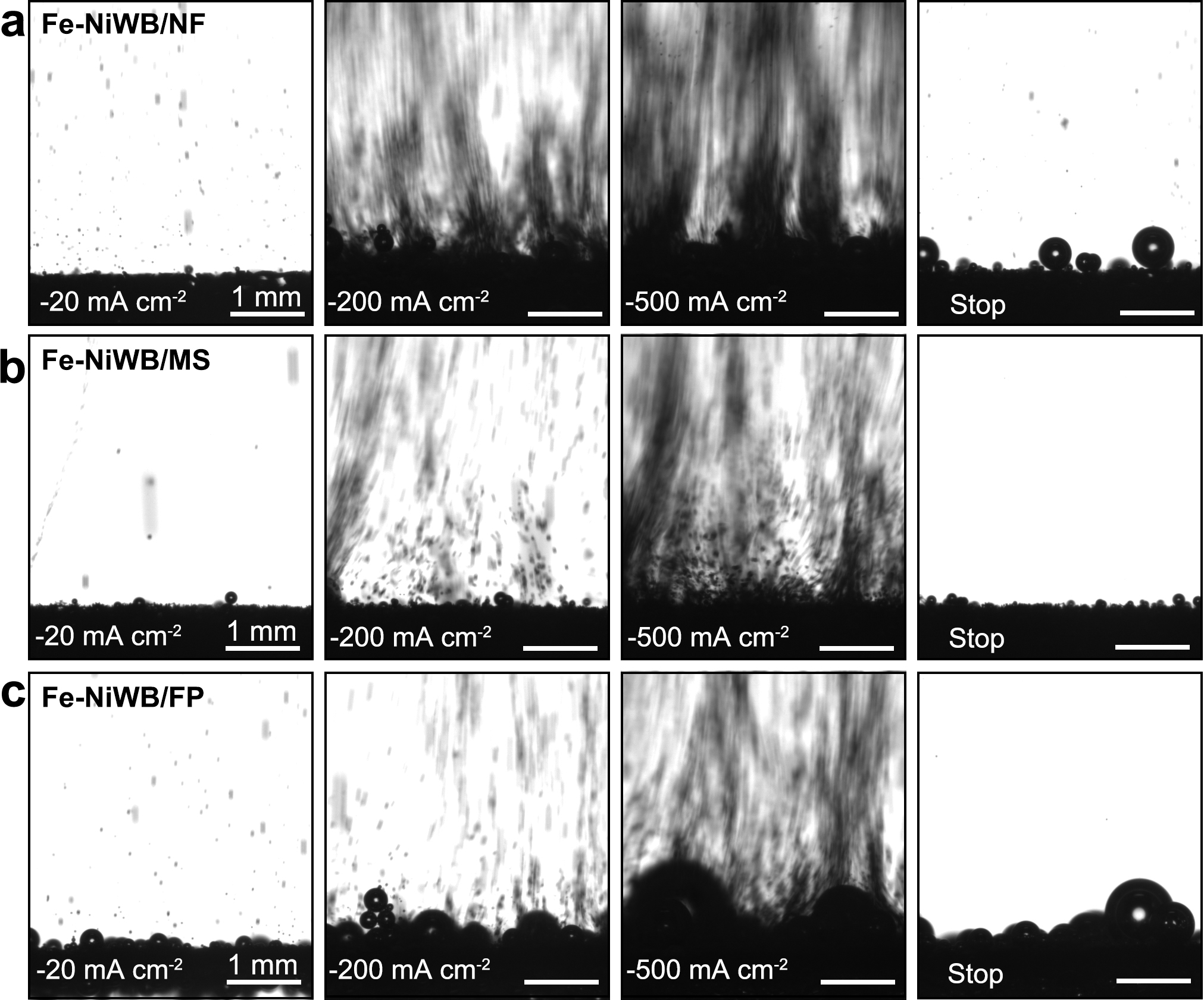


**Figure S36.** High-speed camera images of hydrogen bubbles release from a) Fe-NiWB/NF, b) Fe-NiWB/MS, and c) Fe-NiWB/FP under different current densities.


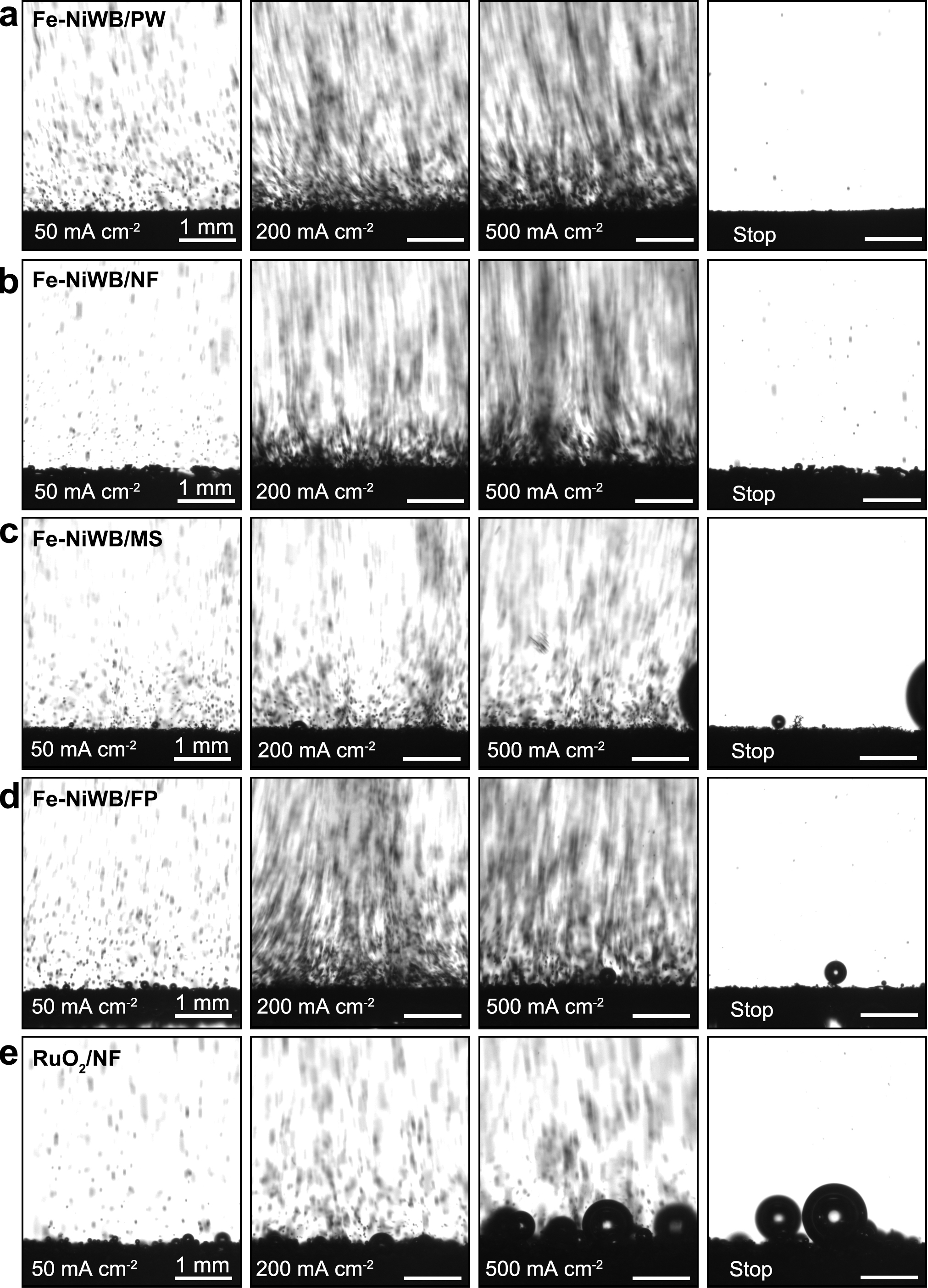


**Figure S37.** High-speed camera images of oxygen bubbles release from a) Fe-NiWB/PW, b) Fe-NiWB/NF, c) Fe-NiWB/MS, d) Fe-NiWB/FP, and e) PtC/NF under different current densities.


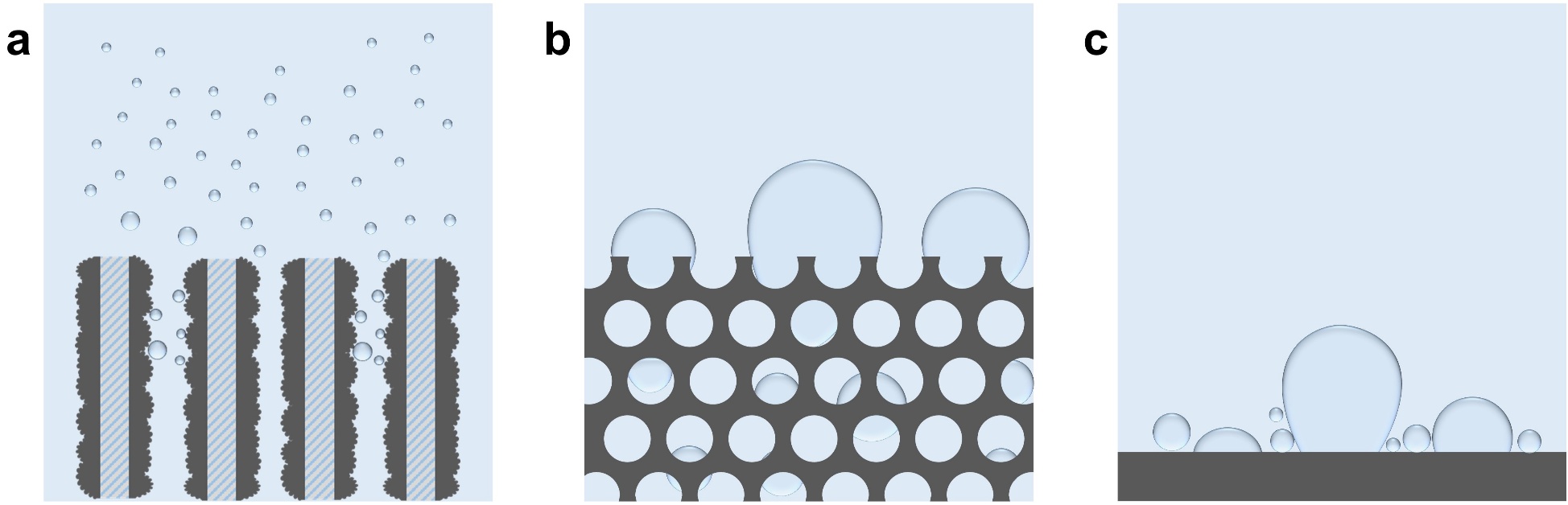


**Figure S38.** Schematic illustration for gas bubble evolution mechanisms on the a) Fe-NiWB/PW, b) Fe-NiWB/NF and Fe-NiWB/MS, and c) Fe-NiWB/FP electrode surface.

**Note:** During water electrolysis, gas bubbles initially nucleate and grow on the electrode surface before detaching upon reaching a critical size. These bubbles impact cell efficiency in two primary ways: adhered bubbles reduce the available electroactive surface area, whereas detached bubbles decrease electrolyte conductivity. In electrodes such as NF, MS, and FP, inefficient bubble detachment properties necessitate the coalescence of numerous smaller bubbles into larger ones before they can escape from the surface. Due to the irregular and disordered porous structure, these enlarged bubbles frequently become trapped on the electrode, increasing contact resistance and ohmic losses. In contrast, PW exhibits superior bubble detachment performance, allowing bubbles to separate at smaller sizes while minimizing accumulation. Its aligned, low-tortuosity porous structure provides efficient bubble transport channels, facilitating rapid bubble detachment and preventing interfacial retention. This enhanced bubble transport, coupled with the high catalytic activity of Fe-NiWB, leads to lower ohmic resistance and enhanced overall cell performance.


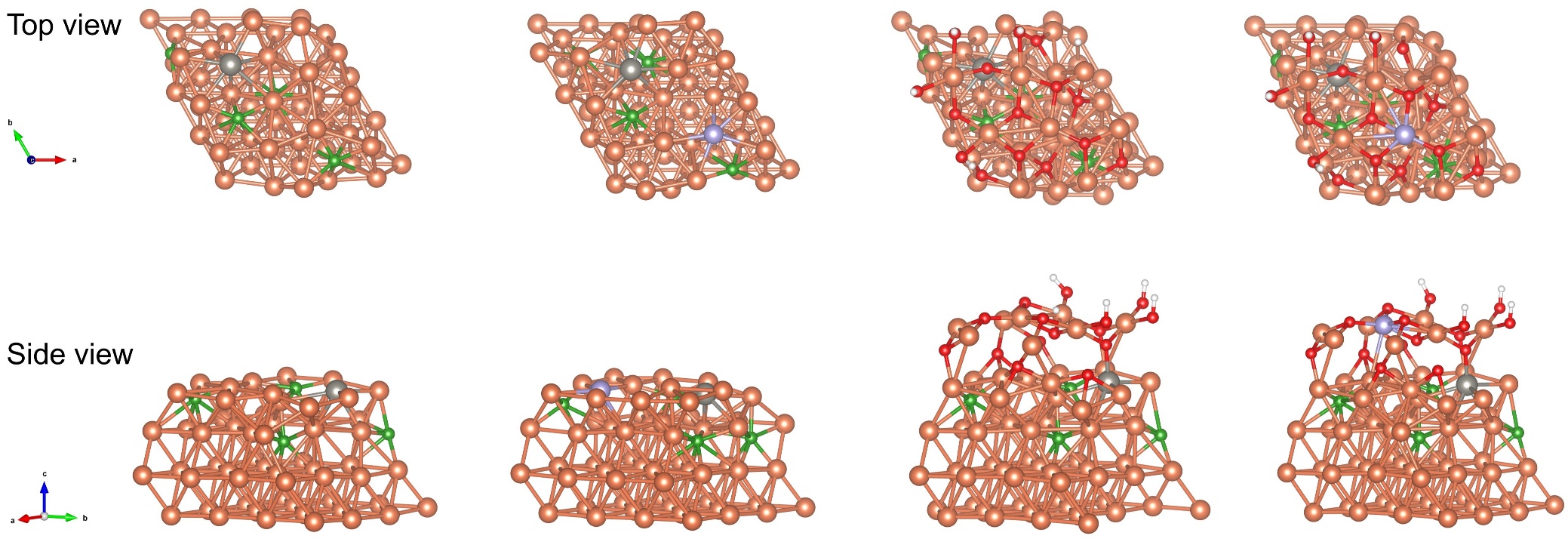


**Figure S39.** Optimized models of NiWB, Fe-NiWB, NiOOH/NiWB, and (Fe)NiOOH/NiWB (from left to right). The Ni, W, B, Fe, O, and H atoms are marked by brown, gray, green, blue, red, and white colors, respectively.


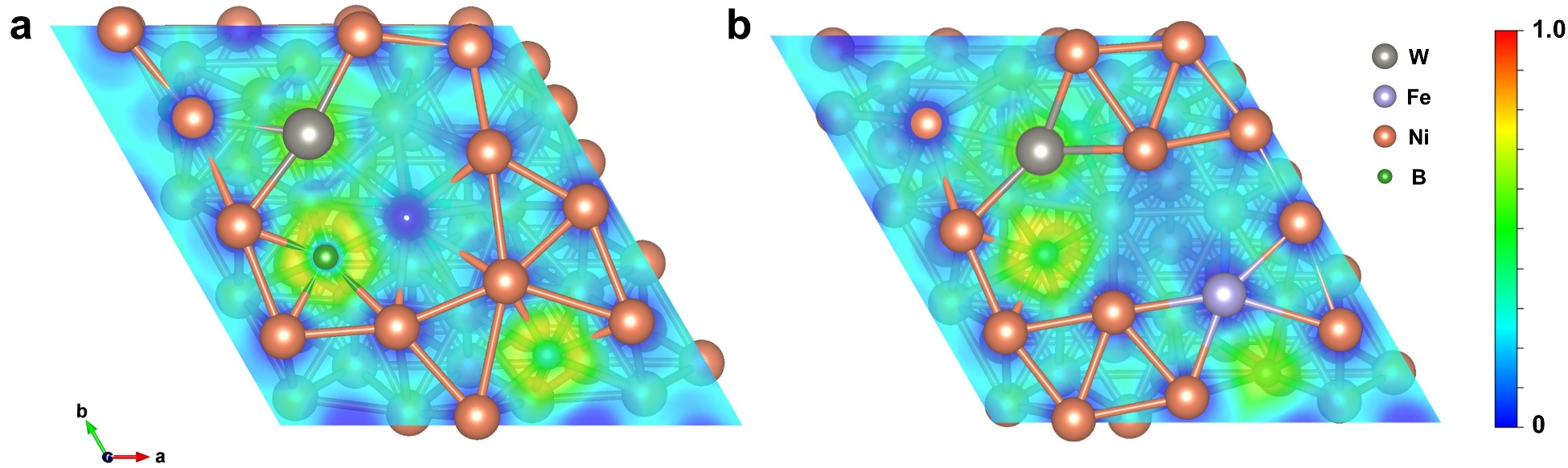


**Figure S40.** The electronic local function diagram of the a) NiWB and b) Fe-NiWB structure.


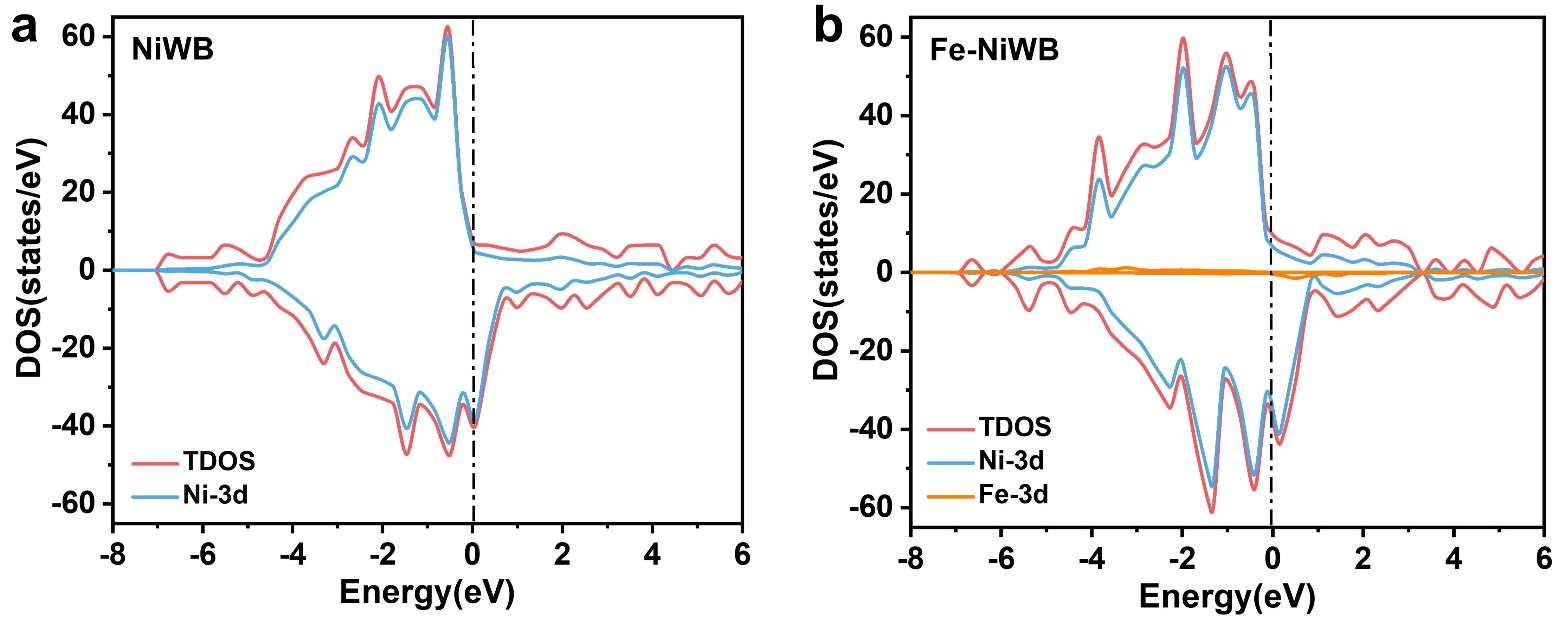


**Figure S41.** Electronic density of states (DOS) of a) NiWB and b) Fe-NiWB.


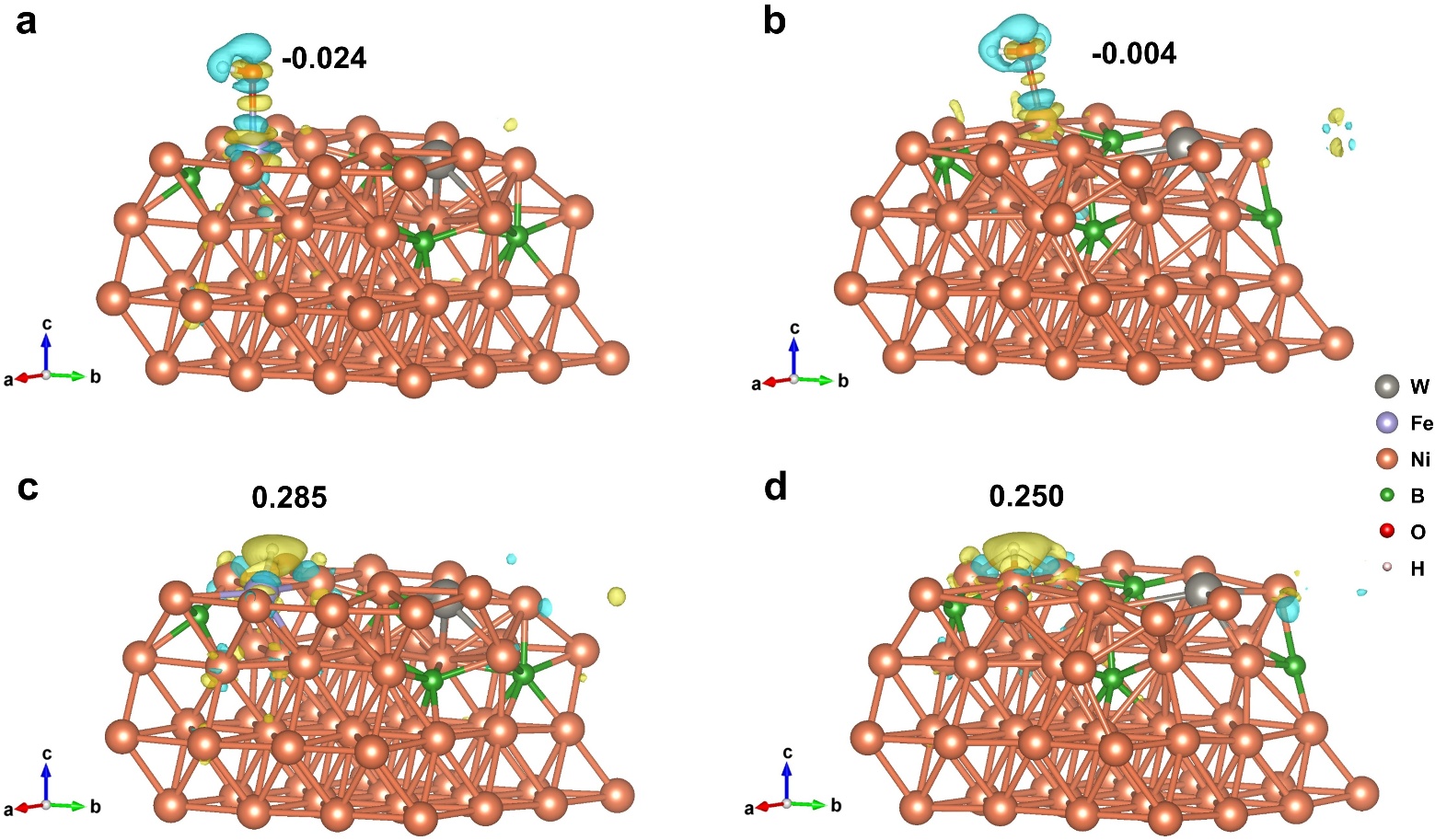


**Figure S42.** Differential charge density maps of H_2_O and H adsorption on Fe-NiWB and NiWB structures: a, b) H_2_O adsorption; c, d) H* adsorption.


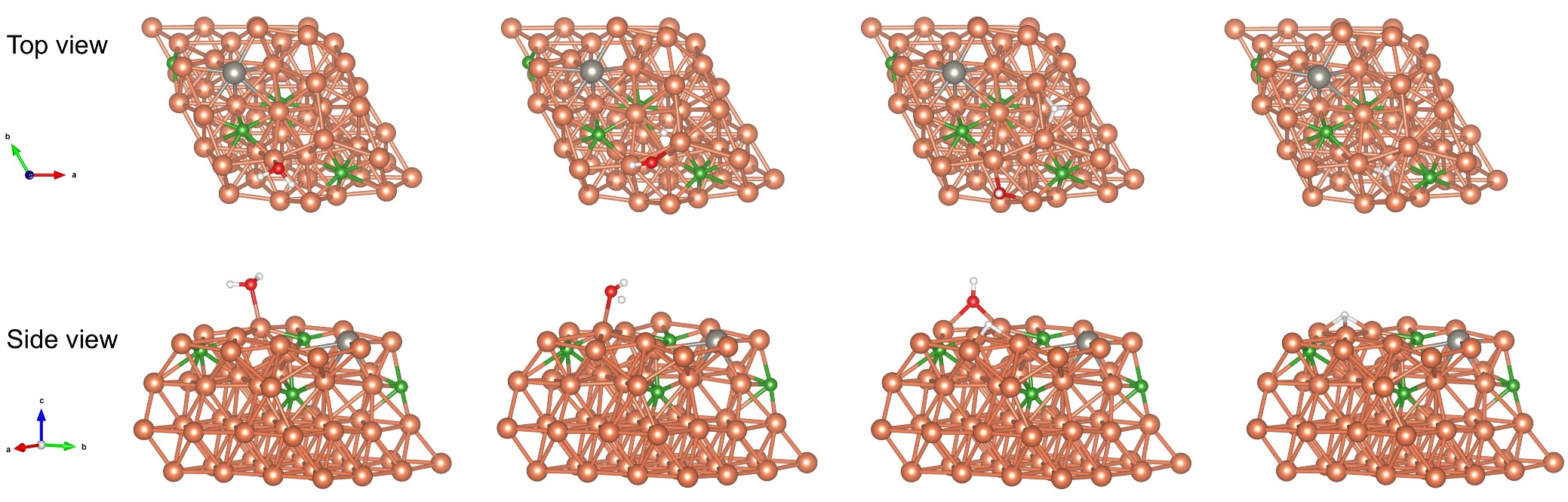


**Figure S43.** Optimized models of H_2_O, transition states, OH* + H*, and H* on NiWB (from left to right). The Ni, W, B, O, and H atoms are marked by brown, gray, green, red, and white colors, respectively.


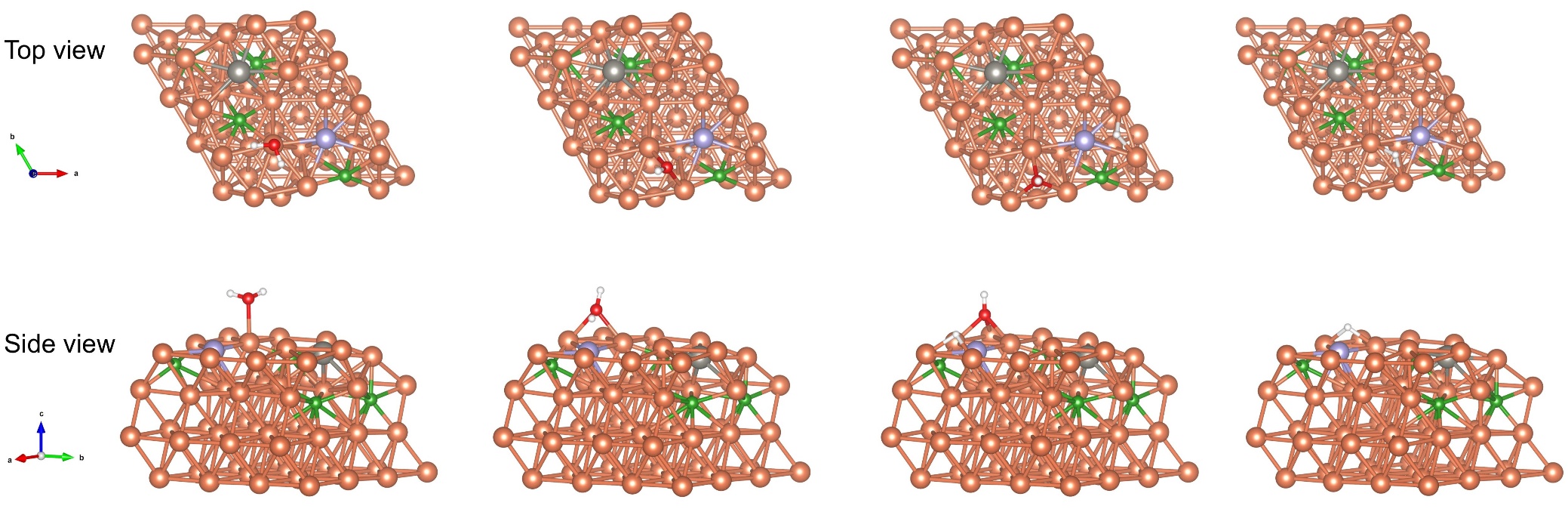


**Figure S44.** Optimized models of H_2_O, transition states, OH* + H*, and H* on the Ni site of Fe-NiWB (from left to right). The Ni, W, B, Fe, O, and H atoms are marked by brown, gray, green, blue, red, and white colors, respectively.


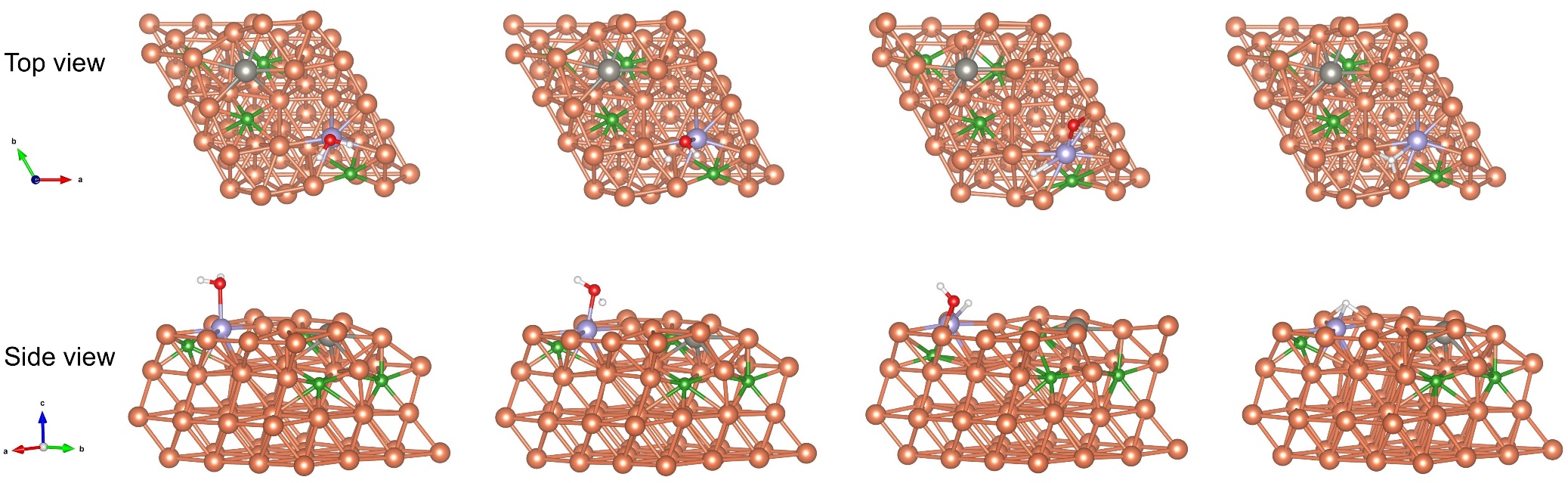


**Figure S45.** Optimized models of H_2_O, transition states, OH* + H*, and H* on the Fe site of Fe-NiWB (from left to right). The Ni, W, B, Fe, O, and H atoms are marked by brown, gray, green, blue, red, and white colors, respectively.


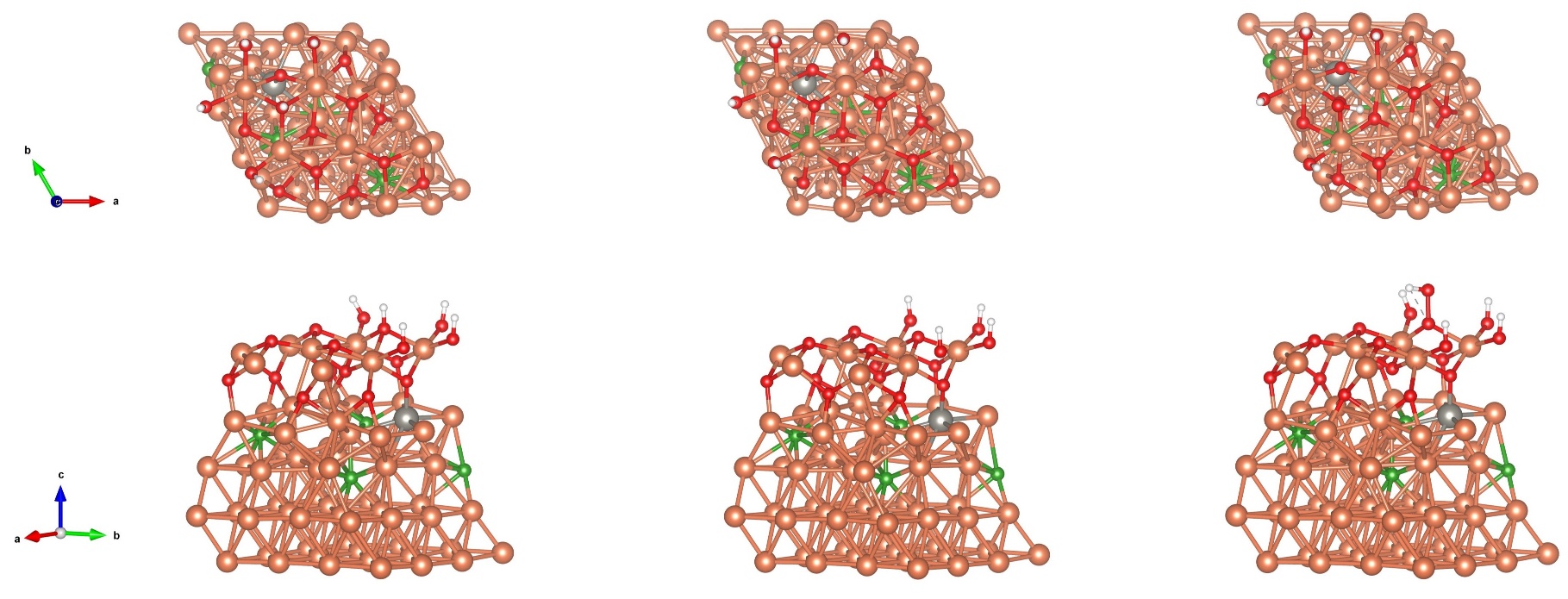


**Figure S46.** Optimized models of OH*, O*, and OOH* on NiOOH/NiWB (from left to right). The Ni, W, B, O, and H atoms are marked by brown, gray, green, red, and white colors, respectively.


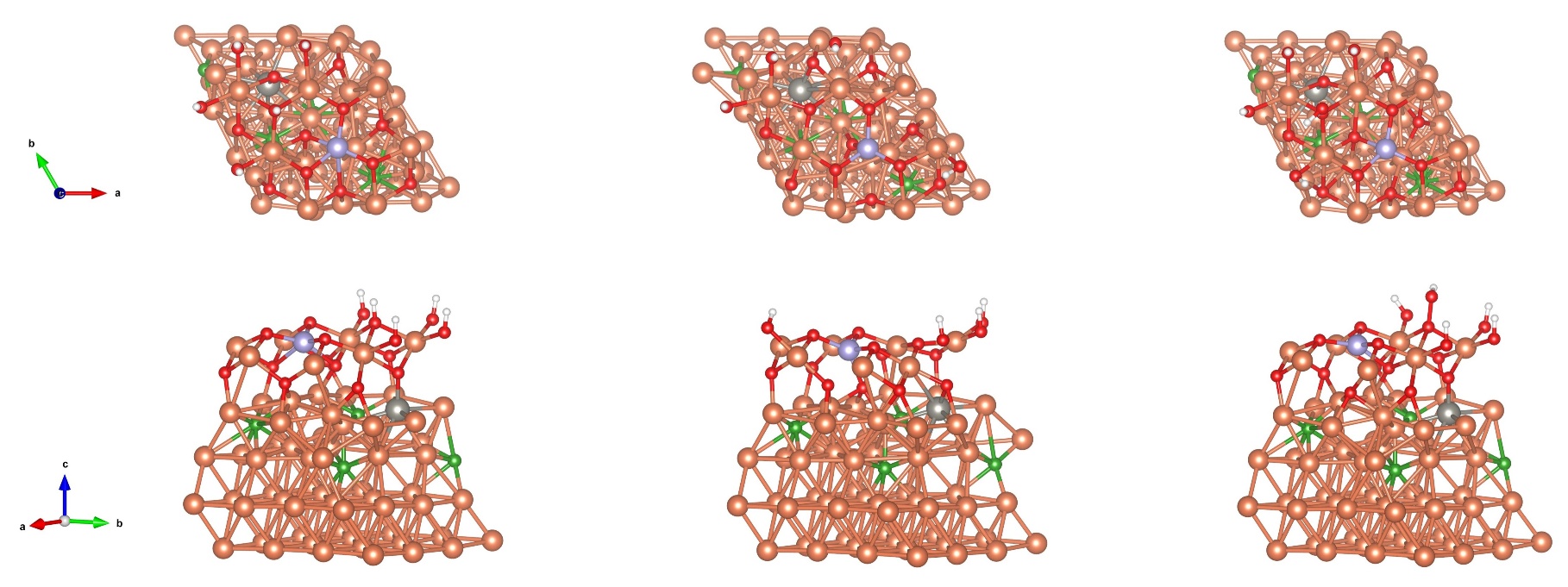


**Figure S47.** Optimized models of OH*, O*, and OOH* on the Ni site of Fe-NiOOH/NiWB (from left to right). The Ni, W, B, O, and H atoms are marked by brown, gray, green, red, and white colors, respectively.


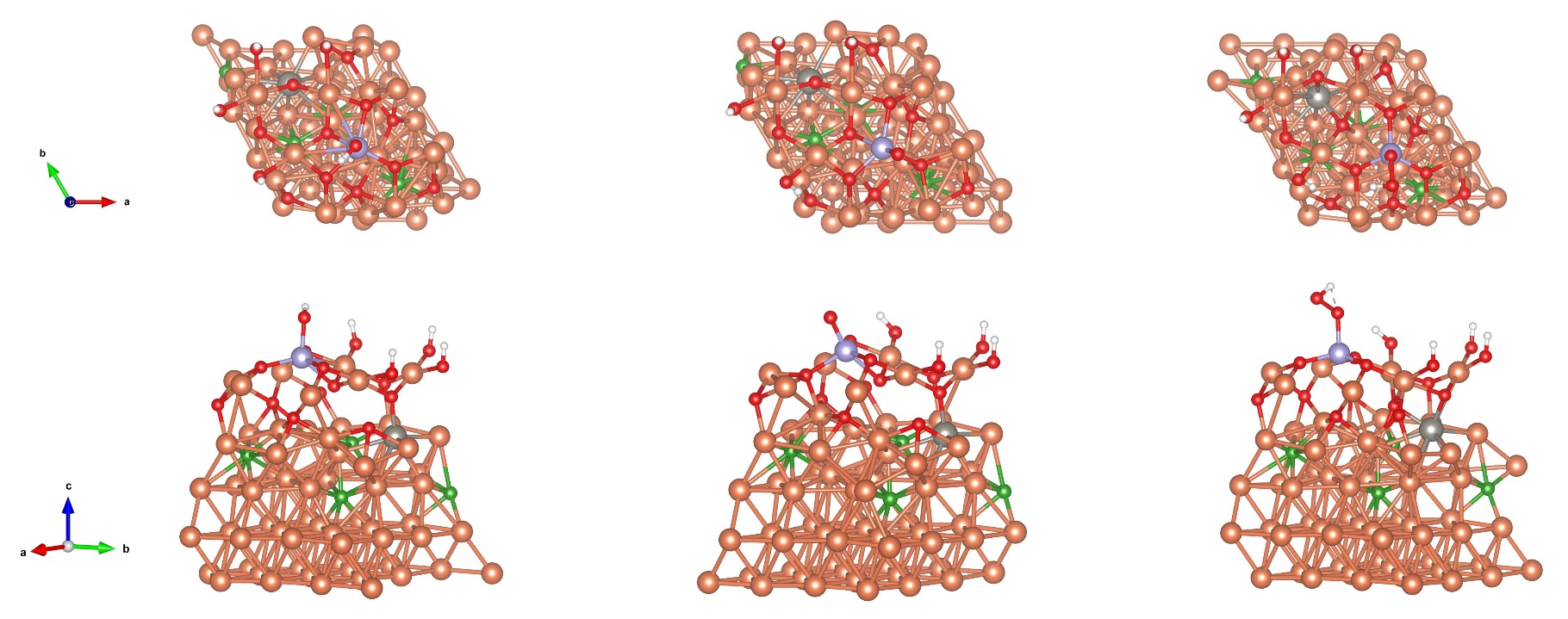


**Figure S48.** Optimized models of OH*, O*, and OOH* on the Fe site of Fe-NiOOH/NiWB (from left to right). The Ni, W, B, O, and H atoms are marked by brown, gray, green, red, and white colors, respectively.


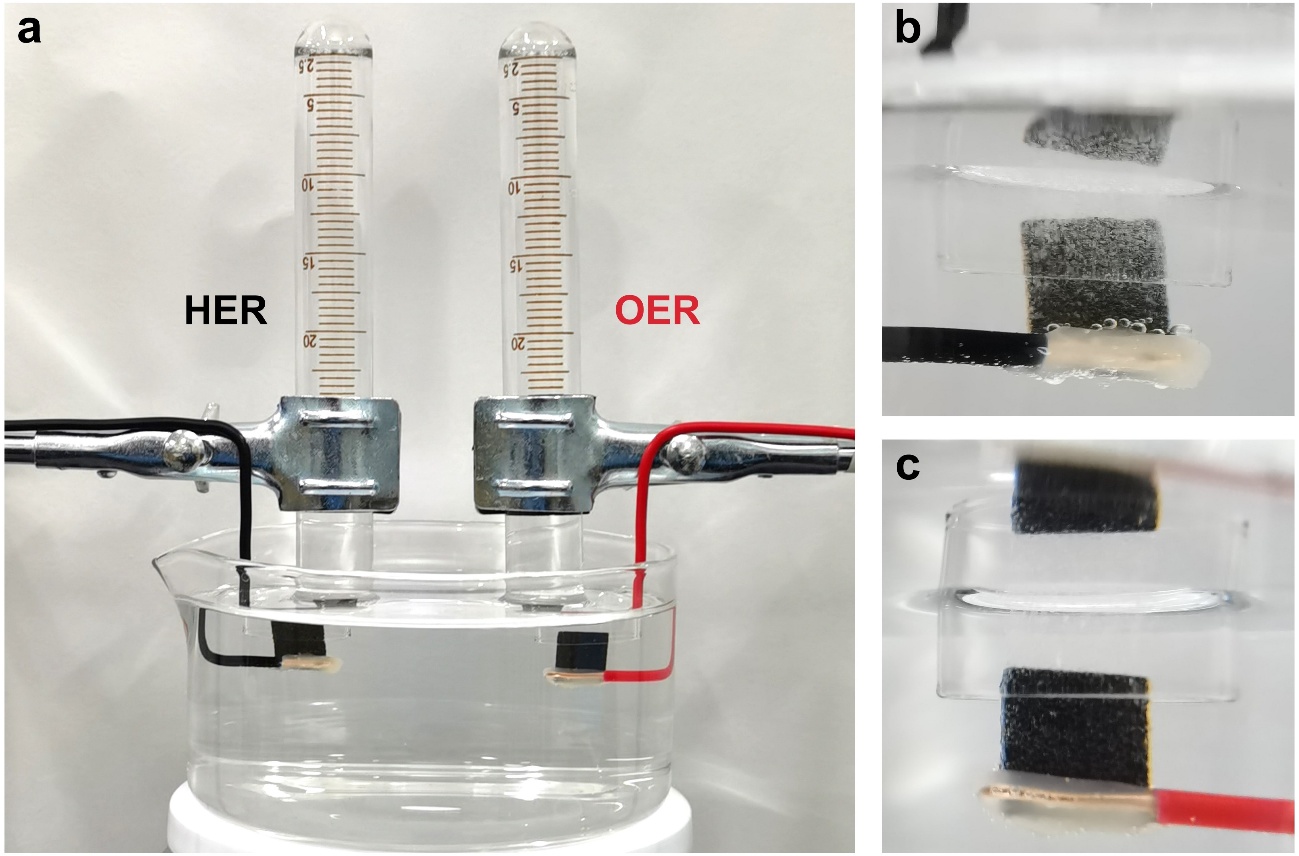


**Figure S49.** a) Photograph of the fabricated two-electrode device for farid efficient test demonstrating the evolution of H_2_ and O_2_ gas from the electrodes at the current density of 100 mA cm^−2^. b, c) Photographs of the gas volume measurement by inverted test tubes.


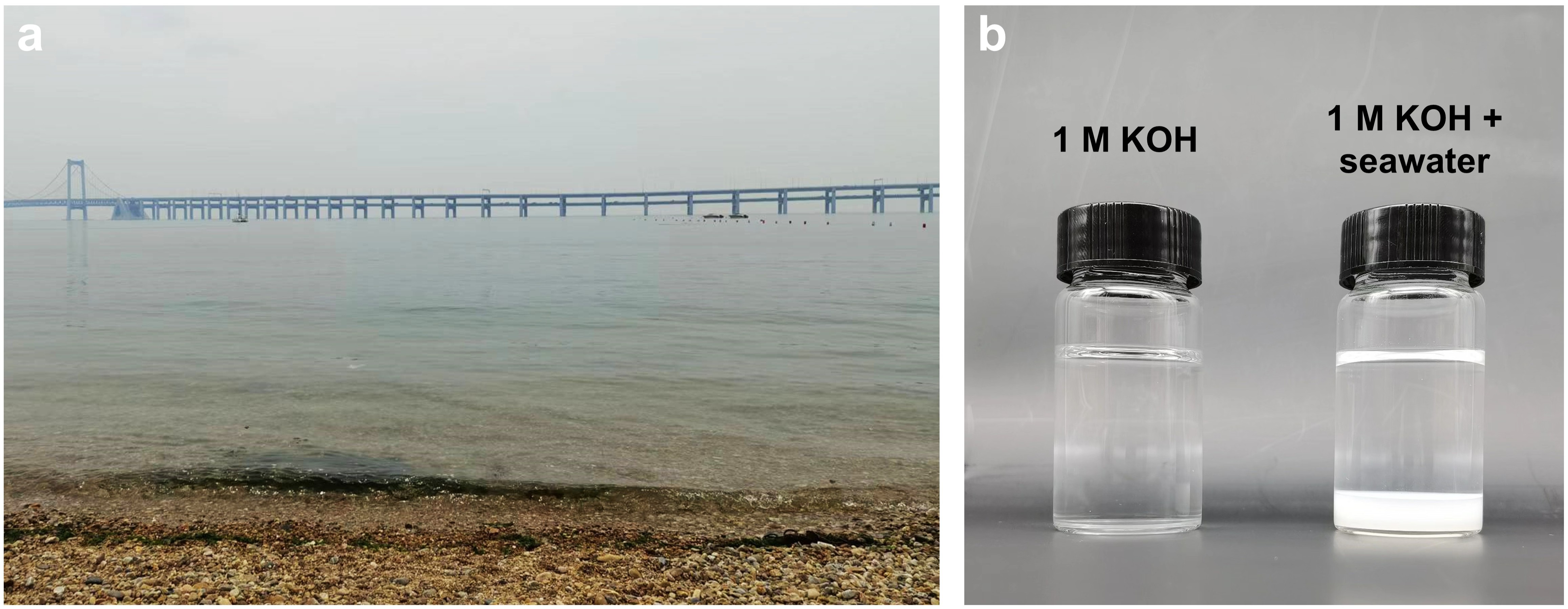


**Figure S50.** a) Photo of the Bohai Bay (Xinghai Park, Dalian, Liaoning, China) for natural seawater collecting. b) optical photographs of the 1 M KOH and 1M KOH + seawater. (Due to the formation of insoluble precipitates like Ca(OH)_2_ and Mg(OH)_2_ upon the addition of KOH to natural seawater, the electrolyte was left to stand and filtered.)


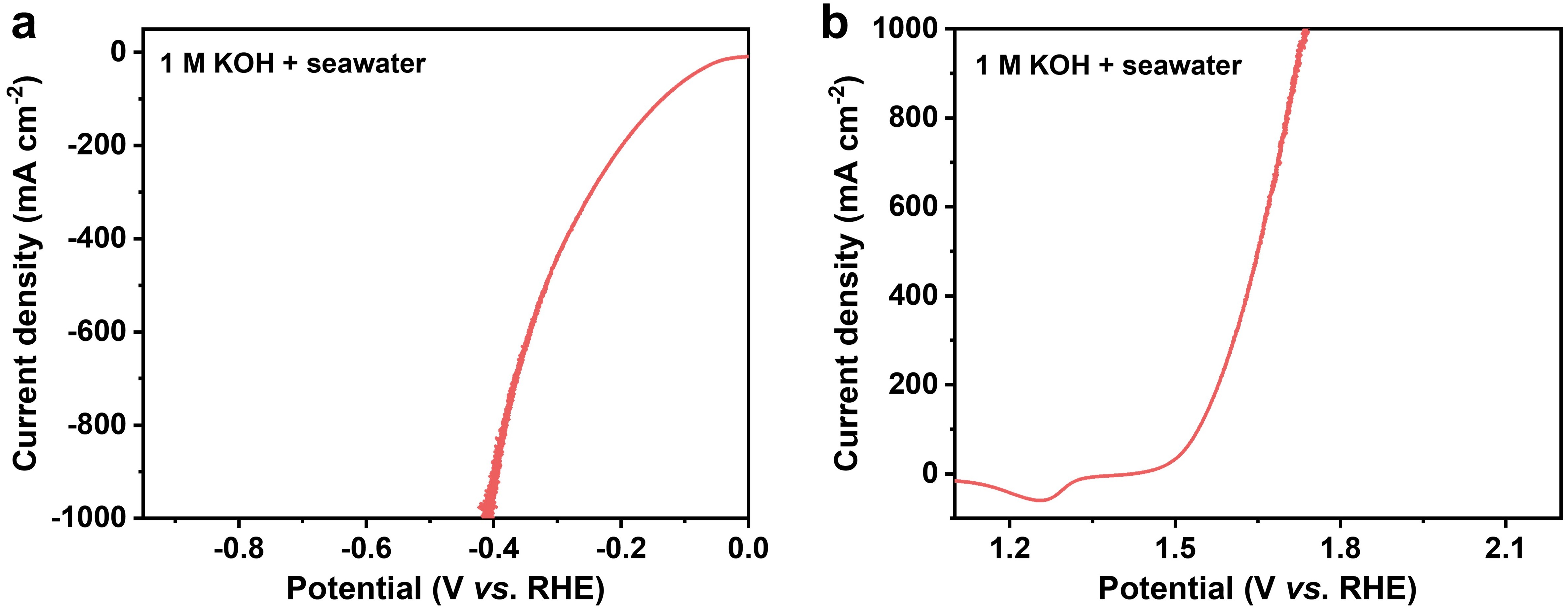


**Figure S51.** a) HER, and b) OER LSV curves of Fe-NiWB/PW in 1.0 M KOH + seawater.

**Note:** Compared with 1 M KOH, the Fe-NiWB/PW electrode shows a slightly higher operating voltage in alkaline seawater due to the presence of residual impurity ions (Mg^2+^/Ca^2+^) and chloride species competing with OER ^[14]^. Post-electrolysis characterizations reveal distinct surface reconstructions: after HER, a thin Ni(OH)_2_ layer forms, enhancing interfacial stability ^[14]^; after OER, the surface further oxidizes into (Fe)NiOOH phase that functions as both the active OER layer and a corrosion-resistant barrier against Cl^− [15]^. These dual adaptations explain the excellent durability of Fe-NiWB/PW under seawater electrolysis conditions (Figure 6g).


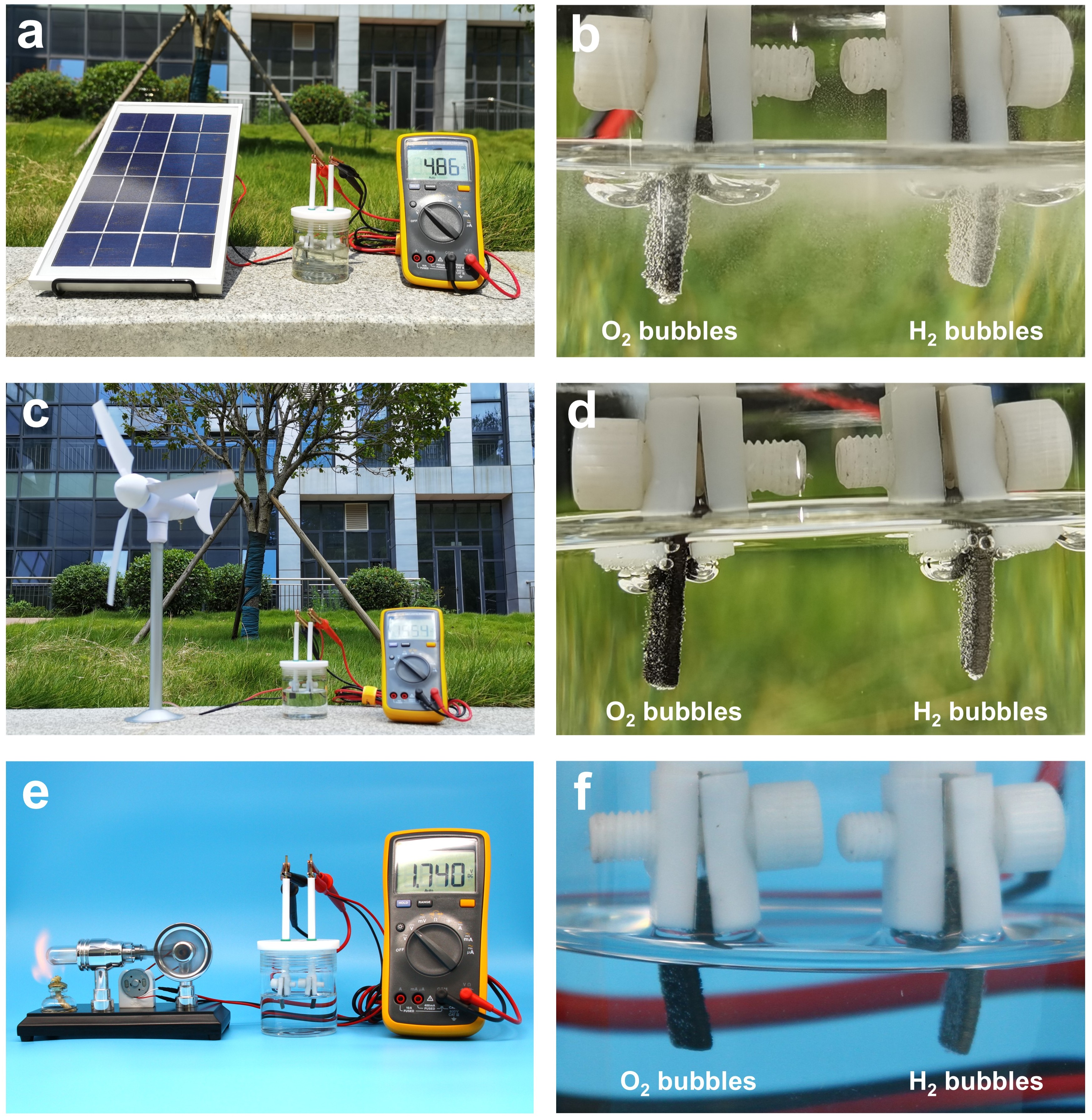


**Figure S52.** Photographs of Fe-NiWB/PW-based water electrolysis hydrogen production devices powered by a, b) a photovoltaic cell, c, d) a wind turbine and e, f) Stirling engine.


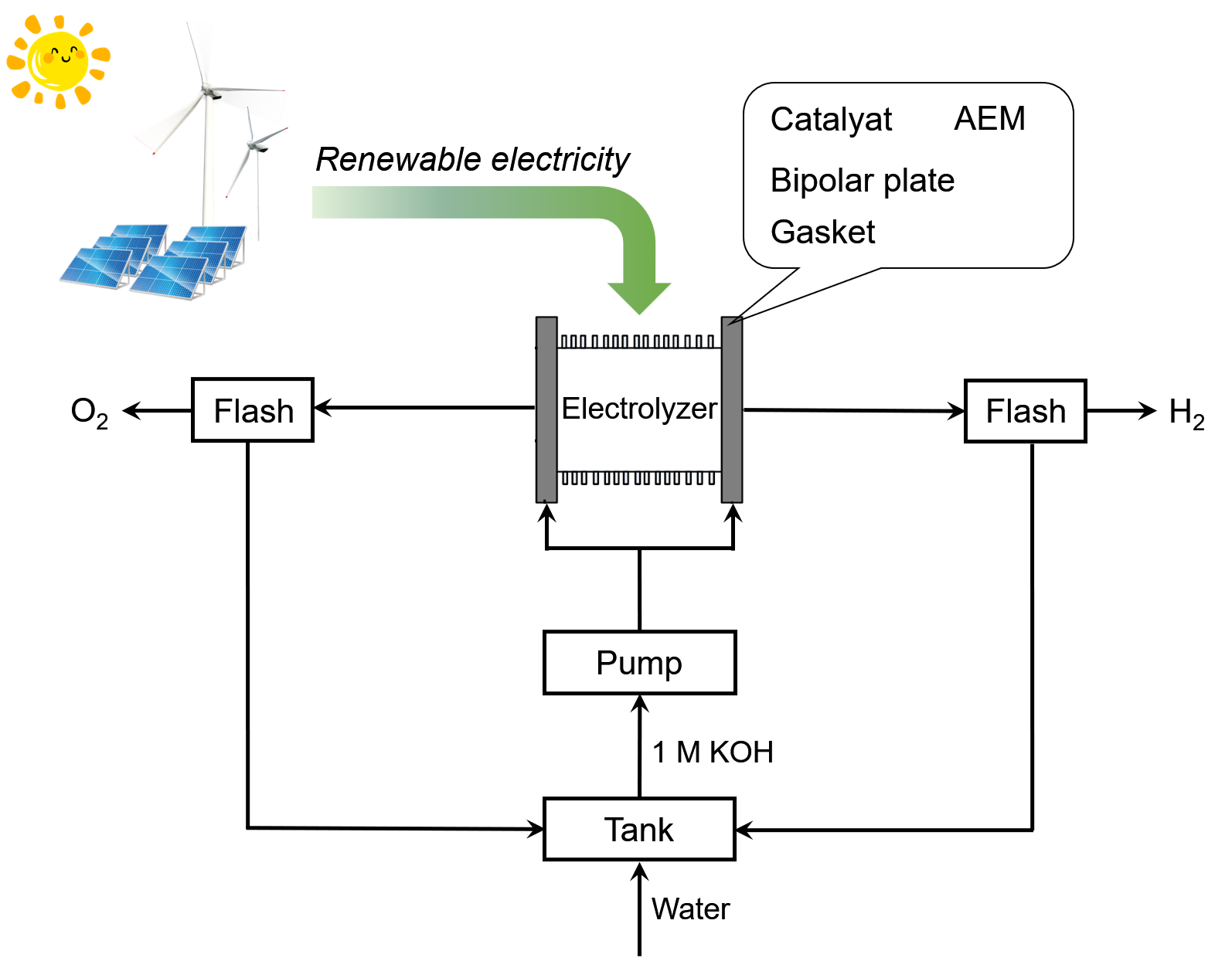


**Figure S53.** Schematic of a proposed green electricity-driven AEM electrolyzer plant.

| **Strategy** | **Principle & Process characteristics** | **Structural Features & Scalability** | **Representative Morphology** | **Main limitations** | **Representative references** |
| --- | --- | --- | --- | --- | --- |
| **Hard templating** | Metal deposition within a removable rigid template (e.g. Polystyrene, SiO_2_ spheres); followed by template dissolution. | Produces highly ordered and uniform pores defined by the template; limited scalability due to costly template synthesis/removal. | 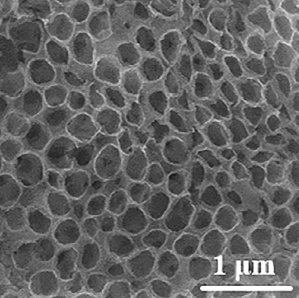 | Template synthesis and removal involve multiple steps, may cause framework collapse or residue contamination. | *Adv. Mater*. **2025**, e06068.^[16]^ |
| **Soft templating** | Surfactant micelles or block copolymers direct metal ion assembly into mesostructures; thermal or chemical removal of template. | Enables tunable mesopore size (2–50 nm) and compositional flexibility; limited by bath sensitivity and narrow operational window. | 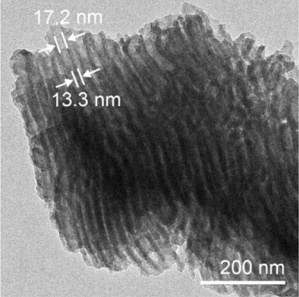 | Organic residues; structure sensitive to surfactant concentration and temperature fluctuations. | *Energy Environ. Sci*. **2025**, 18, 1756.^[17]^ |
| **Dynamic bubble templating** | Gas bubbles serve as dynamic templates during high-current electrodeposition. | Produces interconnected macropores (10–100 μm); simple and scalable on conductive substrate. | 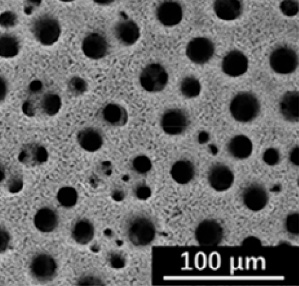 | The stochastic nature of bubble nucleation, coalescence, and detachment results in irregular pore distribution. | *ACS Nano* **2023**, 17, 22268.^[18]^ |
| **Dealloying / selective etching** | Selective leaching of the less-noble component from an alloy precursor. | Generates tunable nanoporous architectures (2–100 nm); pore size adjustable via etching duration; moderate scalability. | 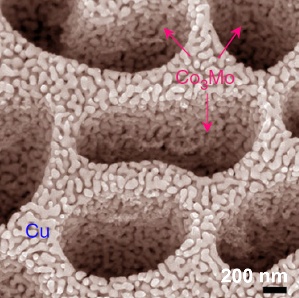 | Limited to specific alloy systems; structural brittleness and residual stress accumulation during dealloying. | *Nat. Commun*. **2020**, 11, 2940.^[19]^ |
| **Colloid-mediated electroless plating (CMEP)** | *In-situ* generated Fe-W-O colloids regulate nucleation and inhibit dense-layer growth within a single chemical bath. | Produces hierarchically porous, strongly anchored coatings; structure tunable via deposition time and bath chemistry; mild, low-cost, and scalable process. | 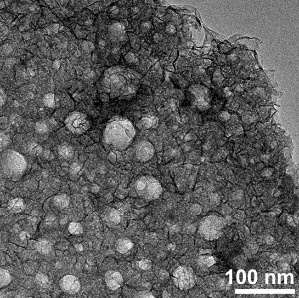 | Excessive colloid formation may induce localized inhomogeneity or mass-transfer limitation during plating. | **This work** |

**Table S1**. Comparison of representative fabrication strategies for porous metallic electrodes and the CMEP approach developed in this work.

**Table S2**. BET surface areas, main modal pore diameters, and total pore volumes

| **Sample** | **S_BET_**  **(m^2^ g^−1^)** | **Total pore volume**  **(cm^3^ g^−1^, P/P_0_=0.99)** | **BJH modal pore diameter (nm)** | **Pore range**  **(nm)** |
| --- | --- | --- | --- | --- |
| NiWB | 0.0867 | 0.000093 | ~3.21 | 10-80 |
| Fe-NiWB | 0.3494 | 0.001592 | ~3.85 | 3-100 |

**Table S3**. The atomic content of the as-prepared Fe-NiWB and NiWB was measured by ICP-OES.

| **Element** | **Content (wt%)** | |
| --- | --- | --- |
|  | **Fe-NiWB** | **NiWB** |
| **Ni** | 86.62 | 70.49 |
| **W** | 6.96 | 15.50 |
| **B** | 1.16 | 2.27 |
| **Fe** | 1.90 | — |

**Table S4**. EXAFS fitting parameters at the K-edge for various samples.

| **Sample** | **Bond type** | **CN^*^** | **R (Å)** | **σ^2^ (10^−3^Å^2^)^**^** | **R factor** |
| --- | --- | --- | --- | --- | --- |
| Ni foil | Ni-Ni | 12 | 2.48±0.02 | 6.9±0.3 | 0.002 |
| NiO | Ni-O | 5.8±0.3 | 2.09±0.03 | 5.5±0.2 | 0.008 |
|  | Ni-Ni | 11.3±0.4 | 2.95±0.02 | 4.3±0.1 |  |
| NiWB | Ni-Ni | 9.3±0.39 | 2.44±0.01 | 7.5±1.6 | 0.004 |
| Fe-NiWB | Ni-Ni | 8.6±1.0 | 2.48±0.02 | 9.0±3.0 | 0.004 |
|  | Ni-Fe | 2.9±0.5 | 2.50±0.02 | 5.0±4.0 |  |

^*^ CN: coordination number; S_0_^2^ was fixed to 0.82. R: distance between absorber and backscatter atoms. ^**^ σ^2^: Debye-Waller factors to account for both thermal and structural disorders. R factor: indicates the goodness of the fit.

**Table S5**. Comparison of ECSA, ECSA-normalized cuurent density, and TOF values for Fe-NiWB/PW catalysts prepared with different deposition times. The 120-min sample exhibits the highest ECSA and TOF for both HER and OER, confirming optimal active site exposure and intrinsic activity.

| **Catalyst** | **ECSA (cm^2^)** | ***J*_ECSA_ (mA cm^−2^) @ η = 300 mV** | **TOF (s^−1^) @ η = 300 mV** |
| --- | --- | --- | --- |
| Fe-NiWB/PW-60min | 85.25 | −0.48 @ HER | 0.81 @ HER |
|  |  | 0.06 @ OER | 0.04 @ OER |
| Fe-NiWB/PW-120min | 384.75 | −1.73 @ HER | 2.67 @ HER |
|  |  | 0.20 @ OER | 0.16 @ OER |
| Fe-NiWB/PW-180min | 335.75 | −0.77 @ HER | 1.19 @ HER |
|  |  | 0.15 @ OER | 0.12 @ OER |
| Fe-NiWB/PW-240min | 275.00 | −0.44 @ HER | 0.69 @ HER |
|  |  | 0.10 @ OER | 0.08 @ OER |

**Table S6**. Comparison of the HER performance of Fe-NiWB and other previously reported electrocatalysts in 1.0 M KOH electrolyte.

| **Catalyst** | **Substrate** | **Overpotential at**  **-10 mA cm^-2^ (mV)** | **Tafel slope**  **(mV dec^-1^)** | **Reference** |
| --- | --- | --- | --- | --- |
| **Fe-NiWB** | **Purified wood** | **48** | **88.43** | **This Work** |
| Zn-Fe/Mn@Mn-FeP ^[20]^ | Fe_70_Mn_28_Zn_2_ steel plate | 53 | 53.2 | *Energy & Environ. Sci.*, **2022**. |
| NiFeP_FA_NN ^[21]^ | Cu foam | 56 | 95 | *Appl. Catal. B*, **2023**. |
| Ni_2_P-CoCH ^[22]^ | Carbon paper | 62 | 76 | *Angew. Chem. Int. Ed*., **2023**. |
| CoFeP-N ^[23]^ | Ni foam | 64 | 80.02 | *Appl. Catal. B*, **2024**. |
| Fe_1.2_(CoNi)_1.8_Se_6_ ^[24]^ | Ni foam | 66 | 66 | *Adv. Energy Mater*., **2023**. |
| Ni(OH)_2_@Ni-N/Ni-C ^[25]^ | Ni foam | 68 | 43.9 | *Nat. Commun.*, **2023.** |
| P-NiCuFe_0.06_-LDH ^[26]^ | Ni foam | 71 | 58 | *Adv. Funct. Mater*., **2023**. |
| CoNi@N-PCNS ^[12b]^ | Ni foam | 71.2 | 137.4 | *Adv. Funct. Mater*., **2024**. |
| Ni_2_P/MoS_2_-CoMo_2_S_4_@C ^[27]^ | Ni foam | 73 | 46.3 | *ACS Nano* **2024**. |
| MoO_2_/Ni_3_S_2_ ^[28]^ | Ni foam | 74 | 85.2 | *Nano Energy*, **2024**. |
| Co/N-NiMo_3_S_4_ ^[29]^ | Carbon cloth | 78 | 69 | *Appl. Catal. B-Environ.*, **2023**. |
| CoTe_2_/CoP ^[30]^ | Ti mesh | 80 | 57 | *Appl. Catal. B*, **2023**. |
| Ce-CoP@CC^[31]^ | Carbon cloth | 81 | 68.7 | *Adv. Energy Mater*., **2023**. |
| Ni_2_P-NiMoO_x_ ^[32]^ | Ni foam | 91 | 60.1 | *ACS Catal.*, **2023**. |
| WC_1-x_/Mo_2_C@CNF ^[33]^ | Carbon nanofiber | 97 | 42.9 | *Angew. Chem. Int. Ed*., **2024**. |
| MoO_2_@E-MoS_2_ ^[34]^ | Carbon paper | 99 | 109 | *Appl. Catal. B*, **2024**. |
| Ni-BP-6 ^[35]^ | Ni Foam | 136 | 114.5 | *Nano-Micro Letters*, **2024**. |
| NFMN-Ni_2_P ^[36]^ | Ni Foam | 141 | 91 | *Adv. Funct. Mater*., **2024**. |
| NCoFO/CC-60 ^[37]^ | Carbon cloth | 203 | 66 | *ACS Catal.*, **2024***.* |

**Table S7**. Comparison of the OER performance of Fe-NiWB and other previously reported electrocatalysts in 1.0 M KOH electrolyte.

| **Catalytic material** | **Substrate** | **Overpotential at 10 mA cm^-2^ (mV)** | **Tafel slope**  **(mV dec^-1^)** | **Reference** |
| --- | --- | --- | --- | --- |
| **Fe-NiWB** | **Purified wood** | **248** | **42.59** | **This Work** |
| NiFe_FA_NN ^[21]^ | Cu foam | 256 | 56 | *Appl. Catal. B*, **2023**. |
| NFMN-FeOOH ^[36]^ | Ni foam | 256 | 36 | *Adv. Funct. Mater*., **2024**. |
| Ni_2_P-NiMoO_x_ ^[32]^ | Ni foam | 255.9 | 67.4 | *ACS Catal.,* **2023**. |
| FeCo_3_(DDA)_2_ ^[38]^ | Carbon cloth | 260 | 46.86 | *Adv. Mater*., **2024**. |
| CoTe_2_/CoP ^[30]^ | Ti mesh | 260 | 89 | *Appl. Catal. B,* **2023**. |
| CoNi@N-PCNS ^[12b]^ | Ni foam | 263.8 | 59.7 | *Adv. Funct. Mater*., **2024**. |
| CoFeV ^[39]^ | Carbon paper | 266 | 73 | *Adv. Funct. Mater*., **2024**. |
| CoCrOx ^[40]^ | Carbon paper | 268 | 101.6 | *Nat. Commun.,* **2024**. |
| P-NiFe_2_O_4_ ^[41]^ | Carbon paper | 268 | 43.3 | *Appl. Catal. B,.* **2024**. |
| Fe-NiO/NiS_2_ ^[42]^ | Carbon paper | 270 | 40 | *Angew. Chem. Int. Ed*., **2022**. |
| Ni_2_P-CoCH^[22]^ | Carbon paper | 270 | 76 | *Angew. Chem. Int. Ed*., **2023**. |
| Ni_8_Co_2_-BDC ^[43]^ | Carbon cloth | 274 | 73.1 | *Adv. Funct. Mater*., **2024**. |
| Co-O NSs-2 nm ^[44]^ | Glass carbon | 278 | 78 | *ACS Nano* **2023**. |
| Ir_1_Ni@MoO_2_ SAAs ^[45]^ | Glass carbon | 280 | 91 | *Adv. Mater*., **2024**. |
| Co_3_Mo/Mo_2_C@NC ^[46]^ | Carbon paper | 282 | 59.3 | *Adv. Funct. Mater*., **2024**. |
| Pt_SA_-Mn,Fe-Ni LDHs ^[47]^ | Ni foam | 288 | 28 | *ACS Nano* **2024**. |
| CoNiFeCu (1:1:1:0.5) ^[48]^ | Glass carbon | 291 | 43.9 | *Adv. Mater*., **2022**. |
| (NiCo)S_1.33_ ^[49]^ | Glass carbon | 302 | 118 | *Nat. Commun.,* **2023**. |
| CoHF/P-O ^[50]^ | Glass carbon | 312 | 53 | *Adv. Funct. Mater*., **2023**. |

**Table S8**. Computing results of TDOS.

| **Structure** | **TDOS (eV)** | **Ni-d center (eV)** | **Fe-d center (eV)** |
| --- | --- | --- | --- |
| NiWB | −0.997 | −1.012 |  |
| Fe-NiWB | −0.908 | −1.005 | −0.713 |
| NiOOH/NiWB | −1.106 | −1.120 |  |
| Fe-NiOOH/NiWB | −1.103 | −1.117 | −1.454 |

**Table S9**. Cell voltage comparison between our developed anion exchange membrane (AEM) water electrolyzer and recent reports.

| **Catalyst** | **Substrate** | **Voltage (V)**  **@500 mA cm^−2^** | **Membrane** | **Temperature** | **Reference** |
| --- | --- | --- | --- | --- | --- |
| **Fe-NiWB/PW_(+,-)_** | **Wood** | **1.55** | **FAB-PK-130,**  **Fumasep** | **60 ℃** | **This Work** |
| Ru-Ru_2_P/V_2_CT_x(-)_\|\|RuO_2(+)_ ^[51]^ | Ni foam | ~1.60 | X37-50, Dioxide | 60 ℃ | *Appl. Catal. B*, **2024**. |
| Pt/C_(-)_\|\|Ti-NFO_(+)_ ^[52]^ | Ni foam | 1.61 | Piperion-A20, Versogen | 60 ℃ | *ACS Catal.*, **2024***.* |
| Pt/C_(-)_\|\|FCO1.5_(+)_ ^[53]^ | Carbon paper | ~1.63 | X37-50, Dioxide | 60 ℃ | *Adv. Funct. Mater*., **2024**. |
| Ru-LC-Ni(OH)_2(-)_\|\|FeNi LDH_(+)_ ^[54]^ | Ni foam | 1.65 | N.A. | 60 ℃ | *Angew. Chem. Int. Ed.*, **2024**. |
| Pt/C_(-)_\|\|Ni_3_Fe LDH_(+)_ ^[55]^ | Carbon paper_(-)_/  Ni fiber_(+)_ | ~1.66 | DURAION, Evonik | 55 ℃ | *Adv. Funct. Mater*., **2022**. |
| Pt/C_(-)_\|\|Bi/BiCeO_1.8_H_(+)_ ^[56]^ | Carbon paper | ~1.68 | X37-50, Dioxide | 50 ℃ | *Adv. Mater*., **2024**. |
| UP-RuNi_SAs(-)_/C\|\|NiFeO_x(+)_ ^[57]^ | Carbon paper | 1.70 | N.A. | 70 ℃ | *Nat. Commun.*, **2024**. |
| Ru/P-MoB_(-)_\|\|RuO_2(+)_ ^[58]^ | NiFe foam | 1.71 | X37-50, Dioxide | 60 ℃ | *Adv. Energy Mater*., **2024**. |
| 1‰Pt-NiCoP@MXene_(-)_\|\|NiFe LDH_(+)_ ^[59]^ | Carbon cloth | 1.76 | X37-50, Dioxide | 65 ℃ | *Angew. Chem. Int. Ed.*, **2024**. |
| Pt_SA_-Mn,Fe-Ni LDHs_(+,-)_ ^[47]^ | Ni foam | 1.79 | X35-50, Dioxide | 60 ℃ | *ACS Nano.*, **2024***.* |
| Pt-AC/Cr-N-C_(-)_\|\|NiFe LDH_(+)_ ^[60]^ | Carbon paper | 1.80 | X37-50, Dioxide | 80 ℃ | *J. Am. Chem. Soc.* **2023.** |
| Ni(Cu)Fe_(+,-)_ ^[61]^ | Ni foam | 1.81  @400 mA cm^−2^ | FAA-3-PK-130,  Fumasep | 50 ℃ | *Chem Catal.*, **2023**. |
| NiMoN/NiFe LDH_(+,-)_ ^[62]^ | Ni foam | 1.85 | Piperion-A40, Versogen | 60 ℃ | *Nat. Commun.*, **2023**. |
| NA-Ru_3_Ni/C_(+,-)_ ^[63]^ | Ni foam | ~1.86 | X37-50, Dioxide | 60 ℃ | *Energy Environ. Sci.*, **2023***.* |
| (Ni,Fe)_3_S_2(+,-)_ ^[64]^ | NiFe foam | ~1.87  (30% KOH) | TN-ICM500, Carbon Energy | 80 ℃ | *Adv. Funct. Mater*., **2024**. |
| NiMoCo_(-)_\|\|NiFeCr-LDH_(+)_ ^[65]^ | Ni foam | 1.90 | X37-FA, Dioxide | 40 ℃ | *Small,* **2022**. |
| Pt_1_/Co(OH)_2_\|\|Ir_1_/Co(OH)_2_ ^[66]^ | Ni foam | 2.00  @400 mA cm^−2^ | N.A. | 75℃ | *Angew. Chem. Int. Ed.*, **2023**. |
| NiFeP_FA_NN_(-)_\|\|NiFe_FA_NN_(+)_ ^[21]^ | Cu foam | 2.14 | FAA-3-PE-30,  Fumasep | 60 ℃ | *Appl. Catal. B*, **2023**. |
| Pt@S-NiFe LDH_(-)_\|\| S-NiFe LDH_(+)_ ^[67]^ | Ni foam | 2.50 | FAA-3-PK-130,  Fumasep | N.A. | *Adv.. Mater.*, **2023**. |

**Table S10**. Metal abundance, yield, and price.

| **Metal** | **Crustal Abundance** | **Yield (t year**^−1^**)** | **Cost (¥ t**^−1^**)** |
| --- | --- | --- | --- |
| Ni | 6.0*10^−3^% | 3,520,000 | 127,000 |
| W | 1.1*10^−4^% | 78,000 | 143,400 |
| Fe | 6.3% | 1,286,540,000 | 2,470 |
| Pt | 3.7 *10^−6^% | 158 | 227,450,000 |
| Ru | 9.9 *10^−8^% | 12 | 117,000,000 |

**Note:** In the context of industrial-scale alkaline water electrolysis, the Fe-NiWB/PW electrode offers significant advantages due to its composition of abundant, low-cost elements (Tables S10, 11). The estimated manufacturing cost of the Fe-NiWB/PW electrode is 1058.17 ¥ m^−2^, significantly lower than that of commercial catalysts, such as 20% Pt/C (7211.48 ¥ m^−2^) and RuO_2_ (6611.48 ¥ m^−2^). Combining outstanding catalytic activity, durability, and economic feasibility, Fe-NiWB/PW represents a highly promising substitute for noble metal-based catalysts in industrial-scale water-splitting applications.

**Table S11**. The preparation cost of the electrode per unit area. 1.0 m^2^ noble metal catalysis (loading mass 1.0 mg cm^−2^).

| **Material** | **Supplier** | **Used account (unit)** | **Cost (¥ unit)** | **Cost (¥)** | **Sum cost (¥)** |
| --- | --- | --- | --- | --- | --- |
| Balsa wood | Zhuhai Yoongsun | 1.0 m^2^ | 124.00 | 124.00 | 1058.17 |
| Sodium hypochlorite  (NaClO) | Sinopharm | 5.0 L | 44.00 | 220.00 |  |
| Ethanol  (C_2_H_6_O) | Heng xing | 10.0 L | 21.00 | 210.00 |  |
| Sodium borohydride  (NaBH_4_) | Kelong | 37.8 g | 5.60 | 211.68 |  |
| Nickel sulfate  (NiSO_4_•6H_2_O) | Sinopharm | 376.0 g | 0.17 | 63.92 |  |
| Sodium tungstate (Na_2_WO_4_•6H_2_O) | Sinopharm | 15.0 g | 0.76 | 11.40 |  |
| Ammonium ferrous sulfate  ((NH_4_)_2_Fe(SO_4_)_2_•6H_2_O) | Sinopharm | 5.0 g | 0.05 | 0.25 |  |
| Sodium succinate  (C_4_H_4_Na_2_O_4_•6H_2_O) | Sinopharm | 250.0 g | 0.17 | 42.50 |  |
| Sodium sulfate  (Na_2_SO_4_) | Sinopharm | 150.0 g | 0.03 | 4.50 |  |
| Dimethylamine borane  (C_2_H_7_N•BH_3_) | Macklin | 72.0 g | 2.36 | 169.92 |  |
| Ni foam | Changde Liyuan | 1.0 m^2^ | 560.00 | 560.00 | 6611.48/  7211.48 |
| 20% Pt/C | Johnson Matthey | 10.0 g | 600.00 | 6000.00 |  |
| RuO_2_ | Macklin | 10.0 g | 540.00 | 5400.00 |  |
| Nafion D520 | Dupont | 40.0 mL | 15.00 | 600.00 |  |
| Isopropyl | Sinopharm | 260.0 mL | 0.20 | 51.48 |  |

**Note:** Techno-economic analysis (TEA) was conducted to evaluate the economic feasibility of the AEM electrolyzer (AEMEL) using the Fe-NiWB/PW electrode. The breakdown of hydrogen production cost was presented based on an ideal 1 MW-scale AWE plant, assuming complete performance retention from lab-scale tests to the plant (Figure S54). Annual expense of the plant was separated into capital expenditure (CAPEX) and operational expenditure (OPEX), where CAPEX was further separated into the capital costs and equipment costs. Some assumptions were made for the presented calculations (Table S12).

Sensitivity analysis is a critical component of economic assessments for various electrolytic hydrogen production projects. This approach systematically evaluates key sources of uncertainty that could substantially affect the economic viability of investment projects. By conducting sensitivity analyses, it becomes possible to identify and quantify the impact of market fluctuations, cost variations, and other uncertainties, thereby enhancing the accuracy of economic forecasts. To assess the influence of different parameters on H_2_ production costs, this study investigates catalyst durability, electricity costs, stack cost, and cell voltage as key factors.

From the preceding cost analysis, electricity cost emerges as the dominant operational expense in AEMEL based hydrogen production.

**Table S12**. Basic assumptions for techno-economic analysis.

| **Parameters** | **Value** | **units** | **Ref.** |
| --- | --- | --- | --- |
| Annual operating hours | 8000 | h y^−1^ |  |
| Plant life (N) | 20 | y |  |
| Income tax (IT) | 0.25 |  | [68] |
| Nominal Interest Rate (NIR) | 5 | % |  |
| Depreciation Method | Straight line |  |  |
| Depreciated years | 10 | y |  |
| Depreciation rate | 10 | % |  |
| Calculation scales (hydrogen production rate) | 3000 | Nm^3^ h^−1^ |  |
| Voltage | 1.64 | V |  |
| Current density | 1000 | mA cm^−2^ |  |
| Cathode FE | 100% |  |  |
| Anode FE | 100% |  |  |
| AEM lifetime | 10000 | h |  |
| Catalyst lifetime | 2000 | h |  |
| Area | 718 | m^2^ electrolyzer | a |
| Stack cost | 4000 | $ m^−2^ | [69] |
| Balance of plant (BOP) | 50 | % stack cost | [70] |
| H_2_ price | 2500 | $ t^−1^ | [71] |
| O_2_ price | 40 | $ t^−1^ | [72] |
| Electricity price | 0.02 | $ kwh^−1^ | [73] |
| Water price | 1.18 | $ t^−1^ | [74] |
| AEM price | 1127 | $ m^−2^ | b |
| Catalyst price | 149 | $ m^−2^ |  |

a: Calculation scales (3000 Nm^3^ h^−1^)/3600/22.4 (kmol m^−3^)*2*96485*1000/Current density(A/m^−2^)/CathodeFE

b: Assume exchange rate 7.1(¥/$).

**CAPEX**

**Table S13**. System capital cost calculation and reference.

| **Capital costs** | **Value** | **units** | **Ref.** |
| --- | --- | --- | --- |
| Inside battery limits (ISBL) | 7178943 | $ | Table S12 |
| Offsite battery limits (OSBL) | 2871577 | $ | 40% of ISBL |
| Engineering and construction costs | 1005052 | $ | 10% of ISBL+OSBL |
| Contingency charges | 1005052 | $ | 10% of ISBL+OSBL |
| Fixed Capital Investment (FCI) | 12060625 | $ | Sum above |

**Supplementary Table 14**. Equipment cost and reference.

| **Equipment** | **Value** | **units** | **Ref.** |
| --- | --- | --- | --- |
| Stack | 2871577 | $ | stack cost (4000 $ m^−2^)*area(718m^2^) |
| Balance of plant (BOP) | 1435789 | $ | 50% Stack ^[70]^ |
| install | 2871577 | $ | Stack*lang factor ^[70]^ |
| Total plant ISBL | 7178943 | $ | Sum above |

**OPEX&REVENUE**

OPEX consists of variable costs and fixed costs. Variable costs include expenses related to raw materials, consumables, and utilities, while fixed costs encompass maintenance, employee salaries, and insurance expenses.

**Table S15**. Mass flow data.

| **Materials** | **Value** | **units** | **Ref.** |
| --- | --- | --- | --- |
| Water | 2413.393 | kg/h | O_2_/16*18.02 |
| H_2_ | 270.5357 | kg/h | H_2_ yield (Nm^3^ h^−1^)/22.4(kmol m^−3^)*2.02(kg kmol^−1^) |
| O_2_ | 2142.857 | kg/h | Electrolyzer area (m^−2^)*Current density(A m^−2^)/ 96485/4*3600*32 /1000*Anode FE |

**Table S16**. Energy flow data.

| **Energy consumption** | **Value** | **units** | **Ref.** |
| --- | --- | --- | --- |
| Electrolytic energy consumption | 11773 | kw | Stack area (m^2^)*Current density (A m^−2^) *Voltage (V)/1000 |
| Pump consumption | 579 | kw | Water consumption (kg h^−1^)*(^a^ratio of circulating water to replenishment)300*0.8(kwh t^−1^)/1000 |

^a^: Derived from industrial examples in Aspen Plus

**Table S17**. Consumables data.

| **Consumables** | ^a^**Cost** $ | **Lifetime h** | ^b^**Annual expenditure $ y^−1^** |
| --- | --- | --- | --- |
| AEM | 808895 | 10000 | 647116 |
| Catalyst | 106994 | 2000 | 427974 |

^a^: Price * Area

^b^: Cost/Lifetime * years of operation time

**Table S18**. Net Present Value (NPV) Calculation Results.

| **Year** | **CAPEX ($)** | **REV ($)** | **CCOP ($)** | **GP ($)** | **Depreciation charge ($)** | **Taxable income ($)** | **Taxes paid ($)** | **Cash Flow ($)** | **Cash flow (Present Value) ($)** | **Net Present Value ($)** |
| --- | --- | --- | --- | --- | --- | --- | --- | --- | --- | --- |
| 0 | -12663656 | 0 | 0 | 0 | 0 | 0 | 0 | -12663656 | -12663656 | -12663656 |
| 1 |  | 5410714 | -2788361 | 2622353 | 1206063 | 1416291 | 0 | 2622353 | 2497479 | -10166177 |
| 2 |  | 5410714 | -2788361 | 2622353 | 1206063 | 1416291 | 354073 | 2268281 | 2057397 | -8108779 |
| 3 |  | 5410714 | -2788361 | 2622353 | 1206063 | 1416291 | 354073 | 2268281 | 1959426 | -6149353 |
| 4 |  | 5410714 | -2788361 | 2622353 | 1206063 | 1416291 | 354073 | 2268281 | 1866120 | -4283233 |
| 5 |  | 5410714 | -2788361 | 2622353 | 1206063 | 1416291 | 354073 | 2268281 | 1777257 | -2505976 |
| 6 |  | 5410714 | -2788361 | 2622353 | 1206063 | 1416291 | 354073 | 2268281 | 1692626 | -813350 |
| 7 |  | 5410714 | -2788361 | 2622353 | 1206063 | 1416291 | 354073 | 2268281 | 1612025 | 798675 |
| 8 |  | 5410714 | -2788361 | 2622353 | 1206063 | 1416291 | 354073 | 2268281 | 1535262 | 2333936 |
| 9 |  | 5410714 | -2788361 | 2622353 | 1206063 | 1416291 | 354073 | 2268281 | 1462154 | 3796090 |
| 10 |  | 5410714 | -2788361 | 2622353 | 1206063 | 1416291 | 354073 | 2268281 | 1392528 | 5188618 |
| 11 |  | 5410714 | -2788361 | 2622353 | 0 | 2622353 | 354073 | 2268281 | 1326217 | 6514835 |
| 12 |  | 5410714 | -2788361 | 2622353 | 0 | 2622353 | 655588 | 1966765 | 1095168 | 7610003 |
| 13 |  | 5410714 | -2788361 | 2622353 | 0 | 2622353 | 655588 | 1966765 | 1043017 | 8653020 |
| 14 |  | 5410714 | -2788361 | 2622353 | 0 | 2622353 | 655588 | 1966765 | 993350 | 9646370 |
| 15 |  | 5410714 | -2788361 | 2622353 | 0 | 2622353 | 655588 | 1966765 | 946048 | 10592418 |
| 16 |  | 5410714 | -2788361 | 2622353 | 0 | 2622353 | 655588 | 1966765 | 900998 | 11493416 |
| 17 |  | 5410714 | -2788361 | 2622353 | 0 | 2622353 | 655588 | 1966765 | 858093 | 12351509 |
| 18 |  | 5410714 | -2788361 | 2622353 | 0 | 2622353 | 655588 | 1966765 | 817231 | 13168740 |
| 19 |  | 5410714 | -2788361 | 2622353 | 0 | 2622353 | 655588 | 1966765 | 778316 | 13947056 |
| 20 | 603031 | 5410714 | -2788361 | 2622353 | 0 | 2622353 | 655588 | 2569796 | 968529 | 14915585 |

**Reference**

[1] a) G. Kresse, J. Furthmüller, *Comput. Mater. Sci.* **1996**, 6, 15; b) G. Kresse, J. Furthmüller, *Phys. Rev. B* **1996**, 54, 11169.

[2] J. P. Perdew, K. Burke, M. Ernzerhof, *Phys. Rev. Lett.* **1996**, 77, 3865.

[3] G. Kresse, D. Joubert, *Phys. Rev. B* **1999**, 59, 1758.

[4] H. J. Monkhorst, J. D. Pack, *Phys. Rev. B* **1976**, 13, 5188.

[5] I. Bagemihl, L. Cammann, M. Pérez-Fortes, V. van Steijn, J. R. van Ommen, *ACS Sustain. Chem. Eng.* **2023**, 11, 10130.

[6] N. R. Sukor, A. H. Shamsuddin, T. M. I. Mahlia, M. F. Mat Isa, *Processes* **2020**, 8, 350.

[7] A. Fivga, I. Dimitriou, *Energy* **2018**, 149, 865.

[8] a) W. D. Seider, D. R. Lewin, J. Seader, S. Widagdo, R. Gani, K. M. Ng, *Product and process design principles: synthesis, analysis and evaluation*, John Wiley & Sons, **2016**, ISBN 1119282632; b) G. Towler, R. Sinnott, *Chemical engineering design: principles, practice and economics of plant and process design*, Butterworth-Heinemann, **2021**, ISBN 0323850480.

[9] Q. Xia, C. Chen, T. Li, S. He, J. Gao, X. Wang, L. Hu, *Sci. Adv.* **2021**, 7, eabd7342.

[10] H. Chen, J. Yu, L. Liu, R.-T. Gao, Z. Gao, Y. Yang, Z. Chen, S. Zhan, X. Liu, X. Zhang, H. Dong, L. Wu, L. Wang, *Adv. Energy Mater.* **2024**, 14, 2303635.

[11] P. V. Sarma, T. V. Vineesh, R. Kumar, V. Sreepal, R. Prasannachandran, A. K. Singh, M. M. Shaijumon, *ACS Catal.* **2020**, 10, 6753.

[12] a) L. Xie, L. Wang, X. Liu, J. Chen, X. Wen, W. Zhao, S. Liu, Q. Zhao, *Nat. Commun.***2024**, 15, 5702; b) B. Yan, X. Qin, T. Chen, Z. Teng, D. K. Cho, H. W. Lim, H. Hong, Y. Piao, L. Xu, J. Y. Kim, *Adv. Funct. Mater.* **2024**, 34, 2309264.

[13] a) H. Sun, B. Yao, Y. Han, L. Yang, Y. Zhao, S. Wang, C. Zhong, J. Chen, C.-P. Li, M. Du, *Adva. Energy Mater.* **2024**, 14, 2303563; b) A. Li, X. Tang, R. Cao, D. Song, F. Wang, H. Yan, H. Chen, Z. Wei, *Adv. Mater.* **2024**, 36, 2401818.

[14] J. Liang, Z. Cai, Z. Li, Y. Yao, Y. Luo, S. Sun, D. Zheng, Q. Liu, X. Sun, B. Tang, *Nat. Commun.* **2024**, 15, 2950.

[15] H. Hu, X. Wang, Z. Zhang, J. Liu, X. Yan, X. Wang, J. Wang, J. P. Attfield, M. Yang, *Adv. Mater.* **2024**, 37, 2415421.

[16] X. Tian, R. Liu, W. Wang, Q. Yang, Z. Huang, Y. Yang, J. Han, T. Dong, Y. Du, J. Lai, H. Li, L. Wang, *Adv. Mater.* **2025**, e06068.

[17] J. Wang, Y. Sun, Y. Cui, H. Qiu, *Energy Environ. Sci.* **2025**, 18, 1756.

[18] P. Cui, T. Wang, X. Zhang, X. Wang, H. Wu, Y. Wu, C. Ba, Y. Zeng, P. Liu, J. Jiang, *ACS Nano* **2023**, 17, 22268.

[19] H. Shi, Y.-T. Zhou, R.-Q. Yao, W.-B. Wan, X. Ge, W. Zhang, Z. Wen, X.-Y. Lang, W.-T. Zheng, Q. Jiang, *Nat. Commun.* **2020**, 11, 2940.

[20] L. Huang, R. Yao, X. Wang, S. Sun, X. Zhu, X. Liu, M. G. Kim, J. Lian, F. Liu, Y. Li, H. Zong, S. Han, X. Ding, *Energy Environ. Sci.* **2022**, 15, 2425.

[21] Z. Wei, M. Guo, Q. Zhang, *Appl. Catal. B-Environ.* **2023**, 322, 122101.

[22] S. Zhang, C. Tan, R. Yan, X. Zou, F. L. Hu, Y. Mi, C. Yan, S. Zhao, *Angew. Chem. Int. Ed.* **2023**, 62, e202302795.

[23] R. Wang, X. Sun, J. Zhong, S. Wu, Q. Wang, K. Ostrikov, *Appl. Catal. B-Environ. Energy* **2024**, 352, 124027.

[24] H. Wu, Z. C. Wang, Z. X. Li, Y. J. Ma, F. Ding, F. Q. Li, H. F. Bian, Q. X. Zhai, Y. L. Ren, Y. X. Shi, Y. R. Yang, Y. Deng, S. C. Tang, X. K. Meng, *Adv. Energy Mater.* **2023**, 13, 2300837.

[25] K. Dastafkan, X. Shen, R. K. Hocking, Q. Meyer, C. Zhao, *Nat. Commun.***2023**, 14, 547.

[26] Y. Zhang, S. Lee, S. Jeong, E. Son, J. M. Baik, Y. K. Han, H. Park, *Adv. Funct. Mater.* **2023**, 34, 2309250.

[27] F.-C. Pan, J. Jia, F. Gong, Y. Liu, S. Liu, S. C. Jun, D. Lin, Y. Guo, Y. Yamauchi, Y. Huo, *ACS Nano* **2024**, 18, 6202.

[28] X. Teng, Z. Wang, Y. Wu, Y. Zhang, B. Yuan, Y. Xu, R. Wang, A. Shan, *Nano Energy* **2024**, 122, 109299.

[29] S. Chandrasekaran, T. T. Ma, Z. C. Hu, Q. Y. Liu, C. Zhan, Y. Li, C. Bowen, H. D. Lu, Y. P. Liu, *Appl. Catal. B-Environ.* **2023**, 338, 123007.

[30] L. Yang, X. Cao, X. Wang, Q. Wang, L. Jiao, *Appl. Catal. B-Environ.* **2023**, 329, 122551.

[31] M. Li, X. Wang, K. Liu, Z. Zhu, H. Guo, M. Li, H. Du, D. Sun, H. Li, K. Huang, Y. Tang, G. Fu, *Adv. Energy Mater.***2023**, 13, 2301162.

[32] J.-T. Ren, L. Chen, H.-Y. Wang, W.-W. Tian, X.-L. Song, Q.-H. Kong, Z.-Y. Yuan, *ACS Catal.* **2023**, 13, 9792.

[33] W. Zhang, L. Yang, Z. Li, G. Nie, X. Cao, Z. Fang, X. Wang, S. Ramakrishna, Y. Long, L. Jiao, *Angew. Chem. Int. Ed.* **2024**, 63, e202400888.

[34] X. Yu, F. Yan, Y. Zhao, B. Geng, X. Ma, L. Wu, X. Zhang, Y. Chen, *Appl. Catal.B-Environ.* **2024**, 343, 123534.

[35] W. Zhai, Y. Chen, Y. Liu, Y. Ma, P. Vijayakumar, Y. Qin, Y. Qu, Z. Dai, *Nano-Micro Letters* **2024**, 16, 115.

[36] H. F. Du, T. F. Wang, S. He, B. X. Li, K. Wang, Q. Chen, Z. Z. Du, W. Ai, W. Huang, *Adv. Funct. Mater.* **2024**, 34, 2311854.

[37] S. Wang, C. Z. Yuan, Y. S. Zheng, Y. Kang, K. S. Hui, K. X. Wang, H. X. Gao, D. A. Dinh, Y. R. Cho, K. N. Hui, *ACS Catal.* **2024**, 14, 3616.

[38] L. Lin, C. Zhang, C. Liang, H. Zhang, Z. Wang, P. Wang, Z. Zheng, H. Cheng, D. Xing, Y. Dai, Y. Liu, B. Huang, *Adv. Mater.***2024**, 36, 2402388.

[39] K. Yeom, J. Jo, H. Shin, H. Ji, S. Moon, J. E. Park, S. Lee, J. Shim, D. H. Mok, M. S. Bootharaju, S. Back, T. Hyeon, Y.-E. Sung, *Adv. Funct. Mater.***2024**, 34, 2401095.

[40] S. Li, T. Liu, W. Zhang, M. Wang, H. Zhang, C. Qin, L. Zhang, Y. Chen, S. Jiang, D. Liu, X. Liu, H. Wang, Q. Luo, T. Ding, T. Yao, *Nat. Commun.* **2024**, 15, 3416.

[41] Y. Li, Z. Zhang, C. Li, Y. Zhou, X.-B. Chen, H. Lu, Z. Shi, S. Feng, *Appl. Catal. B-Environ. Energy* **2024**, 355, 124116.

[42] N. Zhang, Y. Hu, L. An, Q. Li, J. Yin, J. Li, R. Yang, M. Lu, S. Zhang, P. Xi, C. H. Yan, *Angew. Chem. Int. Ed.* **2022**, 61, e202207217.

[43] J. Zhou, F. Qiao, Z. C. Ren, X. B. Hou, Z. K. Chen, S. X. Dai, G. Su, Z. W. Cao, H. Q. Jiang, M. H. Huang, *Adv. Funct. Mater.***2024**, 34, 2304380.

[44] C. He, L. Yang, X. Peng, S. Liu, J. Wang, C. Dong, D. Du, L. Li, L. Bu, X. Huang, *ACS Nano* **2023**, 17, 5861.

[45] B. Wang, J. Li, D. Li, J. Xu, S. Liu, Q. Jiang, Y. Zhang, Z. Duan, F. Zhang, *Adv. Mater.* **2024**, 36, 2305437.

[46] X.-L. Wang, L. Sun, L. Yang, J. Zhao, Q. Xu, *Adv. Funct. Mater.* **2024**, 34, 2314247.

[47] K. D. Tran, T. H. Nguyen, D. T. Tran, V. A. Dinh, N. H. Kim, J. H. Lee, *ACS Nano* **2024**, 18, 16222.

[48] J. Zhang, T. Quast, W. He, S. Dieckhofer, J. R. C. Junqueira, D. Ohl, P. Wilde, D. Jambrec, Y. T. Chen, W. Schuhmann, *Adv. Mater.***2022**, 34, e2109108.

[49] Y. Hu, Y. Zheng, J. Jin, Y. Wang, Y. Peng, J. Yin, W. Shen, Y. Hou, L. Zhu, L. An, M. Lu, P. Xi, C.-H. Yan, *Nat. Commun.* **2023**, 14, 1949.

[50] K. L. Wu, X. Wang, W. Q. Wang, Y. Luo, W. Cao, Y. Y. Cao, H. J. Xie, Y. Yan, H. J. Lin, J. X. Zhu, K. Rui, *Adv.Funct. Mater.* **2023**, 33, 2214075.

[51] T. H. Nguyen, P. K. L. Tran, D. T. Tran, V. A. Dinh, N. H. Kim, J. H. Lee, *Appl. Catal. B-Environ.***2024**, 343, 123517.

[52] K.-Y. Yoon, K.-B. Lee, J. Jeong, M.-J. Kwak, D. Kim, H. Y. Roh, J.-H. Lee, S. M. Choi, H. Lee, J. Yang, *ACS Catal.* **2024**, 14, 4453.

[53] S. Han, J. Park, J. Yoon, *Adv. Funct. Mater.* **2024**, 34, 2314573.

[54] L. Q. Wang, M. Y. Ma, C. C. Zhang, H. H. Chang, Y. Zhang, L. L. Li, H. Y. Chen, S. J. Peng, *Angew. Chem. Int. Ed.***2024**, 63, e202317220.

[55] W. Jiang, A. Y. Faid, B. F. Gomes, I. Galkina, L. Xia, C. M. S. Lobo, M. Desmau, P. Borowski, H. Hartmann, A. Maljusch, A. Besmehn, C. Roth, S. Sunde, W. Lehnert, M. Shviro, *Adv. Funct. Mater.* **2022**, 32, 2203520.

[56] S. Jo, J. I. Jeon, K. H. Shin, L. Zhang, K. B. Lee, J. Hong, J. I. Sohn, *Adv.* *Mater.* **2024**, 36, 2314211.

[57] R. Yao, K. Sun, K. Zhang, Y. Wu, Y. Du, Q. Zhao, G. Liu, C. Chen, Y. Sun, J. Li, *Nat. Commun.* **2024**, 15, 2218.

[58] P. Yang, F. Liu, X. Zang, L. Xin, W. Xiao, G. Xu, H. Li, Z. Li, T. Ma, J. Wang, Z. Wu, L. Wang, *Adv. Energy Mater.* **2024**, 14, 2303384.

[59] H.-J. Niu, C. Huang, T. Sun, Z. Fang, X. Ke, R. Zhang, N. Ran, J. Wu, J. Liu, W. Zhou, *Angew. Chem. Int. Ed.* **2024**, 63, e202401819.

[60] L. Zeng, Z. Zhao, Q. Huang, C. Zhou, W. Chen, K. Wang, M. Li, F. Lin, H. Luo, Y. Gu, L. Li, S. Zhang, F. Lv, G. Lu, M. Luo, S. Guo, *J. Am. Chem. Soc.* **2023**, 145, 21432.

[61] H. Wang, X. Liu, G. Liu, Y. Wang, X. Du, J. Li, *Chem Catal.* **2023**, 3, 100552.

[62] P. Zhai, C. Wang, Y. Zhao, Y. Zhang, J. Gao, L. Sun, J. Hou, *Nat. Commun.* **2023**, 14, 1873.

[63] L. Gao, F. Bao, X. Tan, M. Li, Z. Shen, X. Chen, Z. Tang, W. Lai, Y. Lu, P. Huang, C. Ma, S. C. Smith, Z. Ye, Z. Hu, H. Huang, *Energy Environ. Sci.* **2023**, 16, 285.

[64] X. Bai, M. Zhang, Y. Shen, X. Liang, W. Jiao, R. He, Y. Zou, H. Chen, X. Zou, *Adv. Funct. Mater.* **2024**, 34, 2400979.

[65] M. H. Wang, Z. X. Lou, X. Wu, Y. Liu, J. Y. Zhao, K. Z. Sun, W. X. Li, J. Chen, H. Y. Yuan, M. Zhu, S. Dai, P. F. Liu, H. G. Yang, *Small* **2022**, 18, 2200303.

[66] D. Cao, Z. Zhang, Y. Cui, R. Zhang, L. Zhang, J. Zeng, D. Cheng, *Angew. Chem. Int. Ed.* **2023**, 62, e202214259.

[67] H. Lei, Q. Wan, S. Tan, Z. Wang, W. Mai, *Adv. Mater.* **2023**, 35, e2208209.

[68] Worldwide Tax Summaries, https://taxsummaries.pwc.com/.

[69] IRENA (**2020**), *Green Hydrogen Cost Reduction: Scaling up Electrolysers to Meet the 1.5⁰C Climate Goal*, International Renewable Energy Agency, Abu Dhabi.

[70] a) A. Ozden, Y. Wang, F. Li, M. Luo, J. Sisler, A. Thevenon, A. Rosas-Hernández, T. Burdyny, Y. Lum, H. Yadegari, T. Agapie, J. C. Peters, E. H. Sargent, D. Sinton, *Joule* **2021**, 5, 706; b) X. Wang, Y. Chen, F. Li, R. K. Miao, J. E. Huang, Z. Zhao, X.-Y. Li, R. Dorakhan, S. Chu, J. Wu, S. Zheng, W. Ni, D. Kim, S. Park, Y. Liang, A. Ozden, P. Ou, Y. Hou, D. Sinton, E. H. Sargent, *Nat. Commun.* **2024**, 15, 616.

[71] R. P. Bangalore Ashok, P. Oinas, S. Forssell, *Renew. Energy* **2022**, 190, 396.

[72] J. Qi, Y. Du, Q. Yang, N. Jiang, J. Li, Y. Ma, Y. Ma, X. Zhao, J. Qiu, *Nat. Commun.* **2023**, 14, 6263.

[73] X. Wang, P. Ou, A. Ozden, S.-F. Hung, J. Tam, C. M. Gabardo, J. Y. Howe, J. Sisler, K. Bertens, F. P. García de Arquer, R. K. Miao, C. P. O’Brien, Z. Wang, J. Abed, A. S. Rasouli, M. Sun, A. H. Ip, D. Sinton, E. H. Sargent, *Nat. Energy* **2022**, 7, 170.

[74] B. Pribyl-Kranewitter, A. Beard, C. L. Gîjiu, D. Dinculescu, T. J. Schmidt, *Renew. Sustain. Energy Rev.* **2022**, 154, 111807.
